# Macrophage Migration Inhibitory Factor Promotes Thromboinflammation and Predicts Fast Progression of Aortic Stenosis

**DOI:** 10.1161/ATVBAHA.124.321000

**Published:** 2024-07-11

**Authors:** Karin Anne Lydia Mueller, Carolin Langnau, Tobias Harm, Manuel Sigle, Kristina Mott, Michal Droppa, Oliver Borst, Anne-Katrin Rohlfing, Sarah Gekeler, Manina Günter, Nora Goebel, Ulrich F.W. Franke, Medhat Radwan, Christian Schlensak, Henrik Janning, Sophia Scheuermann, Christian M. Seitz, Dominik Rath, Klaus-Peter Kreisselmeier, Tatsiana Castor, Iris Irmgard Mueller, Harald Schulze, Stella E. Autenrieth, Meinrad Paul Gawaz

**Affiliations:** 1 Department of Cardiology and Angiology (K.A.L.M., C.L., T.H., M.S., M.D., O.B., A.-K.R., S.G., H.J., D.R., K.-P.K., T.C., I.I.M., M.P.G.), University Hospital Tuebingen, Eberhard Karls University Tuebingen, Germany.; 2 Department of Hematology, Oncology, Clinical Immunology and Rheumatology (M.G., S.E.A.), University Hospital Tuebingen, Eberhard Karls University Tuebingen, Germany.; 3 Department of Thoracic and Cardiovascular Surgery (M.R., C.S.), University Hospital Tuebingen, Eberhard Karls University Tuebingen, Germany.; 4 Institute for Experimental Biomedicine, Chair I University Hospital Würzburg, Germany (K.M., H.S.).; 5 DFG Heisenberg Group Thrombocardiology (O.B.), University of Tübingen, Germany.; 6 Cluster of Excellence iFIT (EXC 2180) Image-Guided and Functionally Instructed Tumor Therapies (S.S., C.M.S.), University of Tübingen, Germany.; 7 Dendritic Cells in Infection and Cancer, German Cancer Research Center (DKFZ), Heidelberg, Germany (M.G., S.E.A.).; 8 Robert-Bosch Hospital, Department of Cardiovascular Surgery, Stuttgart, Germany (N.G., U.F.W.F.).; 9 Department of Pediatric Hematology and Oncology, University Children’s Hospital Tuebingen, Germany (S.S., C.M.S.).

**Keywords:** aortic valve stenosis, biomarkers, blood platelets, chemokines, inflammation

## Abstract

**BACKGROUND:**

Aortic stenosis (AS) is driven by progressive inflammatory and fibrocalcific processes regulated by circulating inflammatory and valve resident endothelial and interstitial cells. The impact of platelets, platelet-derived mediators, and platelet-monocyte interactions on the acceleration of local valvular inflammation and mineralization is presently unknown.

**METHODS:**

We prospectively enrolled 475 consecutive patients with severe symptomatic AS undergoing aortic valve replacement. Clinical workup included repetitive echocardiography, analysis of platelets, monocytes, chemokine profiling, aortic valve tissue samples for immunohistochemistry, and gene expression analysis.

**RESULTS:**

The patients were classified as fast-progressive AS by the median ∆Vmax of 0.45 m/s per year determined by echocardiography. Immunohistological aortic valve analysis revealed enhanced cellularity in fast-progressive AS (slow- versus fast-progressive AS; median [interquartile range], 247 [142.3–504] versus 717.5 [360.5–1234]; *P*<0.001) with less calcification (calcification area, mm^2^: 33.74 [27.82–41.86] versus 20.54 [13.52–33.41]; *P*<0.001). MIF (macrophage migration inhibitory factor)-associated gene expression was significantly enhanced in fast-progressive AS accompanied by significantly elevated MIF plasma levels (mean±SEM; 6877±379.1 versus 9959±749.1; *P*<0.001), increased platelet activation, and decreased intracellular MIF expression indicating enhanced MIF release upon platelet activation (CD62P, %: median [interquartile range], 16.8 [11.58–23.8] versus 20.55 [12.48–32.28], *P*=0.005; MIF, %: 4.85 [1.48–9.75] versus 2.3 [0.78–5.9], *P*<0.001). Regression analysis confirmed that MIF-associated biomarkers are strongly associated with an accelerated course of AS.

**CONCLUSIONS:**

Our findings suggest a key role for platelet-derived MIF and its interplay with circulating and valve resident monocytes/macrophages in local and systemic thromboinflammation during accelerated AS. MIF-based biomarkers predict an accelerated course of AS and represent a novel pharmacological target to attenuate progression of AS.

**Highlights**

- Patients with fast-progressive aortic stenosis show an enhanced systemic thromboinflammation with specific platelet and monocyte phenotypes that correlate with the degree of local aortic valve inflammation.
- Our study reveals a prominent inflammatory valvular phenotype in patients with fast-progressive aortic valve stenosis.
- Transcriptomic analysis of valve tissue, chemokine profiling, and multicolor flow cytometry of circulating platelets and monocytes highlight that MIF (macrophage migration inhibitory factor)-regulated pathways are critically involved in disease progression.
- Our findings suggest a novel role of platelets and the platelet-derived mediator MIF as another key component in the complex mechanisms of degenerative aortic valve disease.

**See cover image**

Aortic stenosis (AS) is the most prevalent heart valve pathology worldwide, especially in the aging population, and is associated with a poor prognosis once symptoms occur.^1^ AS is defined as a progressive disease with increasing hemodynamic severity over time. Several recent studies have defined the clinical progress of AS by its echocardiographic parameters and their change during disease progression. To distinguish between fast-progressive AS (FP-AS) and slow-progressive AS (SP-AS), the change of the peak velocity over the aortic valve (AV; ∆Vmax) or the decrease of the AV area within 1 year has been proposed.^2–7^ However, the underlying mechanisms triggering fast disease progression remain unclear, and patients at risk for fast progression are not well defined.^8–10^ Accelerating inflammation, fibrotic and calcific remodeling, and finally osteogenic formation of the AV cusps leads to valve obstruction^11,12^ followed by surgical or transcatheter AV replacement.^8,9^ No pharmacotherapy has been established to prevent the development and progression of degenerative AV disease (DAVD) resulting in AS; however, clinical trials targeting inflammatory and calcium metabolic pathways are ongoing.^13–15^ Thus, none of the suggested therapeutic strategies addresses early stages of DAVD to inhibit acceleration of inflammatory, fibrotic, or osteogenic formation and thereby improve prognosis and shift AV replacement to the latest time point possible.^13^ Therefore, the development of pharmacological treatment strategies targeting early regulatory mechanisms of inflammation still is an unmet clinical need.

Presumed underlying cellular and molecular pathophysiology is complex and comprises mechanical stress, endothelial damage, dysfunction of valve resident endothelial cells followed by lipid accumulation, and differentiation of valve interstitial cells (VICs) to myofibroblasts and inflammatory or calcifying phenotypes.^1,11,13^ Furthermore, circulating immune cells are activated and subsequently infiltrate valve tissue and accelerate myofibroblastic and osteoblastic differentiation of VICs thereby advancing tissue calcification.^13,16,17^ The differentiation of VICs into myofibroblastic/inflammatory or osteoblastic phenotypes is a pivotal step during the propagation phase of AS and seems to be regulated by various cytokines/chemokines and extracellular vesicles that are secreted by platelets and immune cells.^18^ Therefore, we speculate that platelet- and monocyte-derived proinflammatory mediators can induce the phenotypic change of VICs toward inflammatory and calcific remodeling of AV tissue.^1,12,13^ Platelets have been well recognized to play a critical role in vascular inflammation but also seem to regulate important processes in AS.^17,19,20^ Upon adhesion, platelets are activated and secrete a variety of inflammatory mediators like the cytokine-like chemokine MIF (macrophage migration inhibitory factor), a proinflammatory and proatherogenic regulator protein, which boosts vascular inflammation.^21^ MIF facilitates monocyte chemotaxis and infiltration and might be a potential trigger molecule for valve inflammation.^13,22,23^ Interactions of platelets and monocytes/macrophages^17,24,25^ and their inflammatory mediators might predominantly accelerate local inflammation in DAVD, and their role in underlying pathomechanisms needs to be clarified. Early inhibition of platelet adhesion via GP (glycoprotein) Ibα or GP IIb results in decrease of AV inflammation, reduction of leukocyte accumulation, and atherosclerotic lesion formation of AVs in mouse models.^26^ Inhibition of platelets by conventional antiplatelet drugs decreases platelet aggregation but does not substantially reduce secretion of platelet-derived mediators and might, therefore, not effectively prevent progression in DAVD.^13,17,20^ Targeting key compounds in valvular inflammation, however, might be a more promising strategy.^20^ Identifying enhanced systemic inflammatory regulators like elevated MIF plasma levels may help to determine patients at risk for progressive AS before symptoms due to severe critical AS occur. Targeting MIF may be a promising target molecule in prevention of AS progression.^22,23^

Thus, we hypothesized that changes in platelet activity, platelet-derived mediators, and platelet-monocyte interactions in systemic thromboinflammation in AS are of prognostic relevance for an accelerated course of the disease leading to fast progression as defined by repetitive echocardiography.

## MATERIALS AND METHODS

**Figure 1.**
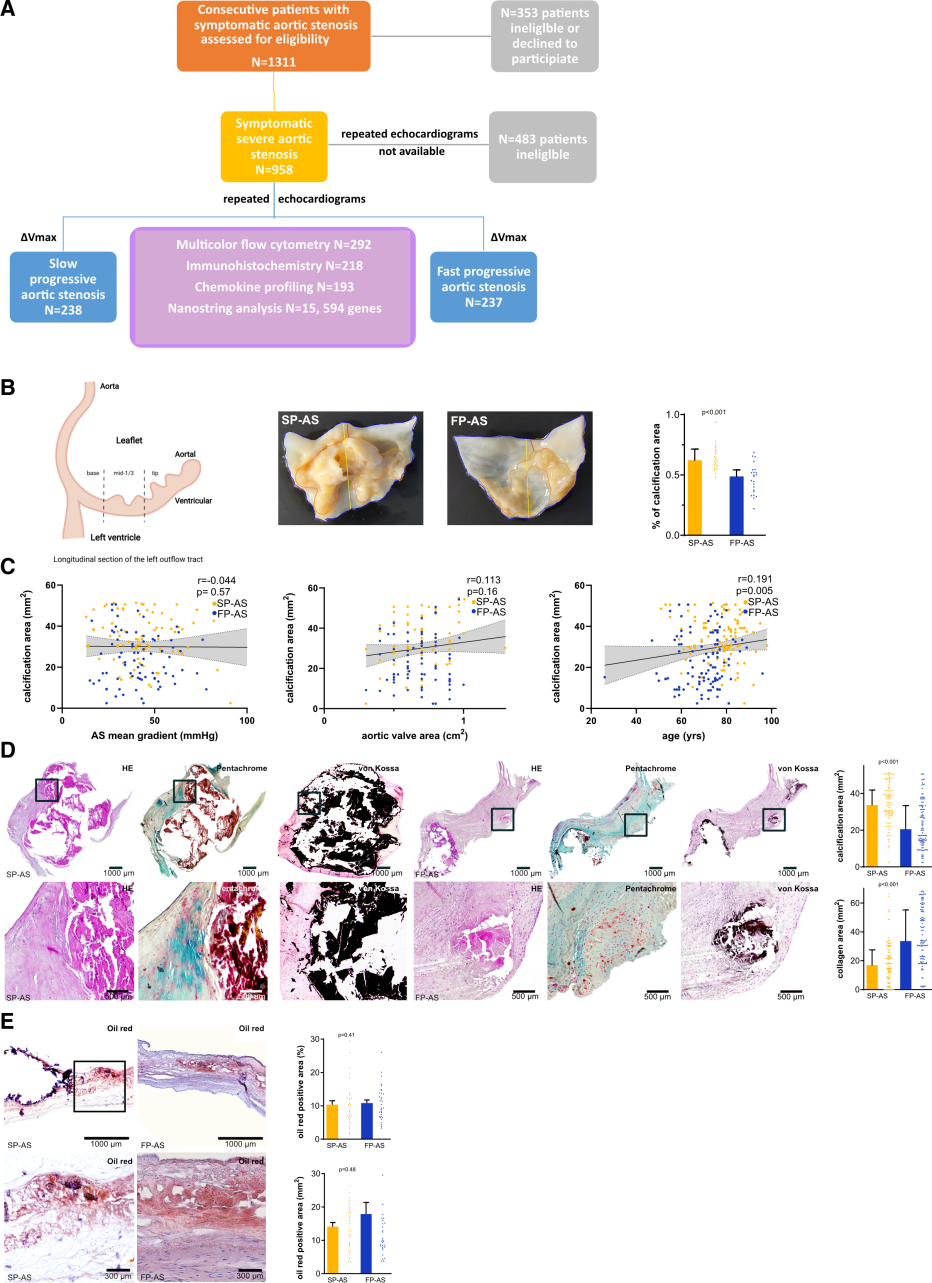
**Study design and histological characteristics of valvular phenotypes in slow-progressive aortic stenosis (SP-AS) and fast-progressive aortic stenosis (FP-AS). A**, Study flowchart. **B**, Morphological analysis of patients with either SP-AS (n=22) or FP-AS (n=23) in gross pathology regarding the degree of mineralization and calcification. **C**, Spearman correlation analysis of SP-AS (n=238) and FP-AS (n=237). **D**, Representative histological stainings of SP-AS (n=110) and FP-AS (n=108) showing calcification area (mm^2^) and collagen area (mm^2^). **E**, Oil red O–positive area (%, mm^2^) in SP-AS (n=32) and FP-AS (n=32), respectively. Plotted: median±interquartile range; statistics: Mann-Whitney *U* test. HE indicates hematoxylin and eosin.

**Figure 2.**
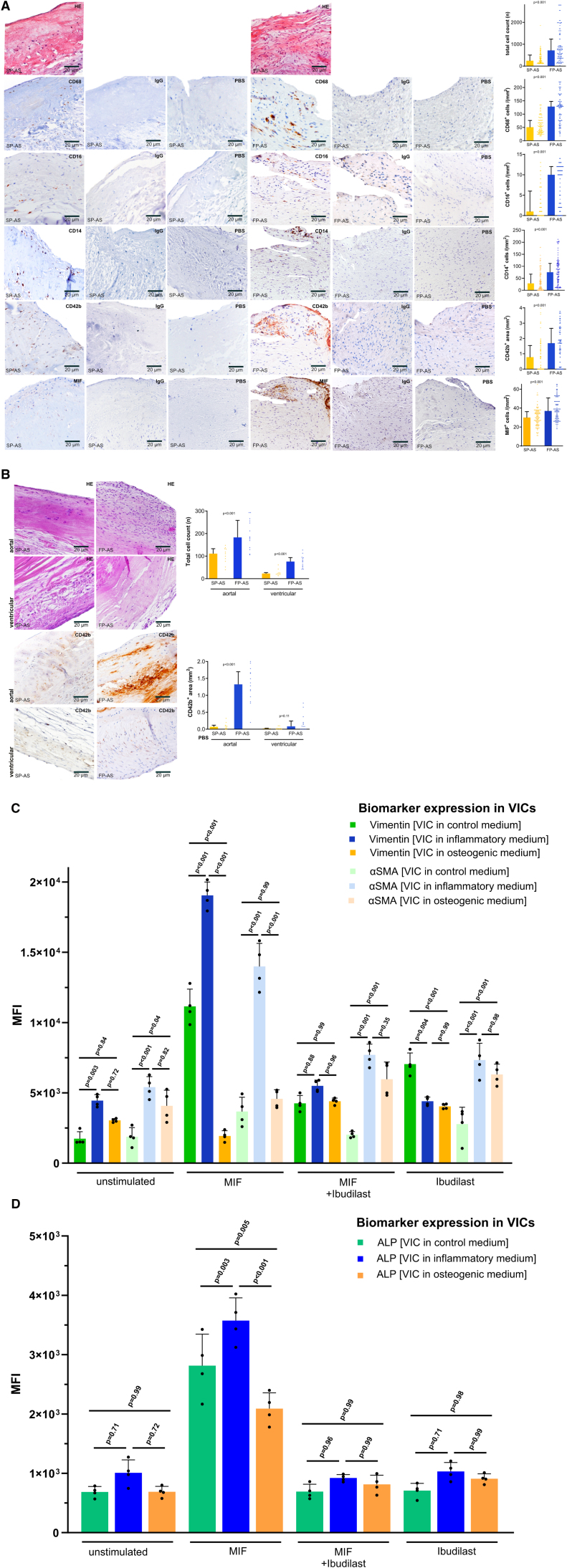
**Fast-progressive aortic stenosis (FP-AS) and slow-progressive aortic stenosis (SP-AS) show typical valvular expression patterns of valve infiltrating inflammatory cells, platelets, and macrophage MIF (macrophage migration inhibitory factor). A**, Representative immunohistological stainings of SP-AS (n=110) and FP-AS (n=108) to calculate the total aortic valve cell count (n), CD68^+^ macrophages, CD16^+^ monocytes, CD14^+^ monocytes, CD42b^+^ platelets, and MIF^+^ staining in matching areas of consecutive aortic valve (AV) tissue sections. Plotted: median±interquartile range (IQR); statistics: Mann-Whitney *U* test. **B**, Representative immunohistological stainings comparing aortal and ventricular aortic valve side in SP-AS (n=11) and FP-AS (n=14) to analyze the cell count (n) and CD42b^+^ positive areas in the aortal and the ventricular side of the AV. Plotted: median±IQR; statistics: Kruskal-Wallis test. **C**, In vitro analysis of cell culture of valvular interstitial cells (VICs) in control, proinflammatory, and pro-osteogenic medium. Vimentin and αSMA expression on VICs in control, proinflammatory, and pro-osteogenic medium without stimulation, under stimulation with MIF, coincubation with MIF and its antagonist ibudilast, and under inhibition by ibudilast. Plotted: mean±SD, n=4; statistics: 1-way ANOVA. Tukey multiple comparison test (*P*<0.05) was used to correct for multiple comparisons. **D**, ALP (alkaline phosphatase) expression on VICs in control, proinflammatory, and pro-osteogenic medium without stimulation, understimulation with MIF, coincubation with MIF and its antagonist ibudilast, and under inhibition by ibudilast. Proinflammatory and pro-osteogenic medium mimic an inflammatory (FP-AS) or calcifying (SP-AS) valvular and systemic phenotype as described before. Plotted: mean±SD, n=4; statistics: 1-way ANOVA. Tukey multiple comparison test (*P*<0.05) was used to correct for multiple comparisons. HE indicates hematoxylin and eosin.

Because of the sensitive nature of the data collected for this study, requests to access the data set from qualified researchers trained in human subject confidentiality protocols may be sent to the corresponding author. Detailed descriptions of study design, patient cohort (Figure 1A; Figure S1), blood sampling and sample preparation, valvular tissue procurement (Figures S2 and S3), in vitro cell culture experiments with VICs (Figure 2C and 2D), multicolor flow cytometry (Figures S4 through S9), chemokine profiling, RNA gene expression analysis by Nanostring technology, morphological analysis by computed tomography scans (Figure S10), and testing of confounding factors by PLS-DA (partial least-squares discriminant analysis) and OPLS-DA (orthogonal partial least squares discriminant analysis) (Figures S11 through S13) are provided in the Supplemental Material. All experiments of patient samples were performed in a blinded manner by 2 experienced investigators to ensure the repeatability of measurements.

The study was performed in accordance with the Declaration of Helsinki and local regulatory authorities (proposal number 240/2018B02). Written informed consent was obtained from every patient.

In brief, from December 2014 through March 2021, we prospectively enrolled 475 consecutive patients with severe symptomatic (New York Heart Association class ≥2) AS with indication for AV repair who presented at the Department of Cardiology and Angiology or at the Department of Heart Surgery of the University Hospital Tübingen, Germany (Figure 1A). All patients underwent clinical and cardiac examination including echocardiography, electrocardiography, assessment of medical history at baseline, concomitant medication, comorbidities, and blood sampling for routine laboratory parameters, marker expression on platelets and monocytes, as well as chemokine profiling. Tissue samples of explanted AVs were obtained from patients who underwent surgical valve replacement and were analyzed by histology, immunohistochemistry, and gene expression analysis using Nanostring technology. In our prospective study, a sample size of 262 patients was needed to detect a 10% increase of MIF^+^ monocytes (MFI, 1×10^4^ in patients with SP-AS) in patients with FP-AS (MFI, 1.1×10^4^; SD=25%) given an α-error of 0.05 and a power of 0.9. Assuming a 10% error caused by sampling/material a total number of 288 patients was required. Echocardiographic assessment was performed in all patients according to current guidelines.^8,9^ The diagnosis and severity of AS was classified according to current guidelines.^8,9^ We defined 2 subgroups of patients with symptomatic AS regarding fast (FP-AS, n=237) and slow (SP-AS, n=238) disease progression by repetitive transthoracic echocardiographic assessment in a retrospective analysis of our digital echocardiographic database. Here, we determined disease progression by the change of the maximum transvalvular flow velocity (∆Vmax) over time and established an annualized progression rate ∆Vmax as described before.^2,4,5,8,9^ The median annualized ∆Vmax was used as a cutoff, and thereby 2 subgroups of patients were evident with either FP-AS (∆Vmax, ≥0.45 m/s per year; n=237) or SP-AS (∆Vmax, <0.45 m/s per year; n=238) until onset of severe symptoms occurred that warranted surgical valve replacement (Figure S1).^5^

Furthermore, in a subgroup of n=134 patients, computed tomography scan of the AV and the aorta was performed before valve replacement (Figure S10).

### Statistical Analysis

Statistical analysis of all clinical and laboratory data and characterization of platelet and monocyte phenotypes, biomarker expression, RNA analysis, and AV morphology was performed. Non-normally distributed continuous data are represented as median with interquartile range (IQR), and normally distributed continuous data are represented as mean with SD. To test for normality assumption, analyses of individual histograms of the residuals and in case of uncertainty, Shapiro-Wilk test were performed. Brown-Forsythe test was performed to screen for equal group variances where applicable. Equal variance assumption was satisfied in this study when the ratio of the larger variance to the smaller variance was not larger than 4. Thus, Mann-Whitney *U* test was performed for 2 group comparisons and Kruskal-Wallis test for multiple group comparisons of non-normally distributed variables. Normally distributed continuous variables were compared using the Student *t* test or 1-way ANOVA for comparison of ≥3 groups as appropriate. Bonferroni adjustment or Tukey multiple comparison test (*P*<0.05 and *P*<0.01, respectively) was used to correct for multiple comparisons where applicable. Categorical variables are represented as total numbers and proportions of participants, and comparison was performed using the χ^2^ test. Correlation analysis was calculated by Spearman rank correlation coefficient. Nanostring data were analyzed using JMP version 16.0.0 (SAS Institute, Cary, NC) and it is implemented in the Student *t* test. Results were log2 transformed, and fold change of means was plotted in scatter plot and volcano plot displaying significant (*P*<0.05) alterations between patients with FP-AS and SP-AS. To classify the significantly regulated changes, gene ontology and Kyoto Encyclopedia of Genes and Genomes pathway analysis were performed using PathfindR RStudio, version 1.4.1717 (RStudio, Inc, Boston, MA; R package PathfindR).^27^ Bonferroni greedy algorithm active subnetwork search method was used to detect significantly (*P*<0.01) enriched pathways. A hierarchical clustering of the top 50 differentially expressed genes was performed using the pheatmap R package.^28^ *Z* scores of means were colored according to upregulation in FP-AS (blue) or SP-AS (orange). Further, a heatmap with row-wise comparisons of Nanostring data was performed. Observed *Z* scores of genes belonging to the MIF pathway (purple) and TGF-β1 (transforming growth factor-β1) pathway (yellow) were plotted according to PathCards online database. To summarize correlations of important parameters in patients with FP-AS and SP-AS, a matrix was performed using the Corrplot R package, displaying correlations of clinical parameters alongside ex vivo assays.^29^ For the correlation matrix, missing data were replaced using a nearest neighbor imputation algorithm.^30^ Spearman ρ is colored, and color intensity and size are plotted proportional to correlation coefficients. Important findings in this study are displayed by chord diagram using the circlize R package.^31^ Significant (*P*<0.05) changes of clinical parameters and ex vivo data, as well as differentially expressed gene pathways between patients with FP-AS (blue) and SP-AS (orange) were presented and colored according to their assay. Unsupervised data analysis of flow cytometry was done using OMIQ data analysis software (Omiq, Inc, Santa Clara, CA). In brief, the data were manually gated and flowAI^32^ was run to check for any aberrant regions of the files. Subsequently, dimension reduction analysis was performed using uniform manifold approximation and projection to visualize the different subpopulations of the cells. Detailed description is given in the Supplemental Material. Linear regression analysis and cox regression analysis were performed to evaluate associations of possible confounding factors and to test whether cell-based MIF markers (MIF expression in platelets and MIF plasma levels) were independent predictors of FP-AS. In linear regression analysis, we calculated β-coefficients and provide both unstandardized and standardized coefficients in our statistical evaluation. Hazard ratios and CIs were calculated and analyzed in cox regression analysis. To further assess the effect of possible confounding factors on disease progression of AS, PLS-DA and OPLS-DA analyses were performed on the subcohorts of patients with AS in dependence on each possible clinical confounder. OPLS-DA and PLS-DA were performed using SIMCA by Umetrics, version 16.0 (Sartorius AG, Goettingen, Germany). Comparisons were considered statistically significant if 2-sided *P* value was ≤0.05. Statistical analysis was performed with either IBM SPSS Statistics software, version 26 (SPSS, Inc, Chicago, IL), GraphPad Prism, version 8.4.0, and 10.1.1 (323; GraphPad Software, Inc, Boston, MA), RStudio, version 1.4.1717 (RStudio, Inc, Boston, MA), SIMCA by Umetrics, version 16.0 (Sartorius AG), or JMP, version 16.0.0 (SAS Institute, Cary, NC), where applicable and as stated above.^27,33,34^

## RESULTS

### Accelerated AS (FP-AS) Is Characterized by Substantially Enhanced Infiltration of Inflammatory Cells and Platelets in Valve Tissue

To get a deeper insight into the pathophysiological mechanisms of FP-AS and SP-AS, we prospectively studied 475 consecutive patients admitted to our hospital for symptomatic AS with indication for valve replacement (Table; Table S1). We identified 2 subgroups of patients by repetitive transthoracic echocardiographic assessment as described in Methods (Figure S1). Hereby, 237 (49.9%) patients were classified as FP-AS by a median ∆Vmax of 0.45 m/s per year. Patients with FP-AS were younger (SP-AS versus FP-AS; median [IQR], 79 [71–83] versus 76 [69–81]; *P*<0.001), had a slightly lower risk score defined by the Society of Thoracic Surgeons,^8,9^ and showed a lower incidence of atrial fibrillation. Transvalvular flow velocity, AV area, and stroke volume were similar in both groups (Table; Table S1). In a subgroup of 134 patients, computed tomography scans of the AV were performed before valve replacement but revealed neither morphological differences regarding the degree of valve calcification nor regarding distribution patterns of calcified areas between FP-AS and SP-AS (Figure S10).

**Table.**
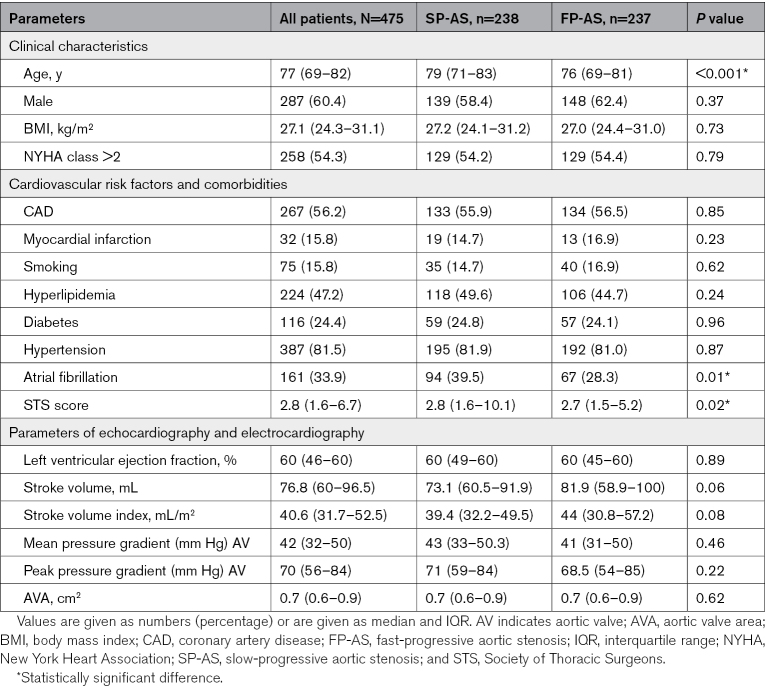
Baseline Characteristics of Patient Population

Assessment of gross pathology of the explanted valves revealed a significantly higher calcification grade of AV cusps in SP-AS (SP-AS versus FP-AS, %: median [IQR], 0.62 [0.56–0.71] versus 0.48 [0.36–0.54]; *P*<0.001; Figure 1B). The degree of macroscopic calcification did not correlate with echocardiographic parameters, while age was significantly associated with calcification areas in gross pathology (*P*=0.005; Figure 1C). Interestingly, histological analysis showed that collagen content (visualized by pentachrome staining) of AV tissue from FP-AS was significantly increased (SP-AS versus FP-AS, mm^2^: median [IQR], 16.91 [7.36–27.54] versus 33.66 [21.24–55.2]; *P*<0.001; Figure 1D). In contrast, mineralization (depicted by van Kossa staining) was significantly enhanced in SP-AS (SP-AS versus FP-AS, mm^2^: median [IQR], 33.74 [27.82–41.86] versus 20.54 [13.52–33.41]; *P*<0.001; Figure 1D). Lipid accumulation assessed by oil red O staining was not different between both groups (Figure 1E). The cellularity of AV tissue, especially that of CD14^+^ and CD16^+^ monocytes, CD68^+^ macrophages, and CD42^+^ platelets, was significantly increased in FP-AS compared with SP-AS (Figure 2A), suggesting an extravasation and infiltration of immune cells into the AV tissue (CD14^+^/CD16^+^/CD68^+^ cells in SP-AS versus FP-AS, respectively, cells/mm^2^: median [IQR]: 29 [7–67.3] versus 75.5 [36.3–112], *P*<0.001; 1 [0–6] versus 10 [2–12], *P*<0.001; 51 [32–76.25] versus 128.5 [75–147.8], *P*<0.001, respectively; platelet accumulation [CD42b^+^ area], mm^2^: 0.78 [0–1.54] versus 1.7 [0.77–2.66]; *P*<0.001). Topical analysis revealed that platelet and cellular infiltration occurs predominantly in the interstitial layer and on the aortic side, while substantially less on the ventricular side of the AV of FP-AS (cellularity, aortal: SP-AS versus FP-AS: 111 [77–133] versus 183 [135.8–258.5], *P*<0.001; cellularity, ventricular: SP-AS versus FP-AS: 23 [18–27] versus 76 [52.25–93.5], *P*<0.001; CD42b^+^ area, aortal: SP-AS versus FP-AS; in mm^2^; 0.07 [0.02–0.12] versus 1.33 [0.92–1.7], *P*<0.001; CD42b^+^ area, ventricular: SP-AS versus FP-AS; in mm^2^; 0.02 [0–0.03] versus 0.09 [0–0.24], *P*=0.11; Figure 2B). Among other bone marrow–derived blood cells, platelets have been recognized as a major source for MIF. MIF is stored in large quantities within granules and is rapidly released from activated platelets. In the areas of platelet infiltration, substantial immunoreactivity of MIF is found (Figure 2A and 2B), which is evidenced by immunofluorescence experiments (Figure S3). Minor MIF expression was found in colocalization with monocytes/macrophages. Thus, platelets seem to be the predominant but not exclusive source of MIF within the diseased AV tissue. Furthermore, these results indicate that FP-AS valve tissue is characterized by enhanced infiltration of inflammatory cells and platelets, as well as less calcification compared with SP-AS. To address the functional impact of MIF for AS progression, we performed additional in vitro cell culture experiments. We analyzed the effect of recombinant MIF and the MIF antagonist ibudilast on inflammatory and osteogenic changes of VICs as described in the Supplemental Material. We found that depending on the culture medium (inflammatory versus osteogenic), MIF enhances prominently an inflammatory phenotype of VICs with significantly increased vimentin and α-SMA expression, which could be attenuated by the MIF antagonist ibudilast (Figure 2C). As osteogenic differentiation of VICs is characterized by increased activity and expression of ALP (alkaline phosphatase), we also analyzed ALP expression in in vitro cell culture. Here, we detected that MIF stimulation also results in increased ALP expression in inflammatory-driven VICs compared with VICs in osteogenic medium (Figure 2D). Thus, we conclude that MIF is a major trigger for inflammatory AV disease progression.

### MIF and Its Related Pathways Are Predominant Regulators of Local Valve Tissue Inflammation in Patients With FP-AS

**Figure 3.**
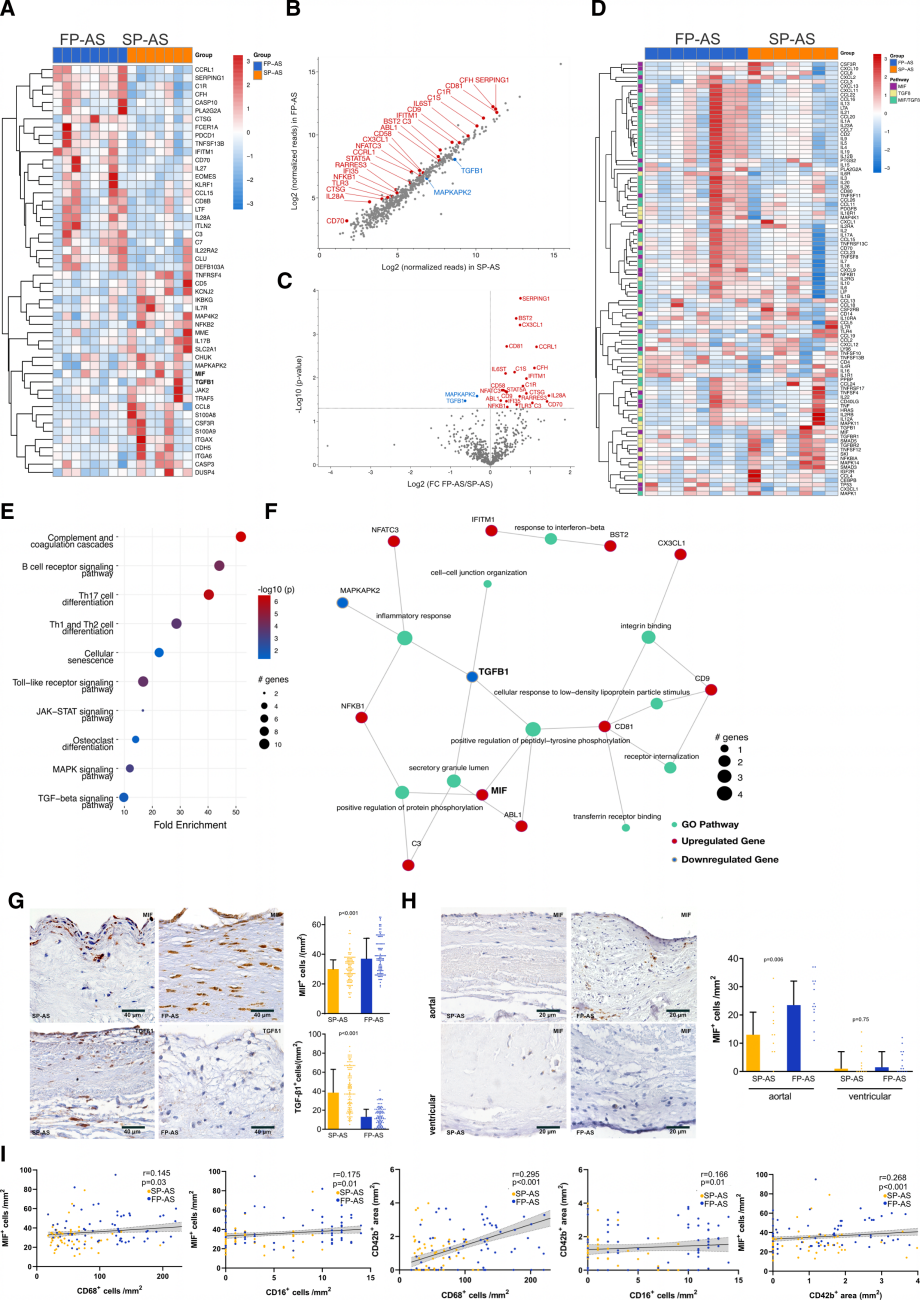
**MIF (macrophage migration inhibitory factor)-associated gene and protein expression in fast-progressive aortic stenosis (FP-AS). A**, Hierarchical clustering analysis of the top 50 differentially expressed genes of NanoString mRNA profiling including MIF and TGF-β1 (transforming growth factor-β1), *Z* scores of means indicating upregulation in FP-AS (blue, n=8) or slow-progressive aortic stenosis (SP-AS; orange, n=7). Scatter plot (**B**) and volcano plot (**C**) displaying significant (*P*<0.05) alterations in SP-AS (n=7) and FP-AS (n=8). **D**, Heatmap with row-wise comparisons of Nanostring data in patients with FP-AS (blue) and SP-AS (orange). *Z* scores of MIF (purple) and TGF-β1–associated pathways (yellow) according to PathCards online database. **E**, The top 10 significantly enriched Kyoto Encyclopedia of Genes and Genomes pathways subsuming the 25 significantly regulated genes in FP-AS were plotted. **F**, A term-gene-graph highlights subnetworks and regulations of significantly regulated genes and the referring pathway in FP-AS displaying MIF interactions with significantly (*P*<0.01) enriched Gene Ontology (GO) pathways. **G**, Representative immunohistological stainings of SP-AS (n=110) and FP-AS (n=108) show MIF^+^ and TGF-β1^+^ cells. Plotted: median±interquartile range (IQR); statistics: Mann-Whitney *U* test. **H**, Analysis of aortal and ventricular aortic valve side in SP-AS (n=11) and FP-AS (n=14). Plotted: median±IQR; statistics: Kruskal-Wallis test. **I**, Spearman correlation analysis of SP-AS and FP-AS (n=218). FC indicates fold change; and Th, T helper.

To further assess local AV inflammation, we performed gene expression analysis of 594 predefined genes after total RNA extraction of AV tissue (Table S2). Hierarchical cluster analysis of the top 50 differentially expressed genes showed significantly different RNA expression levels in valve tissue derived from patients with FP-AS and SP-AS (Figure 3A through 3C). The most prominently downregulated gene expression signals involved key regulators of the toll-like receptor/inflammasome pathway (eg, TGF-β1), while proinflammatory MIF-dependent pathways were upregulated in FP-AS (Figure 3B through 3D). We describe remarkable differences in the TGF-β1–dependent and MIF-dependent pathways between both patient groups (Figure 3). Pathway enrichment analysis identified the protein translation-related categories complement and coagulation cascade, toll-like receptor signaling, Th17 cell differentiation, and B-cell receptor signaling pathway to be upregulated in FP-AS (Figure 3E and 3F).

To further highlight the importance of MIF-regulated signaling in patients with FP-AS, MIF was added to the network analysis displaying its interactions with significantly enriched gene ontology (Figure 3F) and Kyoto Encyclopedia of Genes and Genomes pathways (Figure S14) This observation is further substantiated by immunohistochemistry of AV tissue showing significantly increased MIF protein expression in FP-AS (Figure 3G). Interestingly, not only the overall MIF expression of valve resident cells of FP-AS patients was enhanced but most prominent MIF expression was detected in cells on the aortal side of the AV cusps (Figure 3G and 3H), while TGF-β1–positive cells were remarkably lower in number in FP-AS (Figure 3G). Furthermore, tissue expression of MIF correlated with tissue cell infiltration of macrophages (CD68^+^), monocytes (CD16^+^), and platelets (CD42b^+^; Figure 3I). Thus, our results imply that MIF and its related pathways are a predominant regulator of local valve tissue inflammation in patients with FP-AS.

### Systemic thromboinflammation in FP-AS Is Characterized by Low Intracellular MIF Expression in Platelets but High MIF Plasma Levels

**Figure 4.**
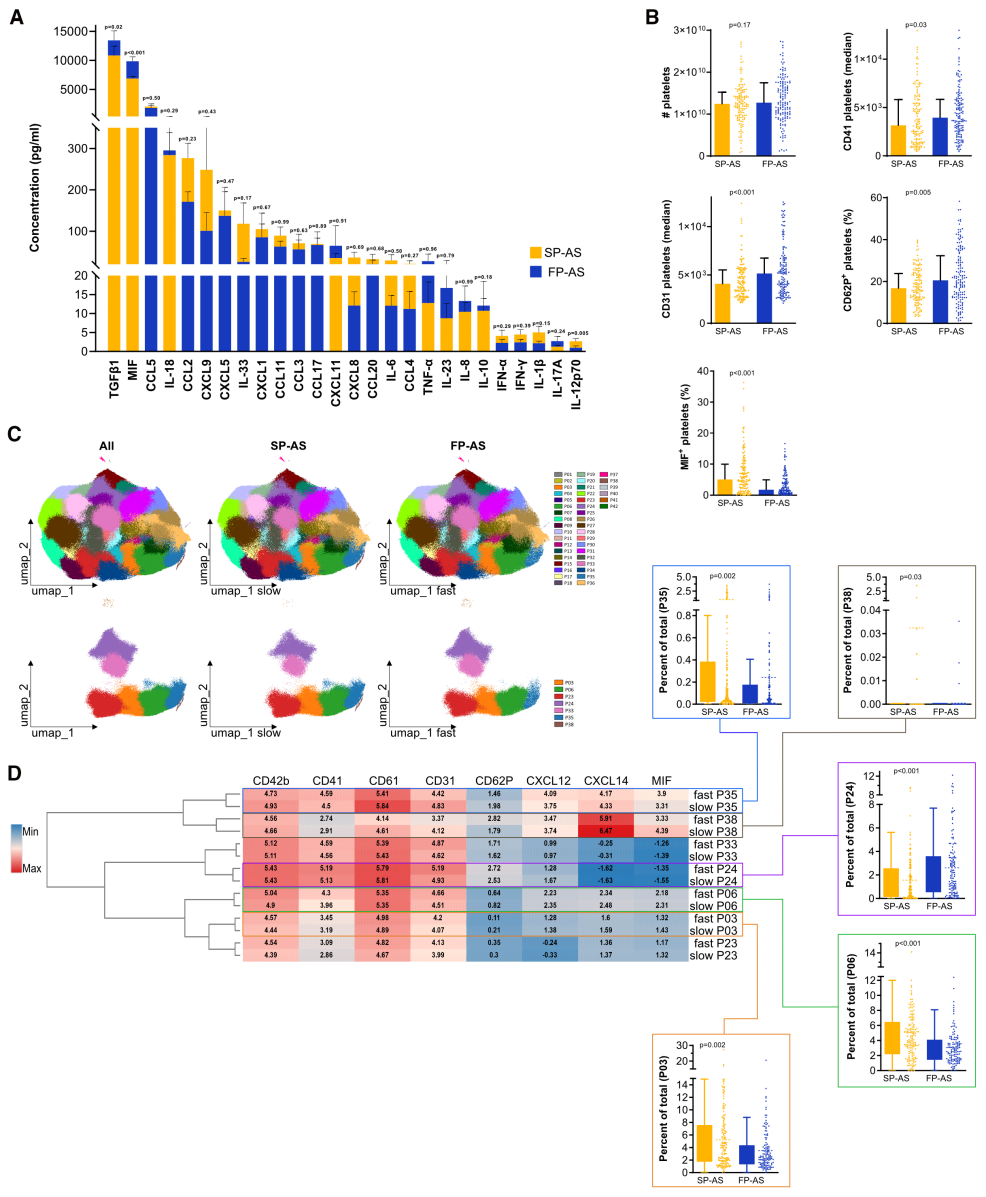
**Immunophenotyping and high-dimensional analysis of platelets in fast-progressive aortic stenosis (FP-AS). A**, Plasmatic inflammatory cytokines/chemokines in slow-progressive aortic stenosis (SP-AS; n=98) and FP-AS (n=95). **B**, Platelets in flow cytometry of SP-AS (n=142) and FP-AS (n=150) showing platelet count, median CD41/CD31, and frequency of CD62P^+^/MIF^+^. **C**, Platelet subpopulations determined by PhenoGraph algorithm for unsupervised clustering of patient samples (n=270). Left plot represents an overlay of platelets from SP-AS and FP-AS followed by individual plots of platelets from SP-AS and FP-AS of all clusters. The lower row shows only significantly different clusters. **D**, Clustered heatmap of significant different clusters P35, P38, P24, P03, and P06 from **C** shows median expression of indicated markers of SP-AS compared with FP-AS. Abundancy of cells in each cluster for each patient is shown as box plots stratified into SP-AS and FP-AS. Plots were generated using the OMIQ data analysis software. MIF indicates macrophage migration inhibitory factor; and umap, uniform manifold approximation and projection.

Next, we asked whether the assessment of systemic thromboinflammation allows to discriminate slow and fast AS progression and whether it is associated with the degree of local valve inflammation. There were no associations between established markers of inflammation-like levels of C-reactive protein (Table S1). Chemokine profiling using an inflammation panel of 26 chemokines/cytokines showed that MIF plasma levels were significantly elevated in FP-AS (MIF; in pg/mL; mean±SEM, 6877±379.1 versus 9959±749.1; *P*<0.001; Figure 4A), whereas other tested mediators, for example, IL (interleukin)-6, IL-1β, or TNF (tumor necrosis factor) showed similar plasma levels in both groups.

Multicolor flow cytometry analysis performing classical manual gating revealed that although the numbers of circulating platelets (Figure S4A) were similar in FP-AS and SP-AS (Figure 4B), their activation status was significantly increased in FP-AS demonstrated by platelet CD62P^+^, CD41, and CD31 expression (CD62P; in percentage; median [IQR], 16.8 [11.58–23.8] versus 20.55 [12.48–32.28]; *P*=0.005), CD41 (3159 [1432–5816] versus 3949 [2567–5843]; *P*=0.03), CD31 (4087 [2918–5515] versus 5147 [3349–6733]; *P*<0.001; Figure 4B). In contrast, intracellular MIF expression in platelets was significantly decreased in FP-AS, indicating an enhanced release reaction of MIF due to increased platelet activation in patients with FP-AS (MIF; in %; 4.85 [1.48–9.75] versus 2.3 [0.78–5.9]; *P*<0.001; Figure 4B).

In addition to this manual gating strategy, we performed unsupervised data analysis by first applying uniform manifold approximation and projection dimension reduction to group phenotypically similar events (Figure S5)^35^ followed by unsupervised clustering analysis using PhenoGraph (Figure 4C).^36^ PhenoGraph analysis resolved 42 clusters (P01–P42), of which 7 showed significant differences between SP-AS and FP-AS (Figure 4C and 4D; Figure S8), confirming the finding obtained by manual gating strategy. Clusters P35, P03, P06, and P38 of platelets expressing either high or even increased amounts of intracellular MIF were more abundant in patients with SP-AS (*P*<0.001; Figure 4D). In contrast, cluster 24 showing increased expression of CD62P and CD31 and less intracellular CXCL12 was more abundant in patients with FP-AS.

**Figure 5.**
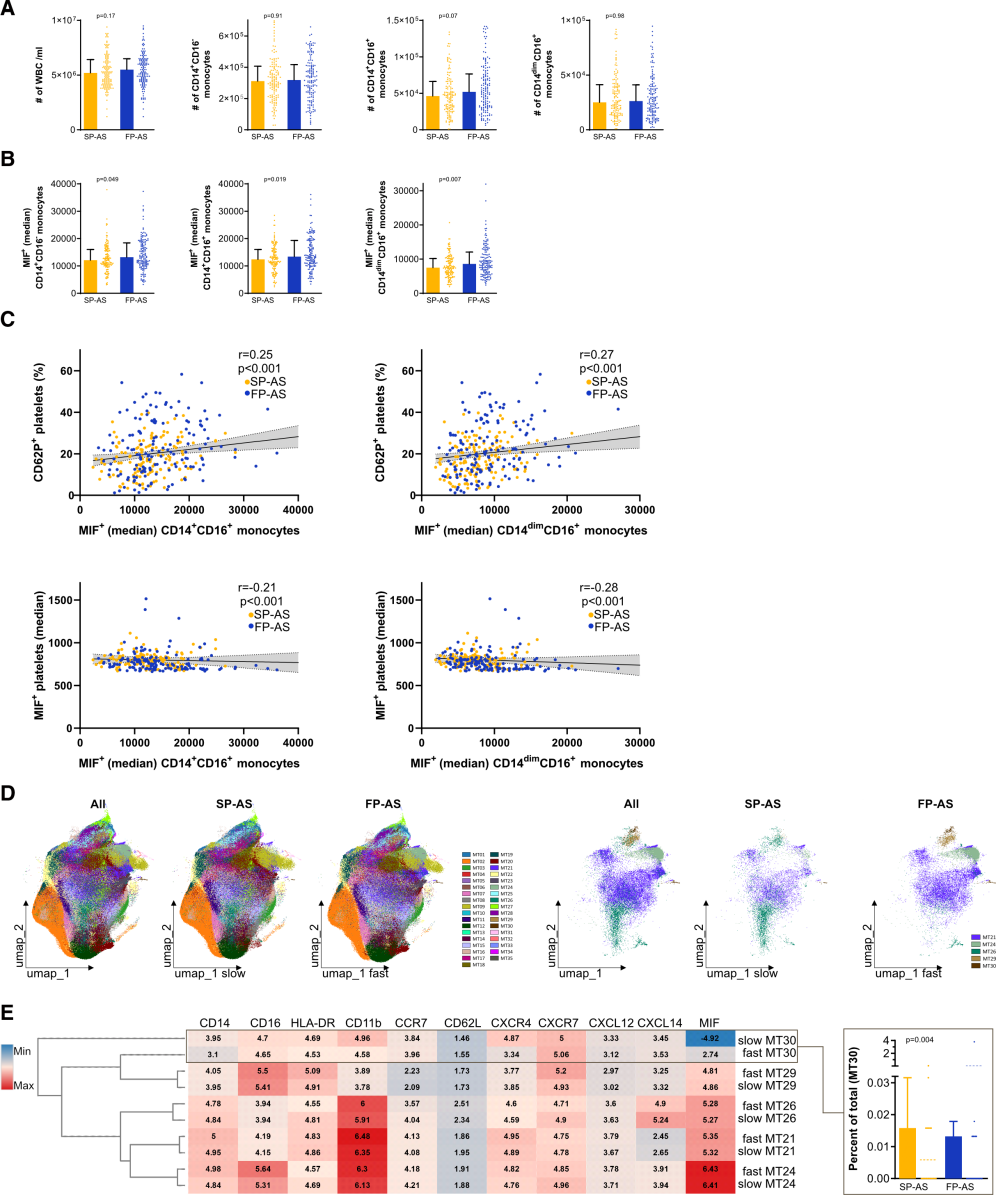
**Expression of MIF (macrophage migration inhibitory factor) in monocyte subsets in fast-progressive aortic stenosis (FP-AS) and slow-progressive aortic stenosis (SP-AS). A**, Flow cytometry of monocytes of SP-AS (n=142) and FP-AS (n=150) to analyze monocyte subsets. **B**, Intracellular MIF expression (median) by monocyte subsets. **C**, Spearman correlation analysis of SP-AS and FP-AS. **D**, Monocyte subpopulation analyzed by PhenoGraph algorithm for unsupervised clustering of patient samples (n=260). Left plot shows overlay of monocytes from SP-AS and FP-AS followed by individual plots of monocytes from SP-AS and FP-AS of all clusters. Lower plots represent only significant different clusters. **E**, Clustered heatmap of significant different clusters from **D** shows median expression of indicated markers for comparison of SP-AS and FP-AS. Abundancy of cells in each cluster for each patient is shown as box plots stratified into SP-AS and FP-AS. Plots were generated using the OMIQ data analysis software. CCR7 indicates C-C chemokine receptor type 7; CXCL, chemokine (C-X-C motif) ligand; CXCR, C-X-C chemokine receptor; HLA-DR, human leukocyte antigen-DR isotype; max, maximum; min, minimum; umap, uniform manifold approximation and projection; and WBC, white blood cell.

As high infiltration of monocytes/macrophages was observed in immunohistochemistry stainings of AVs from FP-AS, we addressed the differences in abundancy and immunophenotype of circulating monocyte subsets in this patient cohort to link local and systemic inflammation regulated by monocytes/macrophages. Similar numbers of white blood cells (median [IQR], 5.2×10^6^ [4.2×10^6^–6.43×10^6^] versus 5.5×10^6^ [4.5×10^6^–6.5×10^6^]; *P*=0.17), as well as circulating classical (CD14^+^CD16^−^, 3.1×10^5^ [2.2×10^5^–4.1×10^5^] versus 3.2×10^5^ [2.3×10^5^–4.2×10^5^]; *P*=0.91), intermediate (CD14^+^CD16^+^, 4.6×10^4^ [2.9×10^4^–6.6×10^4^] versus 5.2×10^4^ [3.1×10^4^–7.7×10^4^]; *P*=0.07), and nonclassical monocytes (CD14^dim^CD16^+^, 2.5×10^4^ [1.5×10^4^–4.1×10^4^] versus 2.6×10^4^ [1.4×10^4^–4.1×10^4^]; *P*=0.98) were observed in both patient groups (Figure 5A; Figure S4). Intracellular MIF expression was the highest in classical and intermediate monocytes, while all monocytes regardless of their subtype showed a high MIF content (Figure 5B). Intracellular MIF expression was increased most prominently in intermediate (MIF; median [IQR], 12 396 [10 076–16 051] versus 13 419 [10 718–19 324]; *P*=0.019) and nonclassical monocytes (SP-AS versus FP-AS; MIF; 7488 [5593–10 185] versus 8601 [6180–12 053]; *P*=0.007) in patients with FP-AS (Figure 5B). MIF expression in monocyte subtypes correlated with the degree of platelet activation indicated by CD62P expression (CD62P^+^platelets/MIF^+^CD14^+^CD16^+^: r=0.25, *P*<0.001; CD62P^+^platelets/MIF^+^CD14^dim^CD16^+^: r=0.27, *P*<0.001) and inversely with MIF expression in platelets (MIF^+^ platelets/MIF^+^CD14^+^CD16^+^: r=0.21, *P*<0.001; MIF^+^platelets/MIF^+^CD14^dim^CD16^+^: r=−0.28, *P*<0.001; Figure 5C). Thus, changes of plasma and intracellular MIF levels in monocytes/platelets are associated with FP-AS.

Unsupervised data analysis using uniform manifold approximation and projection and PhenoGraph algorithms (Figure 5D and 5E; Figures S5 through S7) revealed 35 clusters (MT1–35), of which 5 showed significant differences between SP-AS and FP-AS (Figure 5D). Clustered heatmap analysis of these 5 clusters revealed distinct activation states (CD11b, CD62L, and HLA-DR [human leukocyte antigen-DR isotype]) and expression of MIF, CXCL12, CXCL14, and their receptors CXCR4 and CXCR7 (Figure 5E). The MT30 cluster is significantly reduced in patients with FP-AS showing a clear difference in MIF but also CXCR4 expression (Figure 5E). In summary, we found substantial differences in systemic inflammation markers in monocytes between the 2 groups of AS with prominent changes in MIF-associated markers.

### Plasma and Cell-Associated Levels of MIF Are Significantly Related to Findings of Local AV Tissue Inflammation and Predict Progression of AS

**Figure 6.**
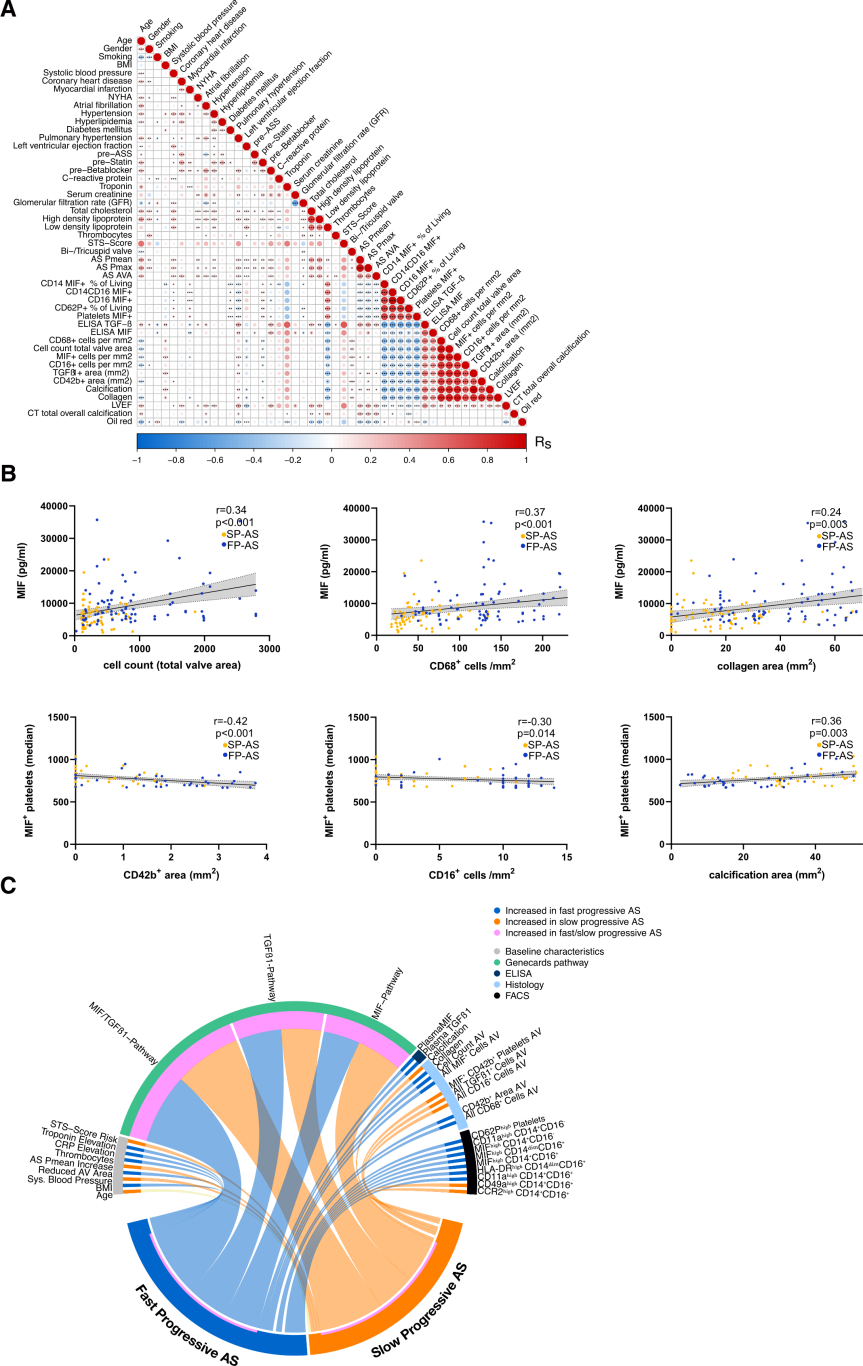
**Plasmatic and platelet-MIF (macrophage migration inhibitory factor) correlate with valvular phenotype in fast-progressive aortic stenosis (FP-AS). A**, Correlation matrix by corrplot R package displays correlations of clinical parameters alongside ex vivo assays. Significant (*P*<0.05) correlations of clinical baseline parameters and important in vitro, ex vivo, and clinical data including flow cytometry, immunohistochemistry, and immunoassay in patients with fast- and slow-progressive aortic stenosis (SP-AS) are illustrated. **B**, Spearman correlation analysis of SP-AS (n=238) and FP-AS (n=237) to evaluate associations with valvular phenotypes characteristic for either SP-AS or FP-AS. **C**, Synopsis of important findings in this study is displayed by chord diagram using R package circlize. Significant (*P*<0.05) changes of clinical parameters and ex vivo data, as well as differentially expressed gene pathways between patients with fast-progressive aortic stenosis (FP-AS; purple) and slow-progressive aortic stenosis (SP-AS; yellow) are illustrated and colored according to the performed assays as indicated in the figure caption. Thus, patients with FP-AS showed a significantly altered risk profile (orange), as well as MIF and TGF (transforming growth factor) expression levels using Nanostring analyses (red), ELISA (purple), immunohistochemical analyses (blue), and flow cytometry (black) analyses.

Further, we hypothesized that changes of plasma and cell-associated MIF levels might reflect the degree of local valvular inflammation. We performed extensive correlation analysis of important clinical factors and thromboinflammation-related parameters (Figure 6A). We found that MIF plasma levels correlate significantly with AV tissue cell infiltration and collagen content (Figure 6B). Platelet-MIF correlates inversely with monocyte and platelet tissue accumulation, while it also correlates positively with calcification area (Figure 6B). Thus, patients with FP-AS are characterized by significant alterations of systemic MIF expression comprising plasma and circulating platelets and monocytes, implying that this cytokine plays a critical role in accelerated valvular inflammation and thereby AS progression. Interestingly, valvular MIF expression and MIF plasma levels were associated with patient age. Younger patients showed more often an inflammatory-driven, fast-progressive phenotype with higher MIF expression in valve tissue and higher MIF plasma levels (Figure S12). However, there were no sex-specific differences among men and women in local and systemic MIF expression (Figure S13). To further assess how varying expression of MIF in platelets and in monocytes might affect their respective functions, we tested monocytes and platelets with high MIF expression regarding their association with markers of activation/adhesion. Here, we found that ^high^MIF^+^ platelets are associated with an increased expression of CD54^+^ in CD14^+^CD16^−^ monocytes as an established adhesion marker. Interestingly, ^high^MIF^+^ CD14^+^CD16^−^ monocytes are associated with an increased number of CD62P^+^ platelets as a marker for platelet activation (Figure S15). Strikingly, patients with FP-AS share elevated MIF levels and MIF-regulated pathways on protein and gene expression levels (Figure 6C). The chord diagram links local protein and gene expression within the AV to systemic MIF- and TGF-β1–related mediators and clinical features pointing out that MIF-regulated pathways are of great importance in FP-AS. Furthermore, in a step-wise linear (Table S3) and in multivariate Cox regression analysis (Table S4), we found that plasma and cell-based MIF markers summarized as liquid biopsy are strongly associated with FP-AS, independent of cardiovascular comorbidities and functional parameters. Liquid biopsy remained significantly associated with FP-AS in all models shown and an independent predictor of FP-AS among all risk factors tested (Table S3). Additionally, we also tested for possible confounding effects of cardiovascular risk factors (diabetes type 2, smoking status, arterial hypertension, and hyperlipidemia), comedication (P2Y12 [purinergic receptor type Y, subtype 12 inhibitors]), and comorbidities (heart failure defined by New York Heart Association class, symptomatic coronary artery disease, chronic kidney disease, and atrial fibrillation) on the progression rate of AS using PLS-DA and OPLS-DA analysis depicted in Figure S11. Here, we confirmed that none of the tested clinical parameters showed a significant influence on the progression rate of AS (Figure S11). Furthermore, we confirmed that valvular MIF expression and MIF plasma levels were associated with patient age but did not show any sex-specific differences among men and women (Figures S12 and S13).

## DISCUSSION

The underlying pathophysiological mechanisms of AS are only incompletely understood.^2,9^ We hypothesized that changes in platelet activity and platelet-derived mediators of systemic thromboinflammation are of prognostic relevance for an accelerated course of the AV disease.

Here, we present data showing that (1) patients with fast disease progression (FP-AS) reveal an inflammatory phenotype of the affected AV, which significantly differs from a calcifying valve pathology in SP-AS. (2) MIF-related pathways are predominantly upregulated in valvular tissue of patients with FP-AS. (3) Platelet activity along with plasma and cell-based markers of systemic inflammation is associated with local valvular inflammation and allows to discriminate an accelerated inflammatory from a calcifying valvular phenotype. (4) Peripheral MIF-based biomarkers predict FP-AS and are independent from demographic and clinical confounders. Valvular MIF expression and MIF plasma levels were associated with patient age but did not show any sex-specific differences among men and women indicating that MIF serves as a robust predictive biomarker in all patients. To date, a watch-and-wait strategy is generally performed in all patients with AS irrespective of disease progression rate.^8,9,11^ Therefore, the development of an effective prevention strategy of AS is an unmet need in modern patient care.^13^ Our findings imply that targeting MIF secretion and MIF-regulated processes pharmacologically may be a future treatment option to delay/prevent progression of DAVD.

Our findings extent the current knowledge^18^ as we demonstrate that local platelet infiltration and accumulation is associated with an inflammatory valvular phenotype that shows enhanced numbers of infiltrating monocytes/macrophages. This suggests that platelets and immune cells, both, might influence the differentiation of VICs resulting in fibrosis and calcification.^1,13^ Platelet-triggered local inflammation might be another key component in the complex mechanisms of DAVD. We suggest an important impact of platelet activation and secretion not only locally but also in systemic thromboinflammation during DAVD. Platelets have been well recognized to play a critical role in inflammation and atherogenesis^17,24,26^ but also seem to regulate important processes in AS. Our findings are in line with other studies that describe a platelet-mediated osteogenic differentiation of AS^17^ and add a deeper insight in platelet-regulated processes along with its associations with disease progression. Inhibition of platelet adhesion attenuates vascular inflammation and plaque formation^26^ in humans and in animal models, for example, atherosclerotic lesion formation of aortic sinus/cusps in ApoE^−/−^ mice^26^ was inhibited by platelet inhibition, suggesting an important role in local inflammation.

Platelets and monocytes store and release cytokines and chemokines that orchestrate immune cell trafficking to the inflamed tissue.^37,38^ Platelet activation might also occur in AS as a result of shear stress originating from turbulent flow pattern of the AV.^13^ Platelets are a major source of MIF and other proinflammatory cytokines/chemokines that are released upon activation.^21,39^ Here, platelets secrete a variety of inflammatory mediators including MIF that boost vascular inflammation.^40–42^ MIF also plays a pivotal role in atherogenesis as deficiency of MIF reduces atheroprogression in LDLR^−/−^ (low-density lipoprotein receptor) mice.^43^ Most interestingly, we found that MIF expression by circulating platelets is reduced in FP-AS, while MIF plasma levels are enhanced, as well as MIF expression in circulating monocytes. MIF, a proinflammatory immunomodulator that has broad effects on the inflammatory response and the immune system signals via CXCR2/4/7 and CD74, initiates inflammatory cell recruitment and proinflammatory gene expression.^39^ Our study shows that MIF might be of great importance in the regulatory processes of local and systemic thromboinflammation during AS. Our present findings imply that circulating platelets in FP-AS degranulate, release MIF, and interact with circulating monocytes. Further studies are needed to dissect the direct interaction of platelets with the various monocyte subsets, for example, by using a more advanced flow cytometry panel addressing platelet/monocyte interactions.

We also identified MIF plasma levels released from activated platelets as a critical prognostic factor for accelerated AS. Thus, inhibition of platelet degranulation or direct inhibition of MIF through antagonists may be a possible pharmacological strategy to modulate accelerated local valve inflammation and thereby progression of AS. MIF antagonists have been developed preclinically, inhibit MIF-related monocyte function, and attenuate atherosclerosis.^26,39,43^

Extracellular MIF independently initiates downstream MAPK (mitogen-activated protein kinase) or PI3K (phosphoinositide 3-kinase) pathway effectors. In detail, MIF-induced signaling acts through relatively few downstream pathways, which frequently converge on the MAPK, PI3K/AKT (phosphoinositide 3-kinase/serine/threonine kinase), NF-κB (nuclear factor-κB), ERK (extracellular signal-regulated kinase) signaling, and p53-mediated apoptosis and growth arrest, which all might play an important role for chronic inflammatory processes and in the regulation of innate immune cells. The main signaling pathway is MIF-mediated glucocorticoid regulation, which comprises STAT3 (signal transducer and activator of transcription 3), IL-6, endothelin-1, and PGC1α (peroxisome proliferator-activated receptor gamma coactivator 1-alpha) signaling.^44^

In our patient cohort, we found significant differences in the gene expression of MIF-mediated signaling pathways between FP-AS and SP-AS in Nanostring analysis. Among the top 50 differentially expressed genes, we also identified several genes that have been well described to be MIF related and to drive inflammatory processes. Among others, we found that genes that play a key role in the MIF-mediated glucocorticoid regulation, which is of great importance in inflammation, were significantly upregulated in FP-AS. Here, we illustrate that CX3CL1 (chemokine (C-X3-C motif) ligand 1), TNFSF8 (tumor necrosis factor superfamily member 8), and TNFSF13B are increased in FP-AS, while we found enhanced expression of NF-κB, IL17B, and JAK2 in SP-AS. Among others, TNFSF13B has been described as a cytokine that is primarily produced by monocytes and neutrophils and plays a crucial role in B-cell homeostasis. Our findings suggest that MIF-mediated TNFSF13B expression may contribute to inflammatory processes in FP-AS. Enhanced TNFSF13B in FP-AS is an interesting finding as it can be addressed therapeutically by the monoclonal antibody belimumab.^45,46^ Furthermore, our Nanostring findings also show different expression of regulatory genes that play an important role in the regulation of the innate immunity and the complement cascade, like SERPING1 (serine proteinase inhibitor family G member 1), which regulates complement activation and innate immunity like MIF. Its expression is significantly increased in FP-AS. Finally, MIF and CX3CL1 (fractalkine), both are expressed in immune cells, platelets, and endothelial cells and participate in monocyte/macrophage recruitment to the site of injury. In our cohort, we found that CX3CL1 expression is significantly increased in FP-AS indicating again a pronounced inflammatory response in the valvular tissue of this phenotype. As CX3CL1 shows synergistic effects with MIF, we can argue that both signaling pathways regulate platelet and monocyte/macrophage activation and thereby inflammation of the valve tissue.

Interestingly, we also found different gene expression of NF-κB, which is essential for inflammation and immunity and regulates the expression of numerous chemokines, cytokines, transcription factors, and regulatory proteins. Here, we show a significant upregulation in SP-AS, which might indicate that NF-κB activation plays an important role in inflammatory and later on calcifying processes. As functional NF-κB–binding sites exist within the MIF promoter, it has been described that oxLDL (oxidized low-density lipoprotein)-mediated MIF induction is NF-κB dependent in atherosclerosis and calcifying processes. Our findings are in line with previous studies on atherosclerotic plaque formation and atheroprogression. It is tempting to speculate that MIF-mediated and NF-κB–mediated signaling might also lead to first rapid and afterward chronic inflammatory processes, which results in osteogenic differentiation and rather slower calcification of the valvular tissue.^47,48^

Our findings in Nanostring analysis support our hypothesis that the MIF-regulated inflammation is enhanced in the inflammatory valvular phenotype of FP-AS.

Specific platelet and monocyte phenotypes in addition to MIF-related biomarkers can not only help to identify patients with DAVD that are at risk for fast progression but may also be addressed by novel anti-inflammatory therapeutic strategies in the future. Therefore, anti-inflammatory strategies should be further investigated in larger clinical studies. At present, several preclinical and clinical compounds are available to modulate the function of MIF in vitro and in vivo.^40–42,49^ For example, the PDE4 (phosphodiesterase-4) inhibitor ibudilast has potent anti-inflammatory activity and inhibits platelet aggregation.^41^

In conclusion, we identified MIF plasma levels released from activated platelets as a critical prognostic factor for patients at risk for FP-AS. Targeting release of platelet-derived MIF may be a potential strategy to decelerate inflammatory, fibrocalcific, and osteogenic processes in AS.

## ARTICLE INFORMATION

### Acknowledgments

K.A.L. Mueller, M.P. Gawaz, and S.E. Autenrieth contributed to the conception and design of the study, interpretation of data, drafting of the manuscript, and revising it critically for important intellectual content; C. Langnau, T. Harm, M. Sigle, M. Droppa, O. Borst, A.-K. Rohlfing, S. Gekeler, M. Günter, H. Janning, D. Rath, and K.-P. Kreisselmeier performed in vitro experiments, experimental analysis, flow cytometry, data acquisition, and interpretation; K. Mott, H. Schulze, S. Scheuermann, and C.M. Seitz performed additional valve analysis and colocalization experiments for revisions and helped to revise the manuscript. T. Castor and I.I. Mueller performed experimental analysis; N. Goebel, U.F.W. Franke, M. Radwan, and C. Schlensak organized sample collection, data acquisition, and interpretation; M. Droppa performed computed tomography data analysis and interpretation; M. Sigle, T. Harm, and K.-P. Kreisselmeier contributed to the interpretation of data and revising manuscript critically for important intellectual content; T. Castor and D. Rath contributed to the interpretation of data and revising manuscript critically for important intellectual content.

### Sources of Funding

This work was supported by the German Research Foundation (DFG), project number 374031971–TRR 240, by the Ministry of Science, Research and the Arts of the State of Baden-Württemberg (COVID-19 Funding), and by German Foundation for Heart Research (DSHF, F/45/22).

### Disclosures

None.

### Supplemental Material

Expanded Materials & Methods

Tables S1–S4

Figures S1–S15

Major Resources Table

## Supplementary Material
