# Supplementary material for: Macrophage Migration Inhibitory Factor Promotes Thromboinflammation and Predicts Fast Progression of Aortic Stenosis
Source: Arterioscler Thromb Vasc Biol. 2024 Jul 11;44(9):2118–35. doi: 10.1161/ATVBAHA.124.321000 (PMC11335082; doi:10.1161/ATVBAHA.124.321000)
Supplement: Supplementary file 1 [file atv-44-2118-s001.pdf]

Supplemental Materials

Macrophage migration inhibitory factor promotes thrombo-inflammation and predicts fast progression of aortic stenosis

Karin Anne Lydia Mueller[1*], MD, Carolin Langnau[1], PhD, Tobias Harm[1], MD, Manuel Sigle[1], Kristina Mott[2], PhD, Michal Droppa[1], MD, Oliver Borst[1,3], MD, Anne-Katrin Rohlfing[1], PhD, Sarah Gekeler[1], Manina Günter[4,5], Nora Goebel[6], MD, Ulrich F.W. Franke[6], MD, Medhat Radwan[7], MD, Christian Schlensak[7], MD, Henrik Janning[1], Sophia Scheuermann[8,9], Christian M. Seitz[8,9], MD, Dominik Rath[1], MD, Klaus-Peter Kreisselmeier[1], MD, Tatsiana Castor[1], PhD, Iris Irmgard Mueller[1], MD, Harald Schulze[2], PhD, Stella E. Autenrieth[4,5#], PhD, Meinrad Paul Gawaz[1#], MD.

[1]University Hospital Tuebingen, Department of Cardiology and Angiology, Eberhard Karls University Tuebingen, Tuebingen, Germany

[2]Institute for Experimental Biomedicine, Chair I University Hospital Würzburg Josef-Schneider-Str. 2, 97080 Würzburg, Germany

[3]DFG Heisenberg Group Thrombocardiology, University of Tübingen, Tübingen, Germany

[4]University Hospital Tuebingen, Department of Hematology, Oncology, Clinical Immunology and Rheumatology, Eberhard Karls University Tuebingen, Tuebingen, Germany

[5]Dendritic Cells in Infection and Cancer, German Cancer Research Center (DKFZ), Heidelberg, Germany

[6]Robert-Bosch Hospital, Department of Cardiovascular Surgery, Stuttgart, Germany

[7]University Hospital Tuebingen, Department of Thoracic and Cardiovascular Surgery, Eberhard Karls University Tuebingen, Tuebingen, Germany

[8]Department of Pediatric Hematology and Oncology, University Children's Hospital Tuebingen, 72076, Tuebingen, Germany

[9]Cluster of Excellence iFIT (EXC 2180) "Image-Guided and Functionally Instructed Tumor Therapies", University of Tuebingen, 72076, Tuebingen, Germany

Short title: MIF in fast progressive aortic stenosis

[#]contributed equally

[*]Corresponding author:
Karin Anne Lydia Mueller, MD
Department of Cardiology and Angiology
University Hospital of the Eberhard Karls University Tuebingen
Otfried-Mueller-Str.10, 72076 Tuebingen, Germany
Tel: +49-7071-29-83688
Fax: +49-7071-29-5749
E-mail: k.mueller@med.uni-tuebingen.de

Materials and Methods

Because of the sensitive nature of the data collected for this study, requests to access the dataset from qualified researchers trained in human subject confidentiality protocols may be sent to corresponding author. This study complies with the Declaration of Helsinki, was approved by the local ethics committee Tuebingen, Germany (proposal number 240/2018B02). The study complies with good clinical practice guidelines on the approximation of the laws, regulations, and administrative provisions of the member states relating to the implementation of good clinical practice in the conduct of clinical trials on medicinal products for human use. Written informed consent was obtained from every patient. Because of the sensitive nature of the data collected for this study, requests to access the dataset from qualified researchers trained in human subject confidentiality protocols may be sent to Karin Mueller at University Hospital Tuebingen, Department of Cardiology, and Angiology, Eberhard Karls University Tuebingen, Germany.

Study design, patient cohort and inclusion criteria

From December 2014 through March 2021, we prospectively enrolled 475 consecutive patients with severe symptomatic (NYHA class ≥ 2) aortic stenosis (AS) with indication for aortic valve (AV) repair who presented at the Department of Cardiology and Angiology or at the Department of Heart Surgery of the University Hospital Tübingen, Germany. All patients underwent clinical and cardiac examination including repetitive echocardiography, electrocardiography, assessment of medical history at baseline, concomitant medication, comorbidities, and blood sampling for routine laboratory parameters, marker expression on platelets and monocytes, as well as chemokine profiling. Tissue samples of explanted AVs were obtained from all patients who underwent surgical valve replacement and were analyzed by histology, immunohistochemistry, and gene expression analysis using Nanostring technology. Clinical risk assessments were based on the scoring risk model developed by the Society of Thoracic Surgeons (STS)[8, 9] using an algorithm that is based on the presence of

coexisting illnesses to predict mortality at 30 days expressed as a percentage. Coronary artery disease (CAD) was determined by coronary angiography, and was characterized as > 25-50% stenosis of one or more coronary vessels.

Inclusion criteria of our study were age older than 18 years and presence of severe symptomatic aortic stenosis determined in echocardiography. Furthermore, the availability of repeated echocardiography data before surgery was obligatory to calculate the change of the peak velocity over the AV (ΔVmax). Exclusion criteria comprised any present microbial systemic infection, suspected or active bacterial endocarditis within 180 days of study inclusion, history of severe liver cirrhosis or any active liver disease, and significant frailty as determined by the Heart Team with life expectancy < 1 year.

As laboratory parameters we determined leucocyte and platelet count, N-terminal-pro-B-type natriuretic peptide (NT-pro-BNP, > 300 ng/L), high-sensitive troponin I (hs TNI, > 37 ng/L), and C-reactive protein (CRP, > 0.5 mg/dL) among others as established markers of myocardial and inflammatory distress.

Echocardiographic Parameters

Echocardiographic assessment and standard measurements were performed in all patients according to the European Association of Echocardiography/American Society of Echocardiography (EAE/ASE) guidelines.[8, 9] LV ejection fraction was measured using the biplane Simpson method in the apical 4- and 2-chamber views. The diagnosis and severity of AS was classified according to European Association of Echocardiography/American Society of Echocardiography guideline (EAE/ASE) recommendations.[8, 9] We measured the transvalvular mean (Pmean) and peak pressure gradient (Pmax) of the AV and the aortic valve area (AVA) accordingly. The outer edge of the AS jet velocity curve in continuous-wave Doppler imaging was traced to obtain the peak jet velocity of the AS [Vmax (m/s)] along with [Pmax/Pmean (mmHg)]. The AVA was calculated using the continuity equation. The AV phenotype was recorded as bicuspid or tricuspid. Degenerative aortic valve disease (DAVD)

and severity of AS was defined by hemodynamic parameters in accordance to the current guidelines.[8, 9] Stroke volume and stroke volume index as well as the left ventricular ejection fraction were obtained according to current guidelines.[8, 9]

Rate of AS progression

In our consecutive cohort of patients with severe AS we identified two subgroups of patients with symptomatic AS regarding fast (FP-AS) and slow disease progression (SP-AS) by repetitive transthoracic echocardiographic assessment in a retrospective analysis. In repeated transthoracic echocardiography before surgery we determined the rate of disease progression by the change of the maximum transvalvular jet velocity (ΔVmax) over time and established an annualized progression rate ΔVmax (m/s/year) as described before.[2, 4, 5, 8 ,9] The median annualized ΔVmax was used as cut-off and thereby two subgroups of patients were evident with either FP-AS (ΔVmax ≥ 0.45 m/s/year) or SP-AS (ΔVmax < 0.45 m/s/year) until onset of severe symptoms occurred that warranted surgical valve replacement (**supplemental figure S1**).[5] The following formula was used to determine the annualized ΔVmax:[2, 4]

Annualized ΔVmax (m/s/year) = (final Vmax − first Vmax) / Δ time,

where 'Final Vmax' is the maximum jet velocity immediately prior to the surgery, 'first Vmax' is the maximum jet velocity from the oldest available echocardiography report and/or the echocardiography closest to 12 months prior to valve replacement, and Δ time is the time elapsed (in years) between the latest and first echocardiography reports.[2, 4-7] All patients underwent repetitive, at least 2 transthoracic echocardiograms, to evaluate AS progression. To discriminate between fast and slow progressive AS, we defined a calculated annualized ΔVmax of 0.45 m/sec/year (defined as median ΔVmax in the overall patient cohort: n=237 patients with fast progressive AS and a ΔVmax ≥ 0.45 m/sec/year; n=238 patients with slow progressive AS and a ΔVmax < 0.45 m/sec/year.The time gap between the final pre-surgery and the first available echocardiography reports and/or the echocardiography report closest to 12 months prior to surgery, ranged from 1.3 months to 23.0 months. The average duration

from first echocardiography to symptomatic severe AS with indication for valve replacement was 39.0 ± 39.2 months, median duration was 43.2 months (IQR 11,7 - 50.9) for the overall cohort. In patients with fast progressive AS the duration was shorter with an average of 12.4 ± 7.8 months; median 10.8 months (IQR 6.7-17.6) compared to slow progressive disease with a mean duration of 60.3 ± 41.3 months, median 48.6 months (40-63.5), p<0.001. The number of echocardiographies varied between 2 to 18 transthoracic echocardiographic exams in the observed cohort during this time interval.

Computed tomography scan of the aortic valve and the aorta

In a subgroup of n=134 patients computed tomography scan of the aortic valve and the aorta was performed before valve replacement. The 3mensio Valves software (PIE Medical Imaging, Maastricht, The Netherlands) was used for the analysis. First annulus plane was defined according to cusps insertion points. For the purposes of quantifying the degree of valvular calcification, the aortic root was separated into two regions along its double-oblique long axis: the leaflet area extended from annulus plane to the lower coronary artery and the left ventricular outflow tract (LVOT) from the annulus plane to 10 mm immediately below it. These regions were further divided into three distinct sectors across the annular plane, corresponding to the non-coronary (NC), left coronary (LC) and right coronary (RC) cusps (C). Calcification (mm^3) was measured with predefined 500-HU threshold with manual adjustment if necessary.[8, 9] This method was chosen over the standard Agatston method due to increased reproducibility.

Procurement of aortic valve tissues for analysis

AV cusps were obtained from all patients undergoing AV replacement surgery at the University Hospital Tuebingen between December 2014 and March 2021.

All valves underwent pathological assessment after removal and if endocarditis was suspected the valves were excluded for the present analysis. The indication for surgery of our patient population was severe symptomatic aortic valve stenosis.

After surgical removal, the AV cusps were immediately placed in an ice-cold solution containing sodium chloride, were stored in 4% paraformaldehyde at 4 °C or RNAlater (ThermoFisher Scientific, Waltham, Massachusetts, USA) or Tissue-TeK® (Sakura Finetek, Alphen aan den Riijin, Netherlands) and stored at -80 °C until analysis. The average dwell time upon excision to valve procurement was kept within 30 min.

Histological and immunohistochemical analysis of aortic valve cusps

Although all patients had clinical diagnosis of severe AS, there was pathological variability in the extent of involvement of aortic valve cusps.

Measurements of gross calcification

Standardized overview pictures of all aortic valves were obtained under similar recording conditions: valve fragment on a black background, same exposure (standard adjustment of front camera of Samsung Galaxy A70), same angle (90 °), and same distance (approx. 60 mm) to the leaflet. The percentage of positive area of calcified nodules was calculated with the software NIS-Elements AR Version 5.21.00 64 bits. All AVs had portions of the attachment of the aortic root, and the tip of the valve, allowing to calculate the uncalcified distance measurements for conclusions about disease progression. The thickness of the valve was measured by a caliper in mm. The number of pixels were converted to mm within the software followed by measurement, and calculation of the total area, calcified area, the diameter, and the distance from the outer edge to the calcified area.

Histology

Formalin-fixed, paraffin-embedded aortic tissue samples were serially sectioned at 6 µm thickness with a Mikrotom (Leica, Jung Multicut). AV cusps were sectioned vertically for histological and immunohistochemical analysis. Paraffin-embedded specimens were stained to illustrate the layered architectural pattern and to determine calcific deposition of the chosen cusp areas. For morphological assessment of the aortic valve, sections were labeled with the following staining's according to the manufacturer's instructions: hematoxylin eosin, von Kossa (DIAPATH-Weinkauf Medizintechnik) for visualization of calcium, and Movat pentachrome (DIAPATH-Weinkauf Medizintechnik) for visualization of collagen.

Immunohistochemistry

After deparaffinization by descending alcohol series (Roti®Histol, 100% until 70% alcohol), the tissues were boiled in citrate buffer (pH 6) 3 times for 5 min in a microwave. After cooling of the tissues for about 15 min they were washed 3 times for 5 min with PBS (Thermo Fisher Scientific, Waltham, Massachusetts, USA). Tissues were incubated for 15 min in 3% hydroxide peroxide (Carl ROTH, Karlsruhe, Germany) followed by another washing step with PBS. Tissues were blocked with serum-free block solution (Agilent, Santa Clara, California, USA) using 2 drops of the ready-to-use solution, and incubated for 30 min at room temperature. 30 µl of primary antibodies, and the corresponding IgG control were applied and diluted in PBS. For negative control primary antibodies were replaced by PBS. Negative control staining for unspecific binding was performed for primary antibodies with appropriate immunoglobulin G control antibodies. Tissue was incubated overnight (14 hours) at 4 °C in a humid chamber. Primary mouse anti-human CD68 (Santa Cruz, Dallas, Texas, USA), CD16 (Santa Cruz, Dallas, Texas, USA), CD42b (Santa Cruz, Dallas, Texas, USA), and MIF antibodies (abcam, Cambridge, England) were used. Primary rabbit anti-human antibodies were used for TGFß-1 (Santa Cruz), and BMP2 (BIOZOL Diagnostics, Eching, Germany). Staining of human thrombus served as a positive control for CD42b, CD68 and CD16 staining, while TGFß-1 and

BMP2 were evaluated in human myocardium. After a washing step with 0.05% Tween20 (SIMGA ALDRICH, St. Louis Missouri, USA) in PBS, incubation with anti-mouse/rabbit biotinylated secondary antibodies (Agilent, Santa Clara, California, USA) was performed. Incubation was performed for 10 min or up to 1 hour in a dark chamber at RT followed by a washing step with 0.05% Tween/PBS. 1-2 drops of Dako´s Streptavidin-HRP-conjugate (Agilent, Santa Clara, California, USA) was added for 10 min. After the last washing step with 0.05% Tween/ PBS, the tissue was incubated with the DAB/LSAB kit (Agilent, Santa Clara, California, USA). Further information for all antibodies and other major resources can be found in **supplemental Table I**. Incubation duration was stopped after macroscopic assessment of the section, as soon as these had to been turned brown. Finally, the tissues were counterstained with hemalaun (Merck, Darmstadt, Germany) for 3 min. The tissues were blued for 15 min under cold running water. Afterwards the ascending alcohol series was performed, and the sections were finally mounted in Roti®-Histokit (Carl ROTH, Karlsruhe, Germany), and cover slipped. An established scoring system was adopted for visual interpretation of the serial slices to allow semi-quantitative analysis of the data. The number of positive cells per millimeter square or the area of positive section per whole valve area was determined for the overall valve tissue, for the lamina fibrosa (aortal side), the lamina spongiosa (interstitial layer) and the lamina ventricularis (ventricular side of the cusps) where applicable. According to the manufacturer's data sheet collagen and reticular fibers are stained yellow within the tissue by Movat pentachrome staining. Exact percentages and total numbers of collagen positive areas were calculated from Movat pentachrome staining. Collagen area was quantified by positively stained area percentage and absolute area normalized to whole valve tissue area. The area was determined using a custom color deconvolution plugin for ImageJ (**supplemental figure S2**). Colors for positively stained, negatively stained and background were selected using a color picker. The RGB values were then defined in the script and all images were batch-analyzed using the same thresholds and color values. If areas were misidentified in the first run, thresholds or color values were adapted and the script reapplied to all images. All images were manually controlled by two experienced investigators. The sum of the yellow stained area

and thereby the collagen area was calculated in either mm^2 or exact percentages of positively stained areas were determined, where applicable. For that, the exact same region of the valve and similar in size valve tissue sections were used. The procedure was automated using a custom batch-script and the same color and threshold values for all individuals.

Microscopy

Aortic tissue sections were visualized with the Nikon Eclipse Ni microscope, and images were captured with the Nikon camera. Briefly, formalin-fixed aortic valves with varying degrees of macroscopic disease were analyzed for the presence of calcification areas. Analyses were undertaken in a blinded manner by 2 primary examiners, and results were cross-referenced by a trained cardiovascular pathologist.

Immunofluorescence microscopy of aortic valves

Aortic valves were fixed in 4 % PFA/PBS solution at 4 °C and subsequently transferred to a serial sucrose gradient of 10, 20 and 30 % (w/v) for 24 h at 4 °C each. Aortic valves were then embedded in SCEM cryomatrix, frozen at – 80 °C and sliced with a Leica CM1900 cryotom into 7 µm sections by using Kawamoto adhesive film. For immunofluorescence staining sections were rehydrated in PBS and blocked with blocking buffer containing 5 % fetal calf serum (FCS), 0.1 % Tween-20 and 10 % rat serum. Sections were incubated with the respective antibodies (CD42b-FITC, clone HPI1; CD14-FITC, clone M5E2; CD16-FITC, clone 3G8; CD68-FITC, clone Y1/82A; TGFβ1-Alexa647, clone 1018746 and MIF-Alexa594, clone 932606) for 45 min at room temperature. Samples were mounted with DAPI-containing Fluorshield (Sigma-Aldrich) to counterstain nuclei. Image acquisition was done using a TCS SP8 confocal laser scanning microscope (Leica Microsystems CMS) with a 40x/NA 1.3 oil objective and LAS X software. Images were processed with Image J (Version 2.9.0/1.53t, NIH).

Gene expression analysis by NanoString® Technology

Ribonucleic acid (RNA) profiling technology (**supplemental table S2**) expression was evaluated using the nCounter Human Immunology v2 Panel (NanoString Technologies, Inc., Seattle, Washington, USA) comprising 594 pre-defined genes. Total RNA was extracted from six sections of formalin-fixed paraffin-embedded tissue sections using the RNeasy FFPE Kit (Qiagen, Hilden, Germany) according to the manufacturer's instructions. RNA yield and purity were assessed using the NanoDrop ND-1000 spectrophotometer (NanoDrop Technologies, Rockland, USA). mRNA expression was measured with the NanoString nCounter Analysis System (NanoString Technologies, Seattle, USA) using 100 ng of total RNA. The nCounter Human Immunology v2 Panel was performed and hybridized to total RNA for 18 h at 65 °C and nCounter Prep Station loading as well as expression quantification with the nCounter Digital Analyzer was performed as recommended by the manufacturer. The expression data were analyzed utilizing the NanoString nSolver Analysis Software v3.0. Quality control of the data was performed using the default settings within the nSolver software and by analyzing the positive and negative control, housekeeper and total (excluding controls) counts as well as the binding densities in each sample. All samples depicted in this study passed the quality control conditions, none were excluded from the study. Agglomerative Cluster - Heat Map analysis using the Pearson Correlation distance measure was performed with the nSolver software according to the manufacturer's instructions. A complete list of genes is supplied in **supplemental table S2** below.

Flow Cytometry staining

Blood samples of all patients with symptomatic severe AS and indication for valve replacement were collected in a citrate-phosphate-dextrose-adenine (CPDA) monovette for phenotyping of monocyte subsets and platelets by flow cytometry staining. Platelets were analyzed in whole blood by diluting 5 µl 1:10 in HEPES-Tyrode's buffer (pH 7.4) in FACS tubes. For staining of monocyte subsets, whole blood was lysed by 10 x RBC Lysis buffer (BioLegend, San Diego,

California, USA) diluted 1:10 in Millipore water. Two washing steps with PBS containing 1% FCS, 2 mM EDTA, and 1% sodium azide were performed followed by adjustment of the cell count to $3x10^6$ cells per tube. The Fc part of both, monocytes and platelets were blocked for 20 min at 4 °C by using human IgG (0.01 mg/ml; Sigma Aldrich Co. St. Luis, Missouri, USA) to avoid unspecific binding of the antibodies.

First, monocytes were extracellularly stained using fluorochrome-conjugated antibodies: CCR2 BV605 (clone K036C2) from BioLegend, San Diego, California, USA for 1 h at 37 °C followed by an extracellular staining with HLA-DR PerCP-Vio77 (clone REA805 Miltenyi, Bergisch Gladbach, Germany), CD3 BV510 (clone OKT3), CD15 BV510 (clone W603), CD19 BV510 (clone HLB19), CD20 BV510 (clone 2H7), CD56 BV510 (clone HCD56), CD14 FITC (clone M5E2), CD16 BV711 (clone 3G8), CD40 BV421 (clone 5C3), CD11b BV605 (clone ICRF44), CD11a BV650 (clone HI111), CD49a PE-Cy7 (clone TS2/7), Zombie NIR from BioLegend (San Diego, California, USA), and CXCR4 BV650 (clone 12G5) as wells as CXCR7 BV421 (clone 10D1) from BD Biosciences (Franklin Lakes, New Jersey, USA) for 20 min at 4 °C. Platelets were stained in the same way for 20 min at 4 °C using following fluorochrome-conjugated antibodies: CD41 PaBl (clone HIP8), CD42b PerCP-Cy5.5 (clone HIP1), CD62P PE-Cy7 (clone AK4), CD61 FITC (clone VI-PL2), and CD31 BV711 (clone WM59) from BioLegend (San Diego, California, USA). For Life/Dead staining Zombie NIR (BioLegend, San Diego, California, USA) was used and performed together with the extracellular antibody staining in one master mix tube. PBS containing 1% FCS, 2 mM EDTA, and 1% sodium azide was used as buffer. Cells were fixed overnight at 4 °C with FoxP3/ Transcription Factor Staining Buffer Set containing formaldehyde (Thermo Fisher Scientific, Waltham, Massachusetts, USA) according to manufacturer's instructions. For permeabilization of the cells, 10x Perm buffer (Thermo Fisher Scientific, Waltham, Massachusetts, USA) was used followed by an intracellular staining of both, monocytes and platelets using fluorochrome-conjugated antibodies CXCL12 PE (clone 79018), CXCL14 AF647 (clone MM0213-12B24), and MIF AF594 (clone 932606) from R&D Systems (McKinley Place, Minneapolis, USA) for 1 h at room temperature. Cells were washed after each extracellular and intracellular staining

with PBS containing 1% FCS, 2 mM EDTA, and 1% sodium azide. Cells were acquired using LSR Fortessa flow cytometer with the DIVA software and were further analyzed using FlowJo 10.6.2 software (all from BD Biosciences, Franklin Lakes, New Jersey, USA). Compensation was measured, and calculated by FACS DIVA software. For exclusion of leukocytes in the monocyte panel the following marker were stained: CD3, CD15, CD19 CD20, and CD56. Monocytes were gated as follows: time/ singlets/ leukocytes/ living cells/ lineage negative cells/ CD14CD16. Classical monocytes were characterized as $CD14^{+}CD16^{-}$, intermediate as $CD14^{+}CD16^{+}$ and non-classical as $CD14^{dim}CD16^{+}$ (**supplemental figure S4**). Platelets were characterized according to their size and granularity and gated as follows: time/ singlets/ platelets/ living platelets FSC-A. In particular, platelets were gated as follows: FSC-A/$CD42b^{+}$ (**supplemental figure S5**). Surface marker expression was quantified as median fluorescence intensity (MFI), and frequency of living cells with fluorescence minus one (FMO) controls used to set the cut-off for positive staining. Manual gating schemes of monocyte subsets and platelets are displayed in **supplemental figure S4 and S5**, respectively.

Unsupervised data analysis as detailed below was done using OMIQ data analysis software (Omiq inc., Santa Clara, CA, USA). First, the data were manually gated to remove aggregates, dead cells, debris, and then the data were sub-sampled to include $1.5x10^{6}$ $CD42b^{+}$ cells/group (platelets) or $1.0x10^{6}$ $CD14^{+}$/$CD16^{+}$/$CD14^{+}CD16^{+}$ cells/group. Next, flowAI[30, 32] was run to check for any aberrant regions of the files. FlowAI settings were as follows: all files used, all fluorescent channels and time selected, all methods used and default settings. Subsequently, dimension reduction analysis was performed using Uniform Manifold Approximation and Projection (UMAP) to visualize the different sub-populations of the cells. UMAP settings for platelets were as follows: all files used, all fluorescent parameters were used besides Live/Dead, Neighbors = 15, Minimum Distance = 0.4, Components = 2, Metric = Euclidean, Learning Rate = 1, Epochs = 200, Random Seed = 2588, Embedding Initialization = spectral. For monocytes following UMAP settings were used: all files used, all fluorescent parameters were used, Neighbors=15, Minimum Distance=0.4, Components=2, Metric=Euclidean, Learning Rate=1, Epochs=200, Random Seed=8263, Embedding Initialization = spectral.

Following the UMAP analysis, PhenoGraph was run to cluster the data. PhenoGraph settings for platelets were as follows: all files used, clustering features CXCL14, CD41, CD31, CD61, CXCL12, CD62P, MIF, CD42b, umap_1, umap_2, Distance Metric=euclidian, consensus metaclustering with k=20, Louvain Seed=2737. PhenoGraph settings for monocytes were as follows: all files used, clustering features CXCL14, CXCR7, CD11b, CXCR4, CD16, CCR7, CD14, CXCL12, CD62L, MIF, HLA-DR, (monocyte Panel 2: CD106, CD40, CCR2, CD11a, CD16, CD80, CD14, CD54, CD49a, CX3CR1, HLA-DR,) umap_1, umap_2, Distance Metric =euclidian, consensus metaclustering with k=20, Louvain Seed=9898. A heatmap was generated with the metaclusters obtained from PhenoGraph and clustered hierarchically on all surface markers with a euclidean distance metric to indicate the similarity of the populations (**supplemental Figures S4 – S9**).

ELISA MIF and TGFß-1

Human MIF or TGFß-1 plasma levels from frozen plasma samples were quantified using an enzyme-linked immunosorbent assays (ELISA) (Biolegend, San Diego, California, USA or from R&D Systems (McKinley, Minneapolis, USA). The assays were performed according to the manufacturer's manual.

Measurement of plasma levels of cytokines and chemokines (LEGENDPlex)

The concentrations of specific cytokines, and chemokines of frozen plasma samples was determined by LEGENDPlex Inflammation Panel 1 and LEGENDPlex Proinflammatory Chemokine Panel (BioLegend, San Diego, California, USA). The assays were performed according to the manufacturer's manual. For the measurement, FACS Lyric (BD Biosciences, Franklin Lakes, New Jersey, USA) was used, and data analysis was performed with the LEGENDPlex Data Analysis Software (BioLegend, San Diego, California, USA).

In vitro cell culture of human valvular interstitial cells

To address the functional consequences of MIF for disease progression of aortic stenosis, we performed *in vitro* cell culture experiments. We analyzed the effect of human recombinant MIF and the MIF antagonist ibudilast on inflammatory and osteogenic changes of valvular interstitial cells (VICs). The inflammatory phenotype of VICs was defined by the increased expression of vimentin and α-SMA. To mimic an either inflammatory or osteogenic driven environment we performed the cell culture experiments with pro-inflammatory medium and pro-osteogenic medium, respectively, to either influence a differentiation towards an inflammatory versus an osteogenic phenotype as established and described in previous studies. As osteogenic differentiation of VICs is characterized by increased activity and expression of alkaline phosphatase (ALP), we also analyzed ALP expression in *in vitro* cell culture. In brief, human VICs (Lonza Cat: 00225974, LotNr: 1F5268) plated in 12-well plates were cultured in inflammatory or osteogenic medium (MSCGM Mesenchymal Stem Cell Growth medium BulletKit # PT-3001) containing, DMEM with 10% FBS, 1% penicillin/streptomycin (Sigma), 10 nmol/L dexamethasone (MP Biomedicals, Santa Ana, CA), 10 mmol/L b-glycerophosphate (EMD Millipore, Burlington, MA), and 50 µg/mL L-ascorbic acid (Sigma-Aldrich), or pro-inflammatory medium containing, DMEM with 5% FBS, 1% penicillin/streptomycin (Sigma), 2 mM L-glutamine, 50 ng/mL insulin, 10 ng/mL, recombinant fibroblast growth factor-2 (FGF-2, Sigma-Aldrich) for 7 days as described before. VICs were incubated with either human recombinant MIF protein (R&D, # MAB289-MF), the MIF antagonist ibudilast (Sigma-Aldrich I0157), or the combination of both over 7 days before harvest and analysis. For analysis, cells were washed with PBS, VICs were counted, and live/dead stain was performed with Zombie NIR from BioLegend (San Diego, California, USA). Afterwards, the cells were permeabilized in 10x Perm buffer (Thermo Fisher Scientific, Waltham, Massachusetts, USA) followed by an intracellular staining wit anti-Vimentin AF647 (R&D, # MAB210, anti-alpha smooth muscle actin (αSMA) AF488 (R&D, # MAB1420), and anti-alkaline phosphatase (ALP) PE (R&D, MAB1448). Cells were acquired using LSR II Fortessa flow cytometer with the DIVA software and were further analyzed using FlowJo 10.6.2 software (all from BD Biosciences, Franklin

Lakes, New Jersey, USA). Compensation was measured, and calculated by FACS DIVA software.

Supplemental Table 1. Detailed baseline characteristics of patient population

**Parameters**	**All Patients, N=475**	**SP-AS, N=238**	**FP-AS, N=237**	**p-value**
Clinical characteristics				
Age, y	77 (69-82)	79 (71-83)	76 (69-81)	**<0.001**
Male	287 (60.4)	139 (58.4)	148 (62.4)	0.368
BMI (kg/m²)	27.1 (24.3-31.1)	27.2 (24.1-31.2)	27.0 (24.4-31.0)	0.725
Systolic blood pressure (mmHg)	135 (120-150)	140 (120-150)	133 (120-150)	0.728
NYHA class > 2	258 (54.3)	129 (54.2)	129 (54.4)	0.788
Cardiovascular risk factors and co-morbidities				
Coronary artery disease				
- 1-vessel	80 (16.8)	36 (15.1)	44 (18.6)	0.850
- 2-vessel	69 (14.5)	36 (15.1)	33 (13.9)	
- 3-vessel	95 (20)	48 (20.2)	47 (19.8)	
- CABG	23 (4.8)	13 (5.5)	10 (4.2)	
Myocardial infarction	32(15.8)	19 (14.7)	13 (16.9)	0.230
Smoking	75 (15.8)	35 (14.7)	40 (16.9)	0.615
Hyperlipidemia	224 (47.2)	118 (49.6)	106 (44.7)	0.244
Diabetes mellitus	116 (24.4)	59 (24.8)	57 (24.1)	0.960
Hypertension	387 (81.5)	195 (81.9)	192 (81.0)	0.871
Atrial fibrillation	161 (33.9)	94 (39.5)	67 (28.3)	**0.012**
Pulmonary hypertension (mmHg)	208 (43.8)	111 (46.6)	97 (40.9)	0.231
Bicuspid aortic valve	33 (6.9)	14 (5.9)	19 (8.0)	0.353
STS-Score	2.8 (1.6-6.7)	2.8 (1.6-10.1)	2.7 (1.5-5.2)	**0.021**
Parameters of echocardiography and electrocardiography				
Left ventricular ejection fraction,	60 (46-60)	60 (49-60)	60 (45-60)	0.892
Stroke volume (mL)	76.8 (60-96.5)	73.1 (60.5-91.9)	81.9 (58.9-100)	0.060
Stroke volume index (mL/m^2)	40.6 (31.7-52.5)	39.4 (32.2-49.5)	44 (30.8-57.2)	0.083
Mean pressure gradient (mmHg)	42 (32-50)	43 (33-50.3)	41 (31-50)	0.458
Peak pressure gradient (mmHg)	70 (56-84)	71 (59-84)	68.5 (54-85)	0.220
AVA (cm^2)	0.7 (0.6-0.9)	0.7 (0.6-0.9)	0.7 (0.6-0.9)	0.616
Heart Rate (bpm)	72.5 (64-85)	72.5 (65-84)	72.5 (64-87)	0.875
Medication at study entry				
ASA	229 (48.2)	114 (47.9)	115 (48.5)	0.721
Antiplatelet therapy	82 (17.3)	41 (17.2)	41 (17.3)	0.877
Oral anticoagulation	105 (22.1)	60 (25.2)	45 (19)	0.103
Antihypertensive therapy	304 (64.0)	153 (64.3)	151 (63.7)	0.950
Aldosterone inhibitors	76 (16.0)	38 (15.9)	38 (16.0)	0.964
Diuretics	234 (49.3)	118 (49.6)	116 (48.9)	0.974
Beta blockers	229 (48.2)	117 (49.2)	112 (47.2)	0.702
Statins	256 (53.9)	132 (55.5)	124 (52.3)	0.499

Laboratory parameters and biomarkers				
Leukocytes (1000/µL)	7100 (6090-8485)	7000 (5920-8300)	7200 (6245-8660)	0.200
Hb (g/dL)	12.8 (11.5-13.9)	12.7 (11.3-13.9)	12.9 (11.7-13.9)	0.397
Platelets (1000/µL)	212 (181-259)	212 (181-247.5)	212 (180-265)	0.460
GFR-MDRD (ml/m^2)	70 (53-85.4)	68.8 (52.5-85.3)	73.2 (54-85.7)	0.190
CRP (mg/dL)	1 (0.8-1.2)	0.23 (0.1-0.85)	0.32 (0.1-0.95)	0.233
hs TNI (mg/L)	0.06 (0.03-5)	0.06 (0.03-2.5)	0.06 (0.03-10)	0.524
NT-pro-BNP (ng/L)	429 (203-1190)	429 (221-1059)	400.5 (156.3-1350)	0.486
Serum Creatinine (mg/dL)	1 (0.8-1.2)	1.0 (0.8-1.2)	0.95 (0.8-1.2)	0.516
Total Cholesterol (mg/dL)	171 (141.8-203)	165 (140-200)	175 (142.8-211)	0.161
LDL (mg/dL)	96 (69.3-129)	90 (68-126)	102 (72-129)	0.250

Values are given as numbers (n) and percentage (%) or are given as median and interquartile range (IQR). AS – aortic stenosis, ASA – Acetylsalicylic acid , AV – aortic valve, AVA – aortic valve area, BMI – body mass index, CAD – coronary artery disease, CABG - coronary artery bypass grafting, CK – creatinine kinase, CRP – C-reactive protein, GFR-MDRD – glomerular filtration rate, Hb – hemoglobin, HDL – high density lipoprotein, hs TNI - High sensitive Troponin I, LDL – low density lipoprotein, NT-pro-BNP – N-terminal pro-B-type natriuretic peptide, STS - Society of Thoracic Surgeons.

Supplemental Table S2. Nano-String mRNA profiling. Complete list of the 594 genes tested with name and accession number in alphabetical order

Gene name	Accession #
**ABCB1**	NM_000927.3
**ABL1**	NM_005157.3
**ADA**	NM_000022.2
**AHR**	NM_001621.3
**AICDA**	NM_020661.1
**AIRE**	NM_000383.2
**APP**	NM_000484.3
**ARG1**	NM_000045.2
**ARG2**	NM_001172.3
**ARHGDIB**	NM_001175.4
**ATG10**	NM_001131028.1
**ATG12**	NM_004707.2
**ATG16L1**	NM_198890.2
**ATG5**	NM_004849.2
**ATG7**	NM_001136031.2
**ATM**	NM_000051.3
**B2M**	NM_004048.2
**B3GAT1**	NM_018644.3
**BATF**	NM_006399.3
**BATF3**	NM_018664.2
**BAX**	NM_138761.3
**BCAP31**	NM_005745.7
**BCL10**	NM_003921.2
**BCL2**	NM_000657.2
**BCL2L11**	NM_138621.4
**BCL3**	NM_005178.2
**BCL6**	NM_001706.2
**BID**	NM_001196.2
**BLNK**	NM_013314.2
**BST1**	NM_004334.2

Gene name	Accession #
**BST2**	NM_004335.2
**BTK**	NM_000061.1
**BTLA**	NM_181780.2
**C14orf166**	NM_016039.2
**C1QA**	NM_015991.2
**C1QB**	NM_000491.3
**C1QBP**	NM_001212.3
**C1R**	NM_001733.4
**C1S**	NM_001734.2
**C2**	NM_000063.3
**C3**	NM_000064.2
**C4A/B**	NM_007293.2
**C4BPA**	NM_000715.3
**C5**	NM_001735.2
**C6**	NM_000065.2
**C7**	NM_000587.2
**C8A**	NM_000562.2
**C8B**	NM_000066.2
**C8G**	NM_000606.2
**C9**	NM_001737.3
**CAMP**	NM_004345.3
**CARD9**	NM_052813.4
**CASP1**	NM_001223.3
**CASP10**	NM_032977.3
**CASP2**	NM_032982.2
**CASP3**	NM_032991.2
**CASP8**	NM_001228.4
**CCBP2**	NM_001296.3
**CCL11**	NM_002986.2
**CCL13**	NM_005408.2

Gene name	Accession #
**CCL15**	NM_032965.3
**CCL16**	NM_004590.2
**CCL18**	NM_002988.2
**CCL19**	NM_006274.2
**CCL2**	NM_002982.3
**CCL20**	NM_004591.1
**CCL22**	NM_002990.3
**CCL23**	NM_145898.1
**CCL24**	NM_002991.2
**CCL26**	NM_006072.4
**CCL3**	NM_002983.2
**CCL4**	NM_002984.2
**CCL5**	NM_002985.2
**CCL7**	NM_006273.2
**CCL8**	NM_005623.2
**CCND3**	NM_001760.2
**CCR1**	NM_001295.2
**CCR10**	NM_016602.2
**CCR2**	NM_001123041.2
**CCR5**	NM_000579.1
**CCR6**	NM_031409.2
**CCR7**	NM_001838.2
**CCR8**	NM_005201.2
**CCRL1**	NM_016557.2
**CCRL2**	NM_003965.4
**CD14**	NM_000591.2
**CD160**	NM_007053.2
**CD163**	NM_004244.4
**CD164**	NM_006016.4
**CD19**	NM_001770.4

Gene name	Accession #
**CD1A**	NM_001763.2
**CD1D**	NM_001766.3
**CD2**	NM_001767.3
**CD209**	NM_021155.2
**CD22**	NM_001771.2
**CD24**	NM_013230.2
**CD244**	NM_016382.2
**CD247**	NM_198053.1
**CD27**	NM_001242.4
**CD274**	NM_014143.3
**CD276**	NM_001024736.1
**CD28**	NM_001243078.1
**CD34**	NM_001025109.1
**CD36**	NM_001001548.2
**CD3D**	NM_000732.4
**CD3E**	NM_000733.2
**CD3EAP**	NM_012099.1
**CD4**	NM_000616.4
**CD40**	NM_001250.4
**CD40LG**	NM_000074.2
**CD44**	NM_001001392.1
**CD45R0**	NM_080921.3
**CD45RA**	NM_002838.4
**CD45RB**	ENST00000367367.1
**CD46**	NM_172350.1
**CD48**	NM_001778.2
**CD5**	NM_014207.2

Gene name	Accession #
**CD59**	NM_000611.4
**CD6**	NM_006725.3
**CD7**	NM_006137.6
**CD70**	NM_001252.2
**CD74**	NM_001025159.1
**CD79A**	NM_001783.3
**CD79B**	NM_021602.2
**CD80**	NM_005191.3
**CD81**	NM_004356.3
**CD82**	NM_002231.3
**CD83**	NM_004233.3
**CD86**	NM_175862.3
**CD8A**	NM_001768.5
**CD8B**	NM_004931.3
**CD9**	NM_001769.2
**CD96**	NM_005816.4
**CD97**	NM_078481.2
**CD99**	NM_002414.3
**CDH5**	NM_001795.3
**CDKN1A**	NM_000389.2
**CEACAM1**	NM_001712.3
**CEACAM6**	NM_002483.4
**CEACAM8**	NM_001816.3
**CEBPB**	NM_005194.2
**CFB**	NM_001710.5
**CFD**	NM_001928.2
**CFH**	NM_001014975.2

Gene name	Accession #
**CIITA**	NM_000246.3
**CISH**	NM_145071.2
**CLEC4A**	NM_194448.2
**CLEC4E**	NM_014358.2
**CLEC5A**	NM_013252.2
**CLEC6A**	NM_001007033.1
**CLEC7A**	NM_197954.2
**CLU**	NM_001831.2
**CMKLR1**	NM_004072.1
**CR1**	NM_000651.4
**CR2**	NM_001006658.1
**CRADD**	NM_003805.3
**CSF1**	NM_000757.4
**CSF1R**	NM_005211.2
**CSF2**	NM_000758.2
**CSF2RB**	NM_000395.2
**CSF3R**	NM_156038.2
**CTLA4-TM**	NM_005214.3
**CTLA4_all**	NM_005214.3
**CTNNB1**	NM_001098210.1
**CTSC**	NM_001814.4
**CTSG**	NM_001911.2
**CTSS**	NM_004079.3
**CUL9**	NM_015089.2
**CX3CL1**	NM_002996.3
**CX3CR1**	NM_001337.3
**CXCL1**	NM_001511.1

**CD53**	NM_001040033.1	**CFI**	NM_000204.3	**CXCL10**	NM_001565.1
**CD55**	NM_000574.3	**CFP**	NM_002621.2	**CXCL11**	NM_005409.4
**CD58**	NM_001779.2	**CHUK**	NM_001278.3	**CXCL12**	NM_000609.5

1

**Gene name**	**Accession #**	**Gene name**	**Accession #**	**Gene name**	**Accession #**
**CXCL13**	NM_006419.2	**FCGR2B**	NM_001002273.1	**HLA-DQB1**	NM_002123.3
**CXCL2**	NM_002089.3	**FCGR3A/B**	NM_000570.4	**HLA-DRA**	NM_019111.3
**CXCL9**	NM_002416.1	**FCGRT**	NM_004107.4	**HLA-DRB1**	NM_002124.2
**CXCR1**	NM_000634.2	**FKBP5**	NM_001145775.1	**HLA-DRB3**	NM_022555.3
**CXCR2**	NM_001557.2	**FN1**	NM_212482.1	**HRAS**	NM_005343.2
**CXCR3**	NM_001504.1	**FOXP3**	NM_014009.3	**ICAM1**	NM_000201.2
**CXCR4**	NM_003467.2	**FYN**	NM_002037.3	**ICAM2**	NM_000873.3
**CXCR6**	NM_006564.1	**GATA3**	NM_001002295.1	**ICAM3**	NM_002162.3
**CYBB**	NM_000397.3	**GBP1**	NM_002053.1	**ICAM4**	NM_001039132.1
**DEFB1**	NM_005218.3	**GBP5**	NM_052942.3	**ICAM5**	NM_003259.3
**DEFB103A**	NM_001081551.2	**GFI1**	NM_005263.2	**ICOS**	NM_012092.2
**DEFB103B**	NM_018661.3	**GNLY**	NM_006433.2	**ICOSLG**	NM_015259.4
**DEFB4A**	NM_004942.2	**GP1BB**	NM_000407.4	**IDO1**	NM_002164.3
**DPP4**	NM_001935.3	**GPI**	NM_000175.2	**IFI16**	NM_005531.1
**DUSP4**	NM_057158.2	**GPR183**	NM_004951.3	**IFI35**	NM_005533.3
**EBI3**	NM_005755.2	**GZMA**	NM_006144.2	**IFIH1**	NM_022168.2
**EDNRB**	NM_003991.2	**GZMB**	NM_004131.3	**IFIT2**	NM_001547.4
**EGR1**	NM_001964.2	**GZMK**	NM_002104.2	**IFITM1**	NM_003641.3
**EGR2**	NM_000399.3	**HAMP**	NM_021175.2	**IFNA1/13**	NM_024013.1
**ENTPD1**	NM_001098175.1	**HAVCR2**	NM_032782.3	**IFNA2**	NM_000605.3
**EOMES**	NM_005442.2	**HFE**	NM_139011.2	**IFNAR1**	NM_000629.2
**ETS1**	NM_005238.3	**HLA-A**	NM_002116.5	**IFNAR2**	NM_000874.3
**FADD**	NM_003824.2	**HLA-B**	NM_005514.6	**IFNB1**	NM_002176.2
**FAS**	NM_000043.3	**HLA-C**	NM_002117.4	**IFNG**	NM_000619.2
**FCAR**	NM_133280.1	**HLA-DMA**	NM_006120.3	**IFNGR1**	NM_000416.1

**FCER1A**	NM_002001.2	**HLA-DMB**	NM_002118.3	**IGF2R**	NM_000876.1
**FCER1G**	NM_004106.1	**HLA-DOB**	NM_002120.3	**IKBKAP**	NM_003640.3
**FCGR1A/ B**	NM_000566.3	**HLA-DPA1**	NM_033554.2	**IKBKB**	NM_001556.1
**FCGR2A**	NM_021642.3	**HLA-DPB1**	NM_002121.4	**IKBKE**	NM_014002.2
**FCGR2A/ C**	NM_201563.4	**HLA-DQA1**	NM_002122.3	**IKBKG**	NM_003639.2

**Gene name**	**Accession #**	**Gene name**	**Accession #**	**Gene name**	**Accession #**
**IKZF1**	NM_006060.3	**IL21**	NM_021803.2	**IRAK4**	NM_016123.1
**IKZF2**	NM_016260.2	**IL21R**	NM_021798.2	**IRF1**	NM_002198.1
**IKZF3**	NM_183232.2	**IL22**	NM_020525.4	**IRF3**	NM_001571.5
**IL10**	NM_000572.2	**IL22RA2**	NM_181310.1	**IRF4**	NM_002460.1
**IL10RA**	NM_001558.2	**IL23A**	NM_016584.2	**IRF5**	NM_002200.3
**IL11RA**	NM_147162.1	**IL23R**	NM_144701.2	**IRF7**	NM_001572.3
**IL12A**	NM_000882.2	**IL26**	NM_018402.1	**IRF8**	NM_002163.2
**IL12B**	NM_002187.2	**IL27**	NM_145659.3	**IRGM**	NM_001145805 .1
**IL12RB1**	NM_005535.1	**IL28A**	NM_172138.1	**ITGA2B**	NM_000419.3
**IL13**	NM_002188.2	**IL28A/B**	NM_172139.2	**ITGA4**	NM_000885.4
**IL13RA1**	NM_001560.2	**IL29**	NM_172140.1	**ITGA5**	NM_002205.2
**IL15**	NM_172174.1	**IL2RA**	NM_000417.1	**ITGA6**	NM_000210.1
**IL16**	NM_004513.4	**IL2RB**	NM_000878.2	**ITGAE**	NM_002208.4
**IL17A**	NM_002190.2	**IL2RG**	NM_000206.1	**ITGAL**	NM_002209.2
**IL17B**	NM_014443.2	**IL3**	NM_000588.3	**ITGAM**	NM_000632.3
**IL17F**	NM_052872.3	**IL32**	NM_00101263 3.1	**ITGAX**	NM_000887.3
**IL18**	NM_001562.2	**IL4**	NM_000589.2	**ITGB1**	NM_033666.2
**IL18R1**	NM_003855.2	**IL4R**	NM_000418.2	**ITGB2**	NM_000211.2
**IL18RAP**	NM_003853.2	**IL5**	NM_000879.2	**ITLN1**	NM_017625.2
**IL19**	NM_013371.3	**IL6**	NM_000600.1	**ITLN2**	NM_080878.2
**IL1A**	NM_000575.3	**IL6R**	NM_000565.2	**JAK1**	NM_002227.1
**IL1B**	NM_000576.2	**IL6ST**	NM_002184.2	**JAK2**	NM_004972.2
**IL1R1**	NM_000877.2	**IL7**	NM_000880.2	**JAK3**	NM_000215.2
**IL1R2**	NM_173343.1	**IL7R**	NM_002185.2	**KCNJ2**	NM_000891.2
**IL1RAP**	NM_002182.2	**IL8**	NM_000584.2	**KIR3DL1**	NM_013289.2
**IL1RL1**	NM_016232.4	**IL9**	NM_000590.1	**KIR3DL2**	NM_006737.2

**IL1RL2**	NM_003854.2	**ILF3**	NM_00113767 3.1	**KIR3DL3**	NM_153443.3
**IL1RN**	NM_000577.3	**IRAK1**	NM_001569.3	**KIR_Activ ating_ Subgroup _1**	NM_001083539 .1
**IL2**	NM_000586.2	**IRAK2**	NM_001570.3	**KIR_Activ ating_S ubgroup_ 2**	NM_014512.1
**IL20**	NM_018724.3	**IRAK3**	NM_007199.1	**KIR_Inhib iting_ Subgroup _1**	NM_014218.2

1

**Gene name**	**Accession #**	**Gene name**	**Accession #**	**Gene name**	**Accession #**
**KIR_Inhib iting_ Subgroup _2**	NM_014511.3	**LILRB3**	NM_006864.2	**MSR1**	NM_002445.3
**KIT**	NM_000222.2	**LILRB4**	NM_00108143 8.1	**MUC1**	NM_001018017 .1
**KLRAP1**	NR_028045.1	**LILRB5**	NM_00108144 2.1	**MX1**	NM_002462.2
**KLRB1**	NM_002258.2	**LITAF**	NM_004862.3	**MYD88**	NM_002468.3
**KLRC1**	NM_002259.3	**LTA**	NM_000595.2	**NCAM1**	NM_000615.5
**KLRC2**	NM_002260.3	**LTB4R**	NM_181657.3	**NCF4**	NM_000631.4
**KLRC3**	NM_007333.2	**LTB4R2**	NM_019839.4	**NCR1**	NM_004829.5
**KLRC4**	NM_013431.2	**LTBR**	NM_002342.1	**NFATC1**	NM_172389.1
**KLRD1**	NM_002262.3	**LTF**	NM_002343.2	**NFATC2**	NM_012340.3
**KLRF1**	NM_016523.1	**LY96**	NM_015364.2	**NFATC3**	NM_004555.2
**KLRF2**	NM_00119076 5.1	**MAF**	NM_005360.4	**NFIL3**	NM_005384.2
**KLRG1**	NM_005810.3	**MALT1**	NM_006785.2	**NFKB1**	NM_003998.2
**KLRG2**	NM_198508.2	**MAP4K1**	NM_007181.3	**NFKB2**	NM_002502.2
**KLRK1**	NM_007360.1	**MAP4K2**	NM_004579.2	**NFKBIA**	NM_020529.1
**LAG3**	NM_002286.5	**MAP4K4**	NM_004834.3	**NFKBIZ**	NM_001005474 .1
**LAIR1**	NM_002287.3	**MAPK1**	NM_138957.2	**NLRP3**	NM_001079821 .2
**LAMP3**	NM_014398.3	**MAPK11**	NM_002751.5	**NOD1**	NM_006092.1
**LCK**	NM_005356.2	**MAPK14**	NM_001315.1	**NOD2**	NM_022162.1

**LCP2**	NM_005565.3	**MAPKAPK2**	NM_004759.3	**NOS2**	NM_000625.4
**LEF1**	NM_016269.3	**MARCO**	NM_006770.3	**NOTCH1**	NM_017617.3
**LGALS3**	NM_001177388.1	**MASP1**	NM_139125.3	**NOTCH2**	NM_024408.3
**LIF**	NM_002309.3	**MASP2**	NM_139208.1	**NT5E**	NM_002526.2
**LILRA1**	NM_006863.1	**MBL2**	NM_000242.2	**PAX5**	NM_016734.1
**LILRA2**	NM_006866.2	**MBP**	NM_002385.2	**PDCD1**	NM_005018.1
**LILRA3**	NM_006865.3	**MCL1**	NM_021960.3	**PDCD1LG2**	NM_025239.3
**LILRA4**	NM_012276.3	**MIF**	NM_002415.1	**PDCD2**	NM_144781.2
**LILRA5**	NM_181879.2	**MME**	NM_000902.2	**PDGFB**	NM_033016.2
**LILRA6**	NM_024318.2	**MR1**	NM_001531.2	**PDGFRB**	NM_002609.3
**LILRB1**	NM_001081637.1	**MRC1**	NM_002438.2	**PECAM1**	NM_000442.3
**LILRB2**	NM_005874.1	**MS4A1**	NM_152866.2	**PIGR**	NM_002644.2

**Gene name**	**Accession #**	**Gene name**	**Accession #**	**Gene name**	**Accession #**
**PLA2G2A**	NM_000300.2	**RARRES3**	NM_004585.3	**STAT5B**	NM_012448.3
**PLA2G2E**	NM_014589.1	**RELA**	NM_021975.2	**STAT6**	NM_003153.3
**PLAU**	NM_002658.2	**RELB**	NM_006509.2	**SYK**	NM_003177.3
**PLAUR**	NM_001005376.1	**RORC**	NM_001001523.1	**TAGAP**	NM_054114.3
**PML**	NM_002675.3	**RUNX1**	NM_001754.4	**TAL1**	NM_003189.2
**POU2F2**	NM_002698.2	**S100A8**	NM_002964.3	**TAP1**	NM_000593.5
**PPARG**	NM_015869.3	**S100A9**	NM_002965.2	**TAP2**	NM_000544.3
**PPBP**	NM_002704.2	**S1PR1**	NM_001400.3	**TAPBP**	NM_003190.4
**PRDM1**	NM_001198.3	**SELE**	NM_000450.2	**TBK1**	NM_013254.2
**PRF1**	NM_005041.3	**SELL**	NR_029467.1	**TBX21**	NM_013351.1
**PRKCD**	NM_006254.3	**SELPLG**	NM_003006.3	**TCF4**	NM_003199.1
**PSMB10**	NM_002801.2	**SERPING1**	NM_000062.2	**TCF7**	NM_003202.2
**PSMB5**	NM_001130725.1	**SH2D1A**	NM_001114937.2	**TFRC**	NM_003234.1
**PSMB7**	NM_002799.2	**SIGIRR**	NM_021805.2	**TGFB1**	NM_000660.3
**PSMB8**	NM_004159.4	**SKI**	NM_003036.2	**TGFBI**	NM_000358.2
**PSMB9**	NM_002800.4	**SLAMF1**	NM_003037.2	**TGFBR1**	NM_004612.2
**PSMC2**	NM_002803.3	**SLAMF6**	NM_001184714.1	**TGFBR2**	NM_001024847.1

**PSMD7**	NM_002811.3
**PTAFR**	NM_000952.3
**PTGER4**	NM_000958.2
**PTGS2**	NM_000963.1
**PTK2**	NM_005607.3
**PTPN2**	NM_002828.2
**PTPN22**	NM_015967.4
**PTPN6**	NM_002831.5
**PTPRC_all**	NM_080921.2
**PYCARD**	NM_013258.3
**RAF1**	NM_002880.2
**RAG1**	NM_000448.2
**RAG2**	NM_000536.3

**SLAMF7**	NM_021181.3
**SLC2A1**	NM_006516.2
**SMAD3**	NM_005902.3
**SMAD5**	NM_005903.5
**SOCS1**	NM_003745.1
**SOCS3**	NM_003955.3
**SPP1**	NM_000582.2
**SRC**	NM_005417.3
**STAT1**	NM_007315.2
**STAT2**	NM_005419.2
**STAT3**	NM_139276.2
**STAT4**	NM_003151.2
**STAT5A**	NM_003152.2

**THY1**	NM_006288.2
**TICAM1**	NM_014261.1
**TIGIT**	NM_173799.2
**TIRAP**	NM_148910.2
**TLR1**	NM_003263.3
**TLR2**	NM_003264.3
**TLR3**	NM_003265.2
**TLR4**	NM_138554.2
**TLR5**	NM_003268.3
**TLR7**	NM_016562.3
**TLR8**	NM_016610.2
**TLR9**	NM_017442.2
**TMEM173**	NM_198282.1

**Gene name**	**Accession #**
**TNF**	NM_000594.2
**TNFAIP3**	NM_006290.2
**TNFAIP6**	NM_007115.2
**TNFRSF10C**	NM_003841.3
**TNFRSF11A**	NM_003839.2
**TNFRSF13B**	NM_012452.2
**TNFRSF13C**	NM_052945.3
**TNFRSF14**	NM_003820.2
**TNFRSF17**	NM_001192.2
**TNFRSF1B**	NM_001066.2
**TNFRSF4**	NM_003327.2
**TNFRSF8**	NM_152942.2
**TNFRSF9**	NM_001561.4
**TNFSF10**	NM_003810.2
**TNFSF11**	NM_003701.2
**TNFSF12**	NM_003809.2

**Gene name**	**Accession #**
**VCAM1**	NM_001078.3
**VTN**	NM_000638.3
**XBP1**	NM_005080.2
**XCL1**	NM_002995.1
**XCR1**	NM_005283.2
**ZAP70**	NM_001079.3
**ZBTB16**	NM_006006.4
**ZEB1**	NM_001128128.1
**sCTLA4**	NM_001037631.1
**ABCF1**	NM_001090.2
**ALAS1**	NM_000688.4
**EEF1G**	NM_001404.4
**G6PD**	NM_000402.2
**GAPDH**	NM_002046.3
**GUSB**	NM_000181.1
**HPRT1**	NM_000194.1

**TNFSF13B**	NM_006573.4
**TNFSF15**	NM_001204344.1
**TNFSF4**	NM_003326.2
**TNFSF8**	NM_001244.3
**TOLLIP**	NM_019009.2
**TP53**	NM_000546.2
**TRAF1**	NM_005658.3
**TRAF2**	NM_021138.3
**TRAF3**	NM_145725.1
**TRAF4**	NM_004295.2
**TRAF5**	NM_004619.3
**TRAF6**	NM_145803.1
**TYK2**	NM_003331.3
**UBE2L3**	NM_198157.1

**OAZ1**	NM_004152.2
**POLR1B**	NM_019014.3
**POLR2A**	NM_000937.2
**PPIA**	NM_021130.2
**RPL19**	NM_000981.3
**SDHA**	NM_004168.1
**TBP**	NM_001172085.1
**TUBB**	NM_178014.2

Supplemental Table 3. Linear regression analysis identifies liquid biopsy as independent predictors of fast progressive aortic valve stenosis

Model	Parameter	Unstandardized Coefficients		Standardized Coefficients		
		Beta	Std error	Beta	t-value	p-value
1	Liquid biopsy	0.135	0.052	0.328	2.57	**0.013**
2	Liquid biopsy	0.130	0.047	0.320	2.78	**0.008**
3	Liquid biopsy	0.130	0.057	0.321	2.272	**0.030**
4	Liquid biopsy	0.135	0.061	0.332	2.222	**0.034**

Adjusted Beta Coefficients are given. Dependent Variable: Fast progressive aortic valve stenosis.

Model 1: Unadjusted. **Model 2:** Adjusted for age, STS score, presence of atrial fibrillation. **Model 3:** Adjusted for age, STS score, presence of atrial fibrillation, LVEF, smoking status, HTN, HLP, DM, CRP, NYHA class, and smoking status. **Model 4:** Adjusted for age, STS score, presence of atrial fibrillation, LVEF, smoking status, HTN, HLP, DM, CRP, NYHA class, and smoking status, CAD, and ASA. Liquid biopsy remained significantly associated with fast progressive aortic valve stenosis in all models shown. P-values < 0.05 were defined as statistically significant. Significant p-values are bolded. Abbreviations: ASA – Acetylsalicylic acid, CAD – coronary artery disease, CRP – C-reactive protein, DM – diabetes mellitus, HLP – hyperlipidemia, HTN – arterial hypertension, LVEF – left ventricular ejection fraction, NYHA - New York Heart Association, Std. Error - Standard Error, STS - Society of Thoracic Surgeons.

Supplemental Table 4. Cox regression analysis identifies liquid biopsy and age as independent predictors of fast progressive aortic valve stenosis

Variable	Cox regression analysis	
	**HR (95% CI)**	**p-value**
Age	0.88 (0.82 – 0.95)	**<0.001**
Liquid biopsy	0.366 (0.157 – 0.855)	**0.020**
NYHA class > 2	4.64 (0.85 – 25.37)	0.077
ASA therapy	0.433 (0.143 – 1.312)	0.139
STS Score	0.98 (0.67 – 1.42)	0.897
Atrial fibrillation	1.37 (0.27–6.86)	0.705
Gender	0.682 (0.21 – 2.24)	0.528
Hyperlipidemia	1.6 (0.31 – 8.3)	0.575

Values are given in order of decreasing hazard ratio in cox regression analysis. P-values < 0.05 were defined as statistically significant. Significant p-values are bolded. Abbreviations: AV – aortic valve, ASA – acetylic salicylic acid, CI – confidence interval, HR - hazard ratio.

Supplemental Figure S1

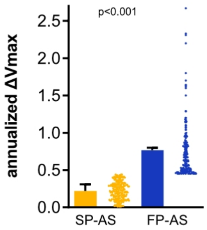

Supplemental Figure S1. Determination of fast and slow progressive aortic stenosis. Two subgroups of patients with symptomatic AS regarding fast and slow disease progression were identified by repetitive transthoracic echocardiographic assessment in a retrospective analysis. The median of annualized ΔVmax was used as cut-off and thereby two subgroups of patients were evident with either FP-AS (ΔVmax ≥0.45 m/s/year) or SP-AS (ΔVmax <0.45 m/s/year) until onset of severe symptoms occurred that warranted surgical valve replacement. Plot shows calculation and distribution of annualized ΔVmax measured by echocardiography of patients characterized as SP-AS (n=238) and FP-AS (n=237) (SP-AS vs FP-AS; median, IQR; 0.22 (0.12-0.31) vs 0.63 (0.49-0.84), P<0.001). Plotted: Median ± interquartile range (IQR); Statistics: Mann-Whitney U test.

Supplemental Figure S2

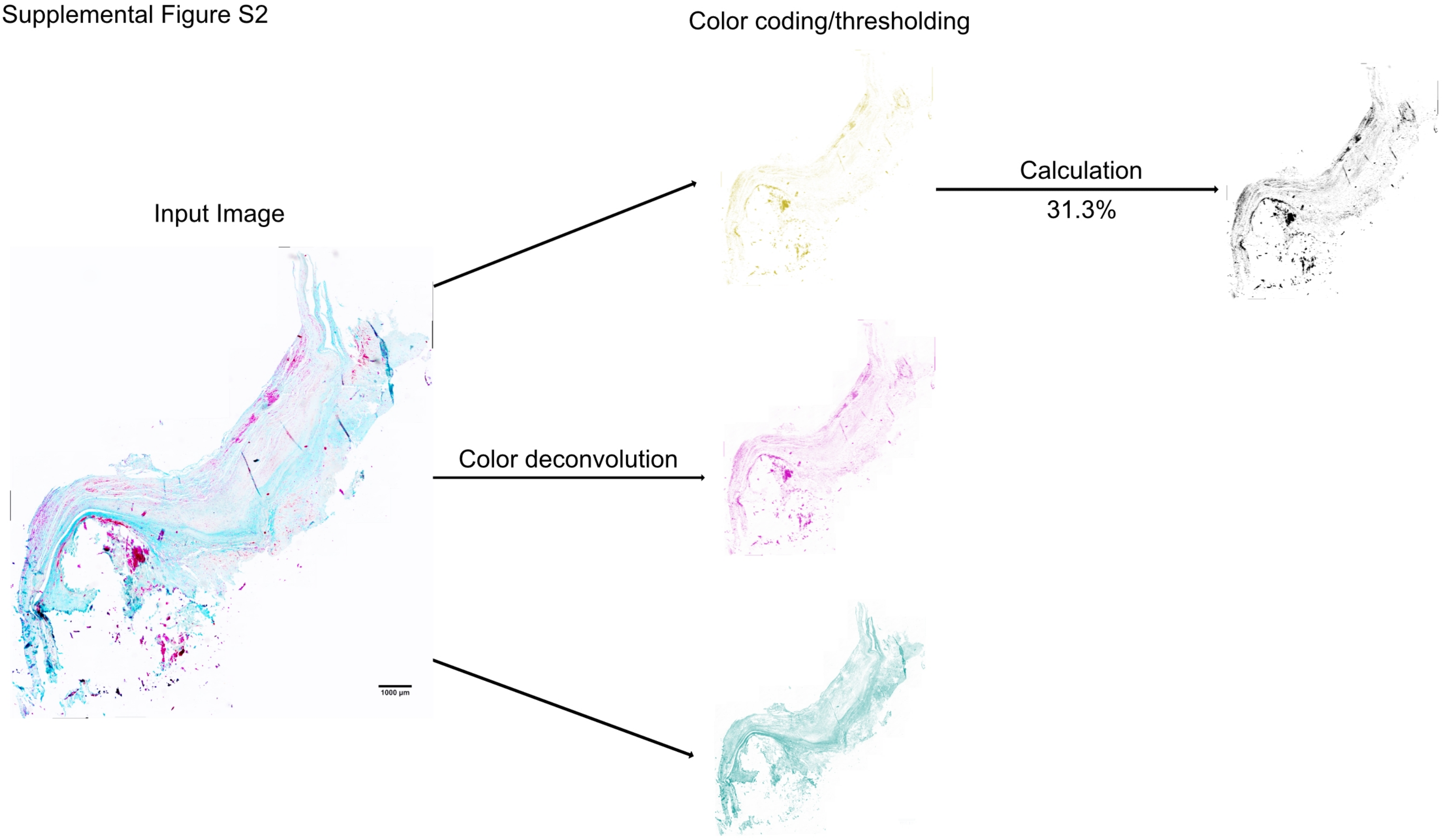

Supplemental Figure S2. Methodology and quantification of collagen deposition.

Workflow of color deconvolution with ImageJ. The color of positively and negatively stained areas, as well as the background color, were selected using the color picker. Next, the input image was split into the three colors. For the image with DAB+ areas a color thresholding was performed to calculate the area percentage or total area of positive staining in relation to whole valve tissue area. The procedure was automated using a custom batch-script and the same color and threshold values for all individuals.

Supplemental Figure S3

A

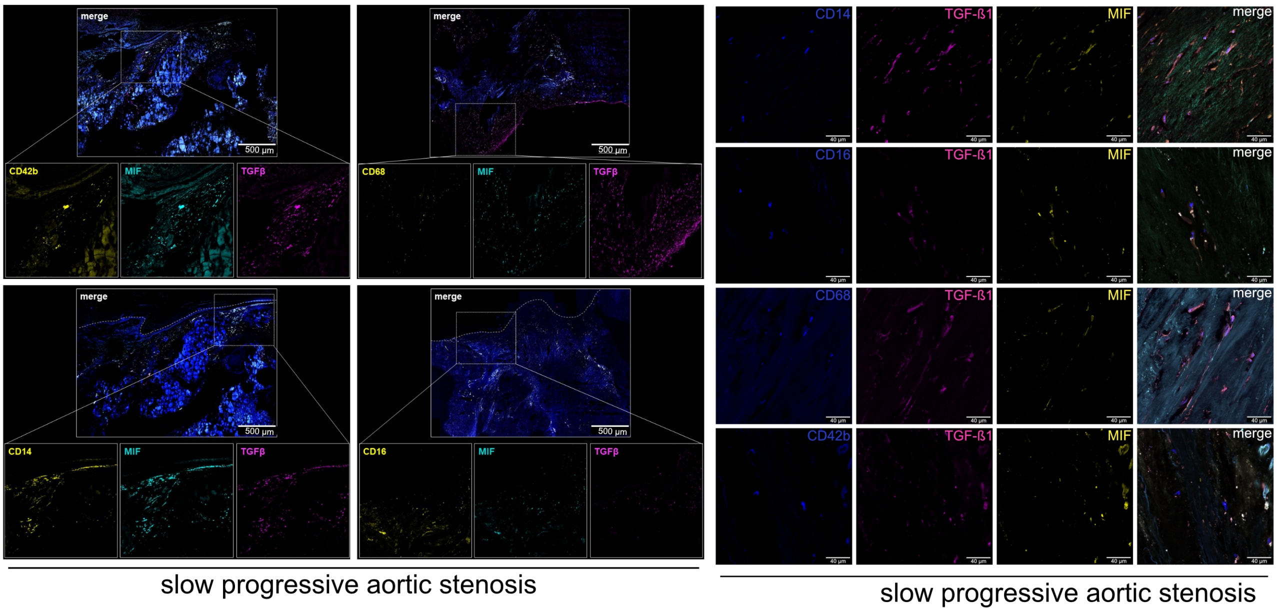

B

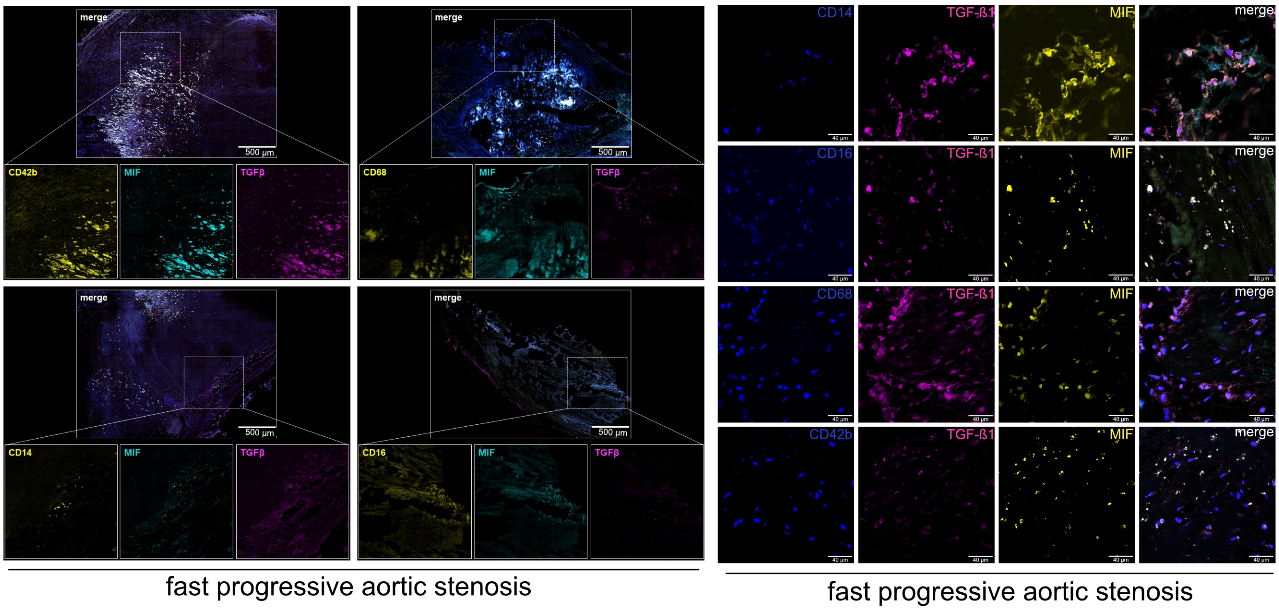

C

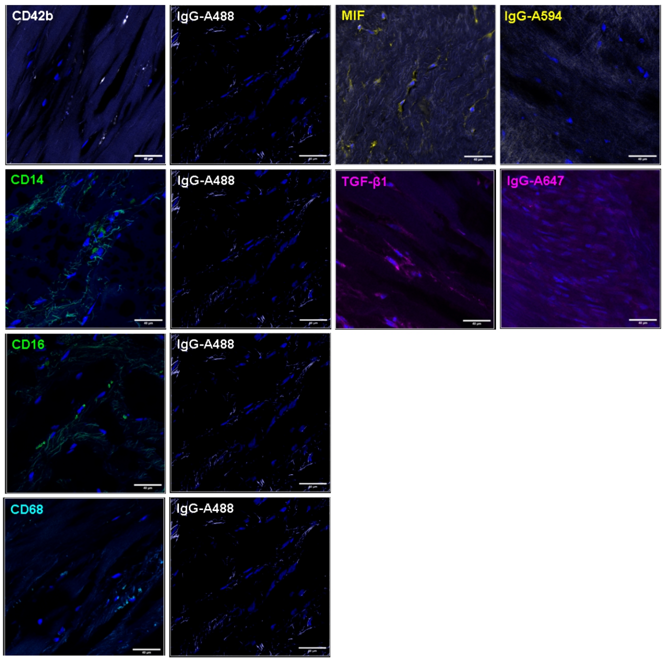

Supplemental Figure S3. Immunofluorescence microscopy of the aortic valves reveals that MIF immunoreactivity co-localizes significantly with platelet infiltration of the diseased aortic valve tissue. (A) Immunofluorescence immunohistochemistry of slow progressive aortic stenosis exemplary illustrating co-localization of CD42b positive platelets, CD68 positive macrophages and monocytes with positive staining of CD 14 or CD16 and cytokine expression of each cell type stained for MIF and TGF-ß1, respectively (overview of AV leaflet left panel, detailed illustration right panel). MIF immunoreactivity co-localizes significantly with CD42b positive areas of platelet infiltration of the diseased aortic valve tissue. Co-localization of MIF expression and monocytes/macrophages was also observed albeit to a lower degree. SP-AS is characterized by reduced platelet and immune cell infiltration compared to fast progressive aortic stenosis. **(B)** Immunofluorescence immunohistochemistry of fast progressive aortic stenosis exemplary illustrating co-localization of CD42b positive platelets, CD68 positive macrophages and monocytes with positive staining of CD 14 or CD16 and cytokine expression of each cell type stained for MIF and TGF-ß1, respectively (overview of AV leaflet left panel, detailed illustration right panel). FPAS is characterized by enhanced platelet and immune cell infiltration with higher amount of MIF and TGF-ß1 expression compared to slow progressive aortic stenosis. MIF immunoreactivity co-localizes significantly with CD42b positive areas of platelet infiltration of the diseased aortic valve tissue. Co-localization of MIF expression and monocytes/macrophages was also observed albeit to a lower degree. **(C)** Respective IgG controls for the FITC-conjugated antibodies raised against CD42b, CD14, CD16 and CD68, as well as for the Alexa594- and Alexa647-labeled antibodies against MIF and TGF-β1 in exemplary tissue sections. For immunofluorescence staining sections were incubated with the respective antibodies (CD42b-FITC, clone HPI1; CD14-FITC, clone M5E2; CD16-FITC, clone 3G8; CD68-FITC, clone Y1/82A; TGFβ1-Alexa647, clone 1018746 and MIF-Alexa594, clone 932606) for 45 min at room temperature. Samples were mounted with DAPI-containing Fluorshield (Sigma-Aldrich) to counterstain nuclei. Image acquisition was done using a TCS SP8 confocal laser scanning microscope (Leica

Microsystems CMS) with a 40x/NA 1.3 oil objective and LAS X software. Images were processed with Image J (Version 2.9.0/1.53t, NIH).

Supplemental Figure S4

A

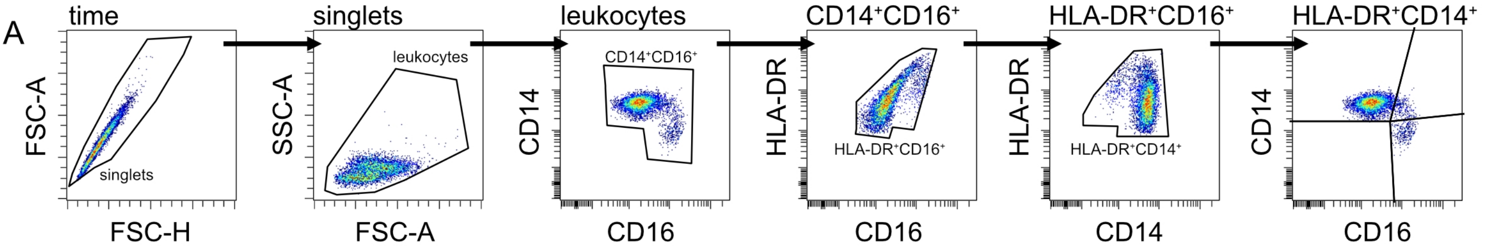

B

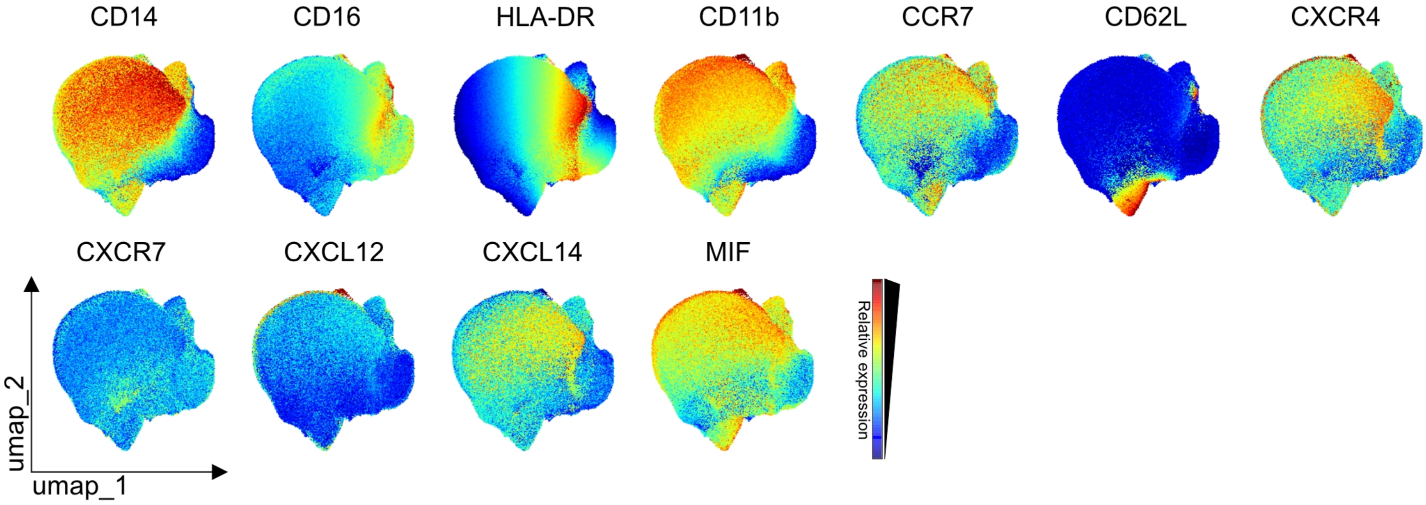

C

	CD14	CD16	HLA-DR	CD11b	CCR7	CD62L	CXCR4	CXCR7	CXCL12	CXCL14	MIF
FP-AS	1.91	1	1.96	3.09	1.18	0.23	1.85	1.89	0.96	0.99	2.61
SP-AS	1.78	0.81	1.78	3.1	1.21	0.23	1.92	2.09	0.89	0.99	2.55

Min
Max

D

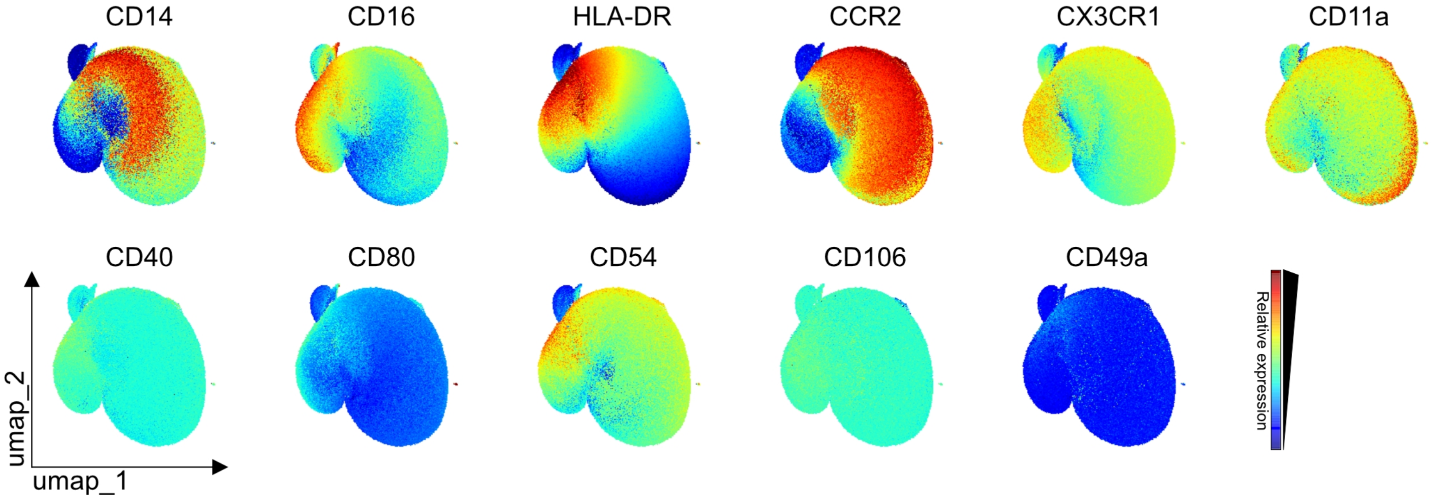

E

	CD14	CD16	HLA-DR	CCR2	CX3CR1	CD11a	CD40	CD80	CD54	CD106	CD49a
FP-AS	1.65	1.67	2.04	3.74	2.92	2.7	0.42	0.6	2.35	0.61	0.19
SP-AS	1.58	1.67	2	3.85	2.89	2.63	0.43	0.62	2.38	0.61	0.21

Min
Max

Supplemental Figure S4. Gating strategy of human monocytes and UMAP analysis of surface marker expression. (A) Representative gating strategy of human monocytes. Gating strategy was used for both monocyte flow cytometry panels. Classical monocytes were characterized as $CD14^{+}CD16^{-}$, intermediate as $CD14^{+}CD16^{+}$ and non-classical as $CD14^{dim}CD16^{+}$. **(B+D)** UMAP plots representing median marker expression of monocyte flow cytometry panel 1 **(B)** or monocyte flow cytometry panel 2 **(D)** as overlay of all patient samples. **(C+E)** Heatmap of depicted marker expression (median). Patients are clustered into slow and fast progressive aortic stenosis. **(A-E)** Plots were generated using OMIQ data analysis software.

Supplemental Figure S5

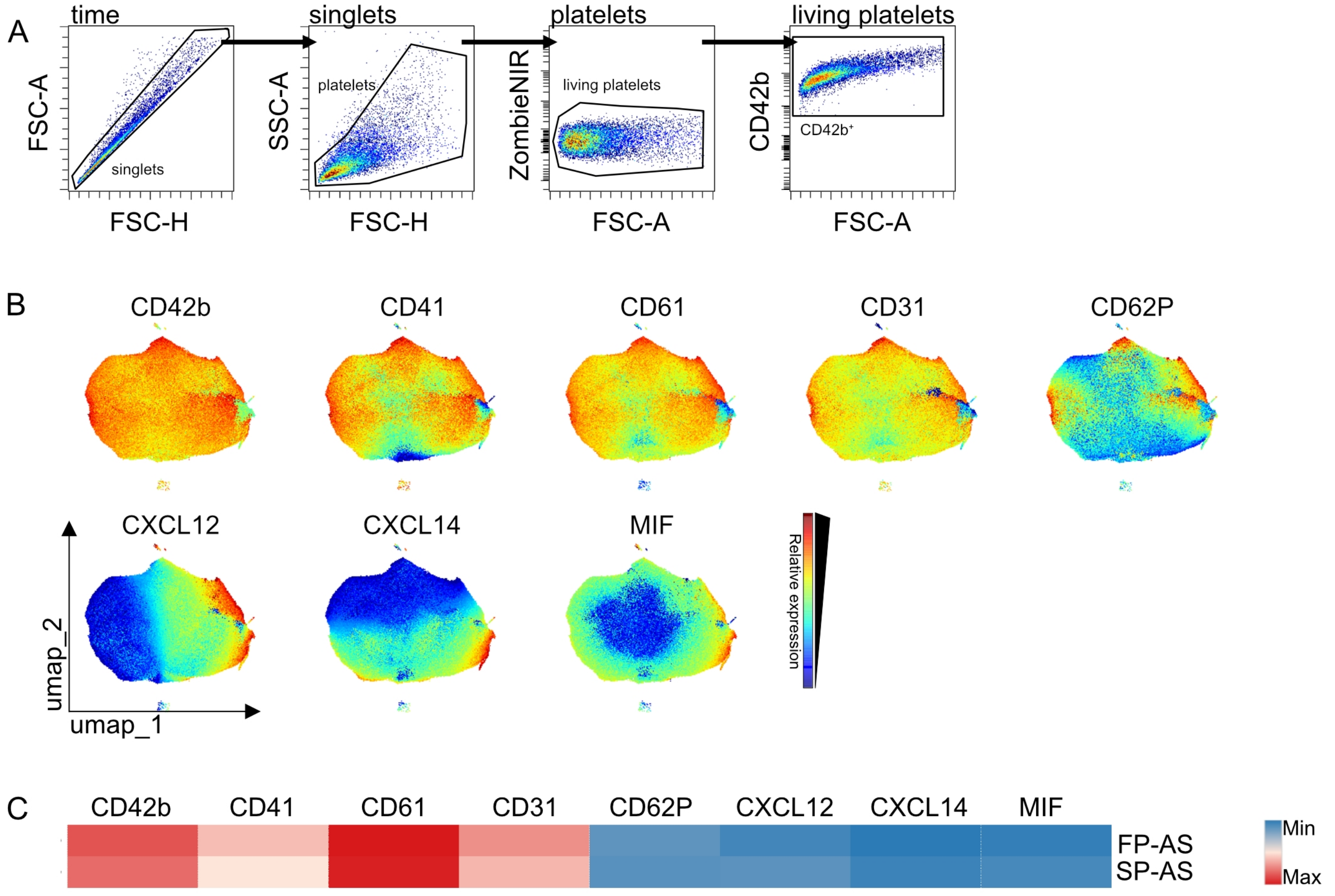

Supplemental Figure S5. Gating strategy of human platelets and UMAP analysis of surface marker expression. (A) Manual gating strategy of human platelets. **(B)** UMAP plots representing median marker expression of platelets as overlay of all patient samples. **(C)** Heatmap of median expression of analyzed markers of platelet flow cytometry panel. Patients are divided into slow and fast progressive aortic stenosis. **(A-C)** Plots were generated using OMIQ data analysis software.

Supplemental Figure S6

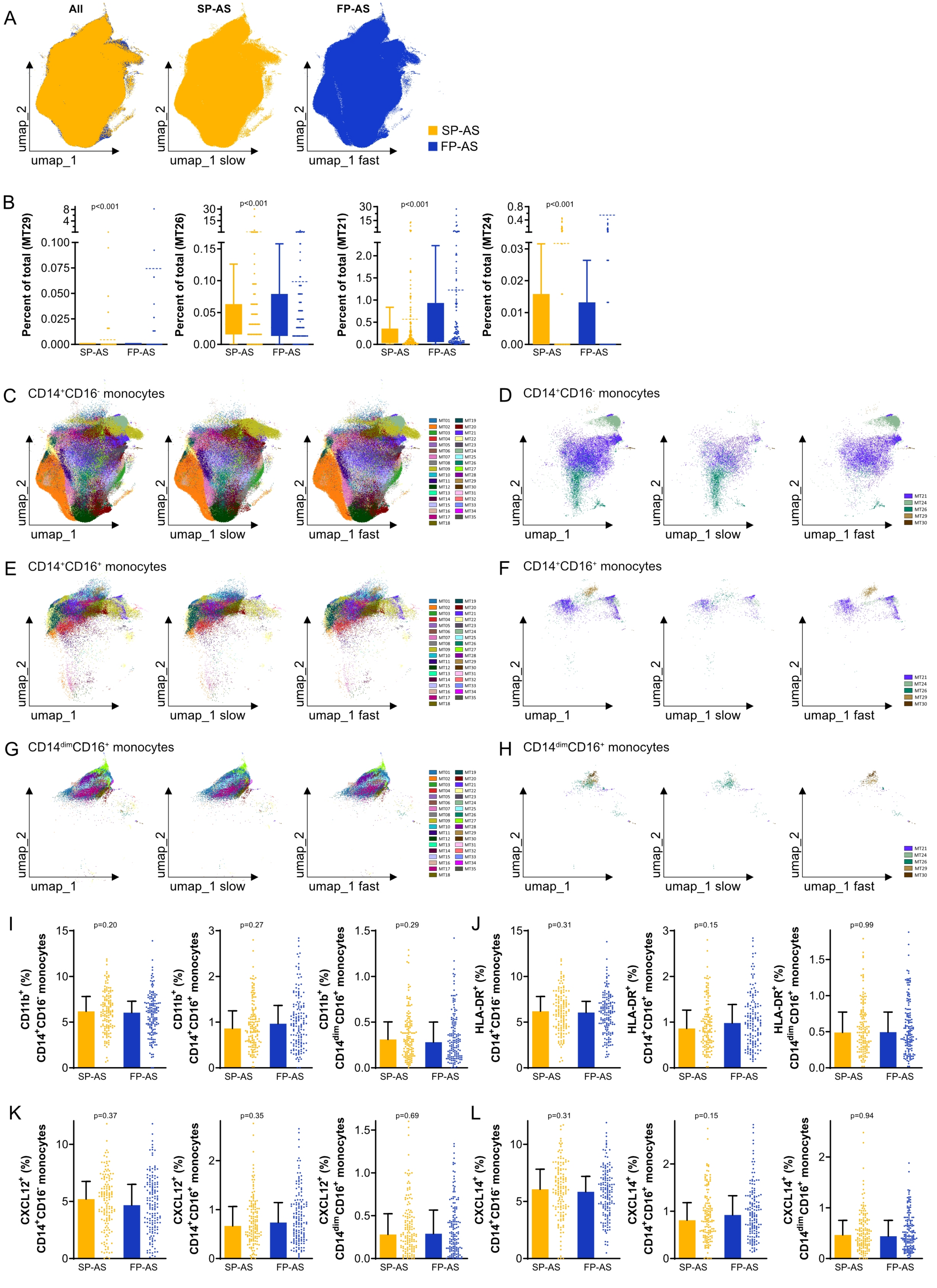

Supplemental Figure S6. UMAP and PhenoGraph analysis of human monocyte flow cytometry panel 1 and surface marker expression. (A) UMAP analysis of monocyte flow cytometry panel 1 of patients divided into slow (orange) and fast (blue) progressive AS with the left plot showing an overlay and the other two plots showing the individual patient groups. **(B)** Percentage of cells for each patient in the depicted clusters are shown as box plots divided into slow and fast progressive AS. Only significantly different clusters (abundancy) MT29, MT26, MT21 and MT 24 are presented. Plotted: Median ± interquartile range (IQR); Statistics: Mann-Whitney U test. **(C-H)** Analysis of monocyte subpopulations. PhenoGraph algorithm was used (nearest neighbors k=20) for unsupervised clustering. Left UMAP plots show an overlay of slow and fast progressive patients and the other two plots show the individual patient groups (middle = slow; right = fast) gated manually on $CD14^+$ monocytes **(C-D)**, intermediate $CD14^+CD16^+$ monocytes **(E-F)** and non-classical $CD16^+$ monocytes **(G-H)**. **(C, E, G)** show all clusters from PhenoGraph analysis whereas **(D, F, H)** show only significant different clusters. **(A-H)** Plots were generated using OMIQ data analysis software. **(I-L)** Marker expression on monocyte subsets after manual gating are shown for slow compared to fast progressive patients. Frequency (%) of following marker were evaluated for each monocyte subsets: **(I)** $CD11b^+$ monocytes, **(J)** $HLA\text{-}DR^+$ monocytes, **(K)** $CXCL12^+$ monocytes and **(L)** $CXCL14^+$ monocytes. **(I-L**) Flow cytometry data were analyzed by FlowJo. Plotted: Median ± interquartile range (IQR); Statistics: Mann-Whitney U test.

Supplemental Figure S7

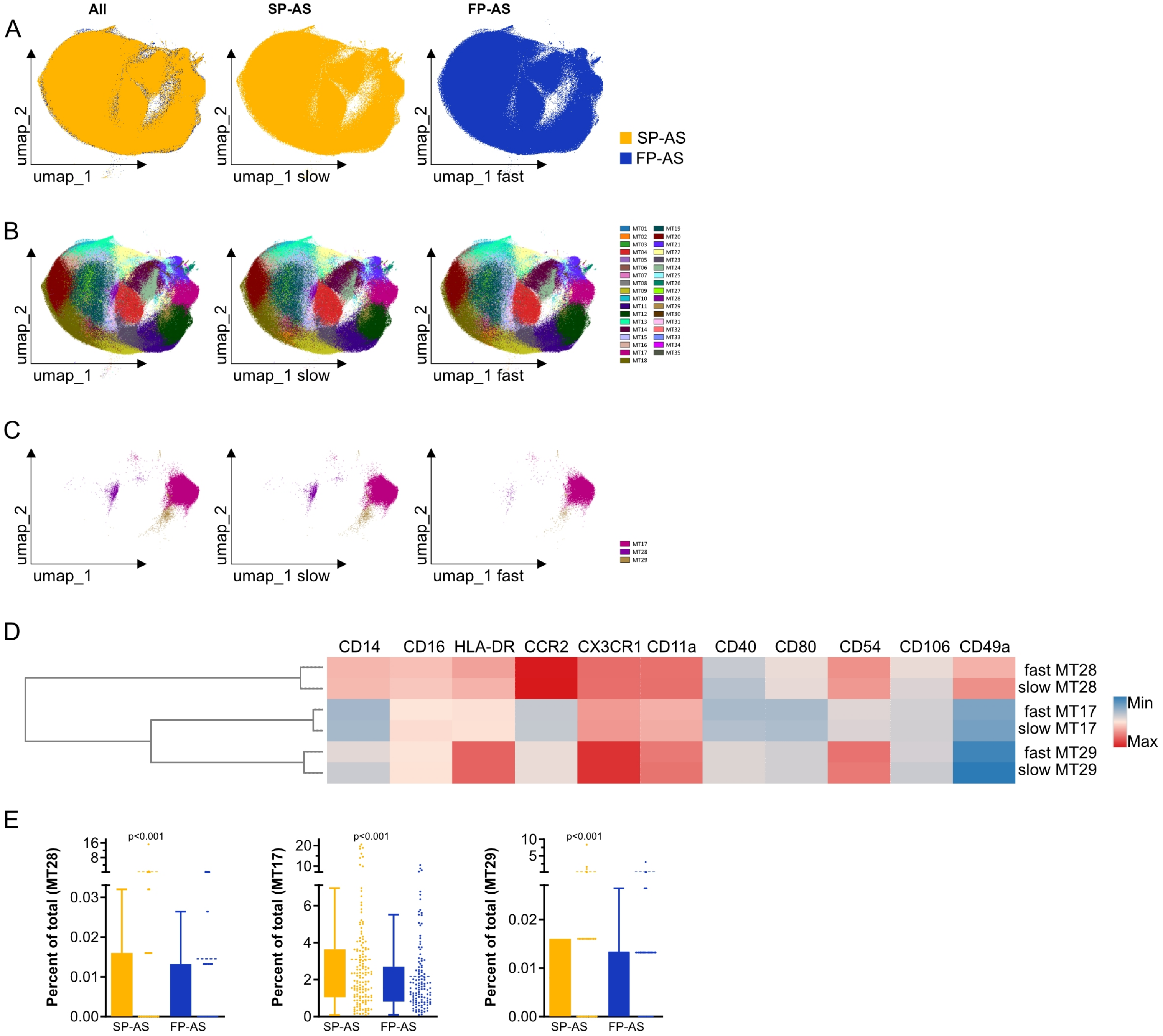

Supplemental Figure S7. Unsupervised gating strategy of human monocyte flow cytometry panel 2 and PhenoGraph clustering. (A) UMAP analysis of monocyte flow cytometry panel 2 of patients divided into slow (orange) and fast (blue) progressive AS with the left plot showing an overlay and the other two plots showing the individual patient groups. **(B-C)** Analysis of monocyte subpopulations. PhenoGraph algorithm was used (nearest neighbors k=20) for unsupervised clustering. Left UMAP plots show an overlay of slow and fast progressive patients and the other two plots show the individual patient groups (middle = slow; right = fast). **(B)** Plots show all clusters from PhenoGraph analysis whereas in **(C)** plots show only significant different clusters. **(D)** Heatmap of median expression of analyzed markers monocyte flow cytometry panel 2. Patients are divided into slow and fast progressive aortic stenosis and only significant different clusters MT28, MT17, and MT29 are shown. **(E)** Percentage of cells for each patient in the depicted clusters are shown as box plots divided into slow and fast progressive AS. Plotted: Median ± interquartile range (IQR); Statistics: Mann-Whitney U test. **(A-E)** Plots were generated using OMIQ data analysis software.

Supplemental Figure S8

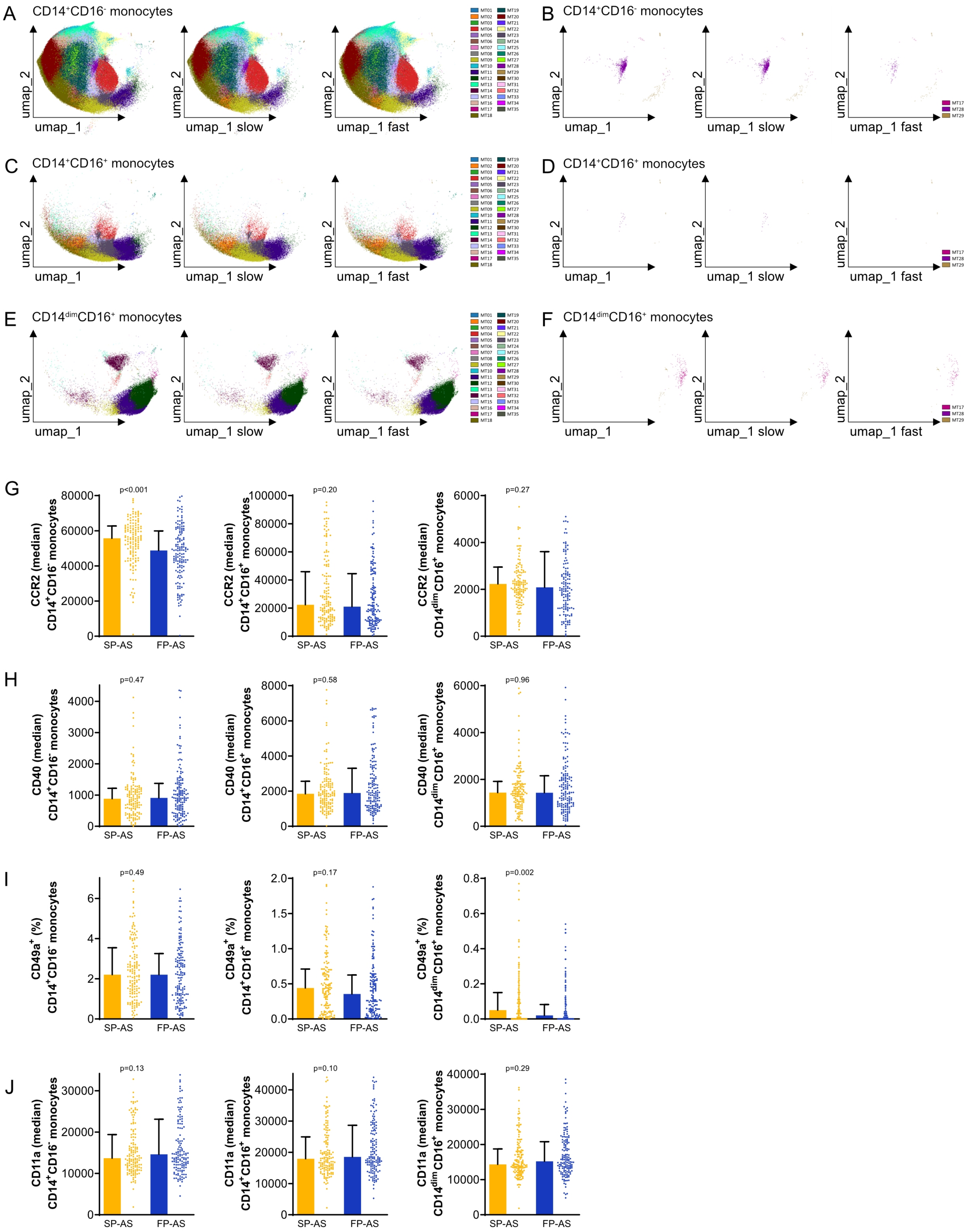

Supplemental Figure S8. PhenoGraph analysis of human monocyte flow cytometry panel 2 and surface marker expression. (**A-F**) PhenoGraph analysis of monocyte flow cytometry panel 2 of patients divided into slow and fast progressive AS with the left plot showing both and the other two plots showing the individual patient groups (middle = slow; right = fast) gated manually on CD14$^+$ monocytes **(A-B)**, intermediate CD14$^+$CD16$^+$ monocytes **(C-D)** and non-classical CD16$^+$ monocytes **(E-F)**. **(A, C, E)** show all clusters from PhenoGraph analysis whereas **(B, D, F)** show only significant different clusters. **(A-H)** Plots were generated using OMIQ data analysis software. **(G-J)** Marker expression of monocyte subsets after manual gating are shown for slow and fast progressive patients. Following marker were evaluated for each monocyte subsets: **(G)** median of CCR2 on monocytes, **(H)** median of CD40 on monocytes, **(I)** frequency (%) of CD49a$^+$ monocytes and **(J)** median of CD11a on monocytes. (**G-J**) Flow cytometry data were analyzed by FlowJo. Plotted: Median ± interquartile range (IQR); Statistics: Mann-Whitney U test.

Supplemental Figure S9

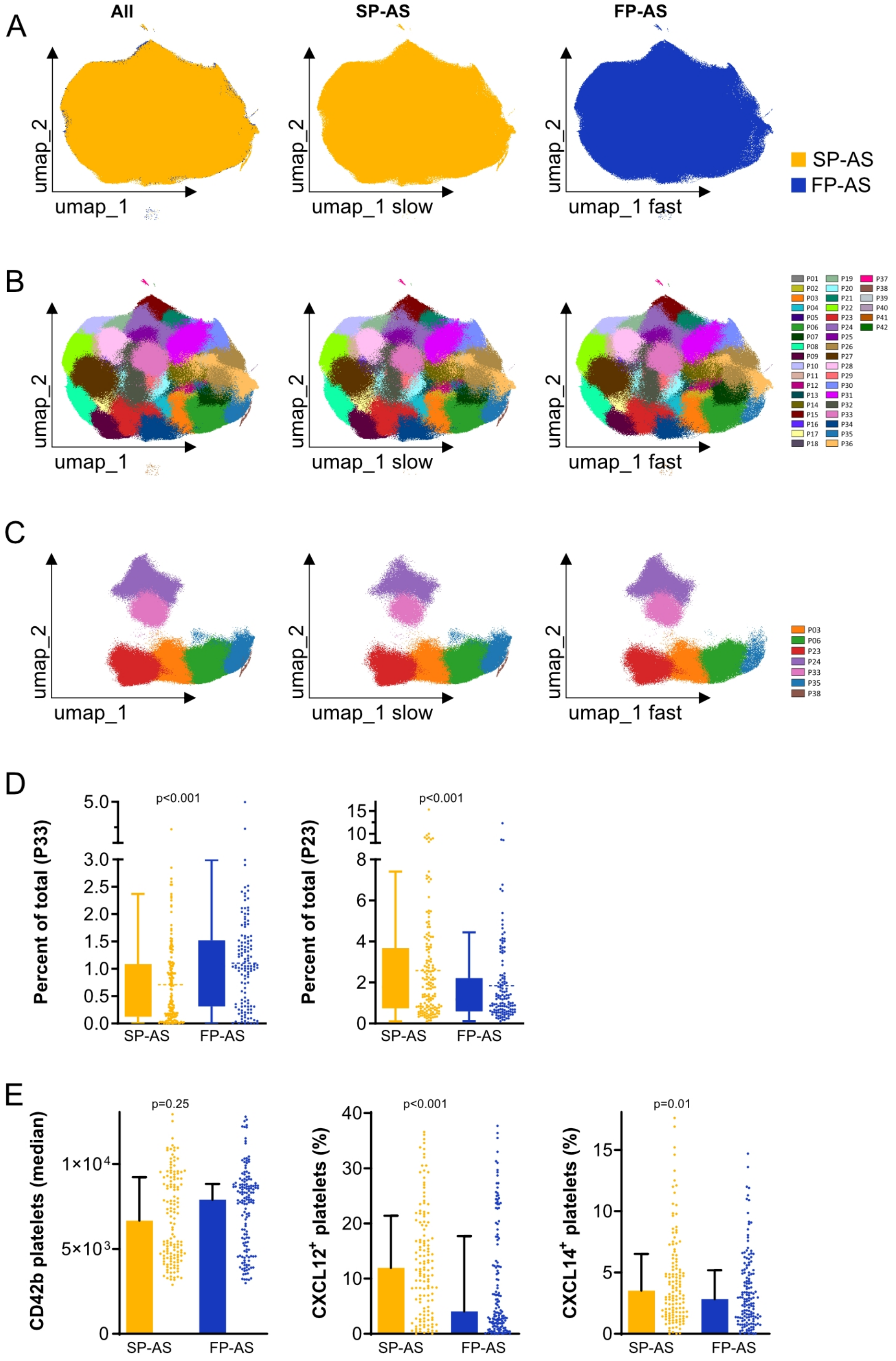

Supplemental Figure S9. UMAP analysis of human platelet flow cytometry panel and manual gating strategy and surface marker expression. (**A**) UMAP analysis of platelet flow cytometry panel of patients divided into slow (orange) and fast (blue) progressive AS with the left plot showing an overlay and the other two plots showing the individual patient groups. **(B)** Percentage of cells for each patient in the depicted clusters are shown as box plots divided into slow and fast progressive AS. Plotted: Median ± interquartile range (IQR); Statistics: Mann-Whitney U test. **(C-D)** PhenoGraph clustering with the left plot showing both and the other two plots showing the individual patient groups (middle = slow; right = fast). **(C)** Plots show all clusters manually gated platelets from PhenoGraph analysis whereas in **(D)** plots show only significant different clusters. **(A-D)** Plots were generated using OMIQ data analysis software. **(E)** Marker expression of platelets after manual gating are shown for slow and fast progressive patients. Following marker were evaluated: median of CD42b on platelets, frequency (%) of $CXCL12^+$ and $CXCL14^+$ of platelets. Flow cytometry data were analyzed by FlowJo. Plotted: Median ± interquartile range (IQR); Statistics: Mann-Whitney U test.

Supplemental Figure S10

A SP-AS

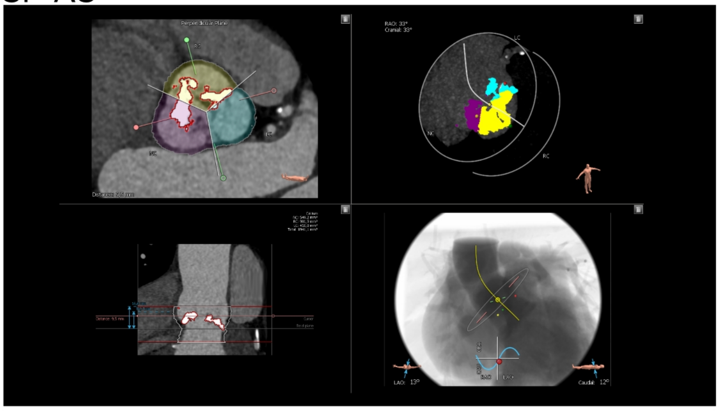

B FP-AS

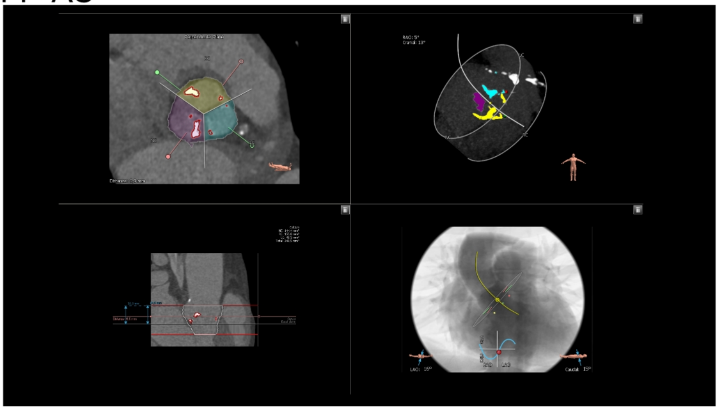

C

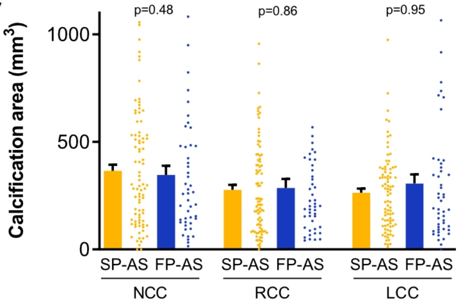

D

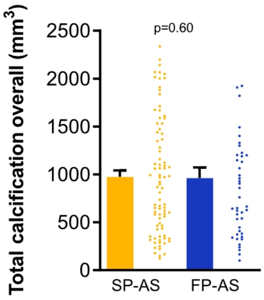

Supplemental Figure S10. Computed tomography (CT) scans of the aortic valve were not sufficient to morphologically discriminate between fast and slow progressive aortic stenosis.

(A) Representative CT scan of a patient with fast progressive aortic stenosis, inflammatory phenotype in immunohistology and low degree of calcification and mineralization. **(B)** Representative CT scan of a patient with slow progressive aortic stenosis, calcifying phenotype in immunohistology and high degree of calcification and mineralization. The 3mensio Valves software (PIE Medical Imaging, Maastricht, The Netherlands) was used for the analysis. First annulus plane was defined according to cusps insertion points. For the purposes of quantifying the degree of valvular calcification, the aortic root was separated into two regions along its double-oblique long axis: the leaflet area extended from annulus plane to the lower coronary artery and the left ventricular outflow tract (LVOT) from the annulus plane to 10 mm immediately below it. These regions were further divided into three distinct sectors across the annular plane, corresponding to the non-coronary (NC), left coronary (LC) and right coronary (RC) cusps. Calcification (mm^3) was measured with predefined 500-HU threshold with manual adjustment if necessary. This method was chosen over the standard Agatston method due to increased reproducibility. **(C-D)** There were no significant morphological differences in the CT scans of patients with slow (n=65) and fast (n=69) progressive aortic stenosis, neither regarding the degree of calcification in each single cusp (RCC, NCC, and LCC (**C**), nor the grade of total mineralization of the aortic valve (**D**). Plotted: Median ± interquartile range (IQR); Statistics: Mann-Whitney U test.

Supplemental Figure S11

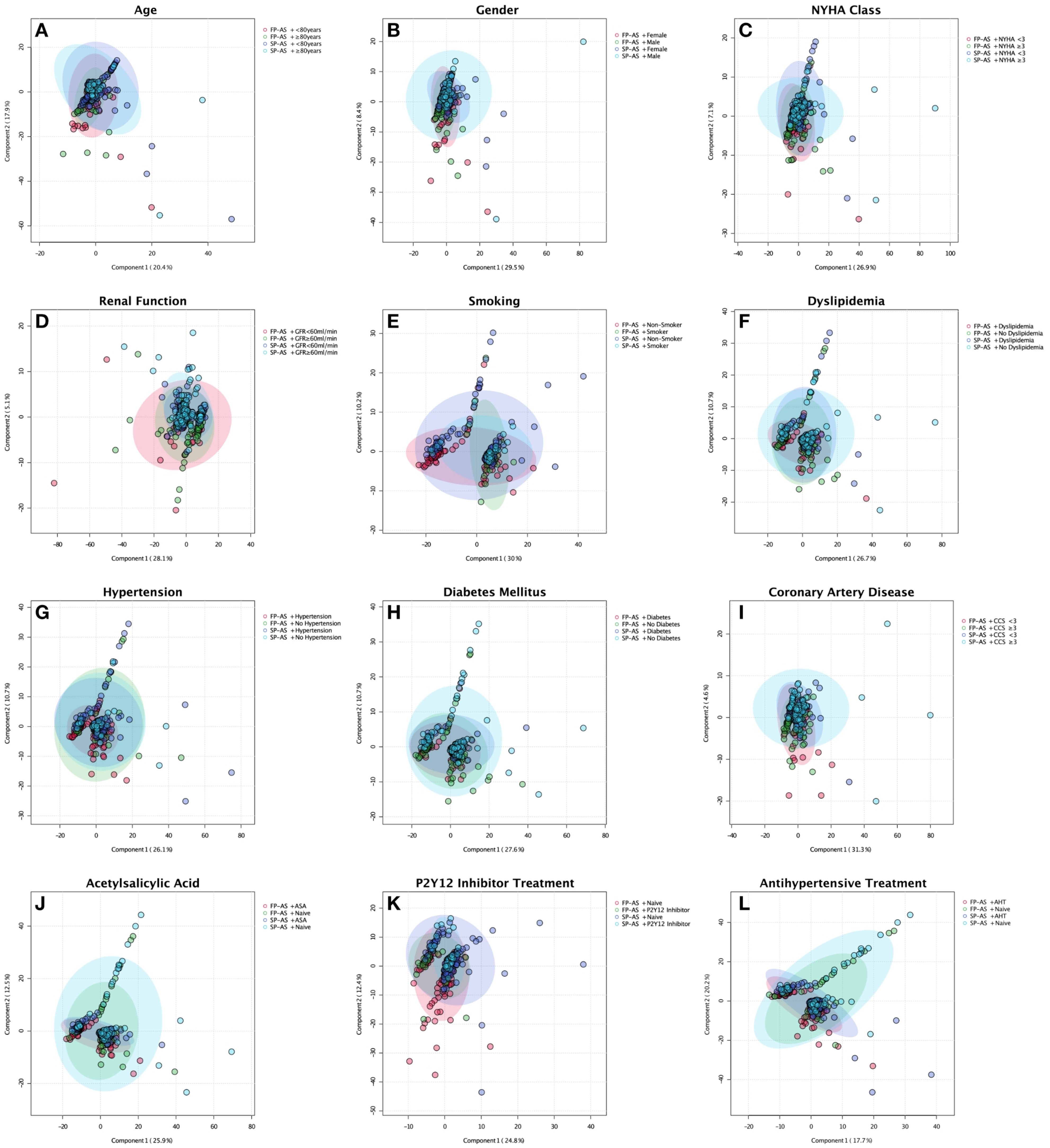

Supplemental Figure S11. Effect of cardiovascular risk factors (age, gender, diabetes mellitus type 2, smoking status, arterial hypertension, hyperlipidemia), comedication (acetylsalicylic acid, P2Y12 inhibitors, antihypertensive treatment), and comorbidities (heart failure defined by NYHA class, symptomatic coronary artery disease, chronic kidney disease) on the progression rate of aortic stenosis (AS). PLS-DA and OPLS-DA analysis was performed to assess the impact of clinical parameters on the progression of aortic stenosis, defined as fast (ΔVmax ≥ 0.45 m/sec/year) and slow (ΔVmax < 0.45 m/sec/year) progressive AS. The four subgroups (fast progressive AS ± possible confounder / slow progressive AS ± possible confounder) are coloured and labelled as depicted in the legend above. A homogenous spreading of the subgroups **(A–L)** implements a minor influence of assessed risk factors, treatment and comorbidities on the progression rate of AS. The comparison of fast and slow progressive AS is illustrated for each analyzed parameter. Cumulative R2 and Q2 were derived from 5-fold cross-validation. **(A)** PLS-DA analysis of patient age indicates that age does not significantly impact the progression of AS [R2= 0.741, Q2= 0.105]. **(B)** Gender was also not associated with disease progression in PLS-DA analysis [R2= 0.760, Q2= 0.116]. **(C)** PLS-DA analysis of NYHA class due to concomitant impaired left ventricular function and heart failure was not associated with changes of peak velocity ΔVmax and progression rate of AS [R2= 0.836, Q2= 0.250]. **(D)** Chronic kidney disease defined by impaired glomerular filtration rate (GFR < 60mL/min) was not significantly associated with the progression rate of AS in PLS-DA analysis [R2= 0.957, Q2= 0.393]. **(E)** PLS-DA analysis of the smoking status indicates that current smoking does not significantly impact the progression of AS [R2= 0.796, Q2= 0.294]. **(F)** Elevated total cholesterol and LDL levels defined as hyperlipidaemia were not associated with progression of aortic stenosis in our cohort shown by PLS-DA analysis [R2= 0.728, Q2= 0.241]. **(G)** Presence of arterial hypertension does not significantly affect the progression rate of AS in PLS-DA analysis [R2= 0.800, Q2= 0361]. **(H)** PLS-DA analysis shows that diabetes mellitus type 2 does not significantly influence the progression rate of aortic stenosis shown for all four subgroups [R2= 0.771, Q2= 0.275]. **(I)** Presence of symptomatic coronary artery disease defined by either of slight to moderate

(CCS<3) and severe angina pectoris (CCS≥3) did not significantly impact progression rate of AS in PLS-DA analysis [R2=1.108, Q2= 0.620]. **(J)** Therapy with acetylsalicylic acid was no relevant confounding factor in OPLS-DA analysis [R2= 0.826, Q2= 0.417]. **(K)** Antiplatelet therapy with P2Y12-inibitors did not significantly influence progression rate of AS in OPLS-DA analysis [R2= 1.075, Q2= 0.701]. **(L)** Antihypertensive treatment did not significantly influence progression rate of AS in OPLS-DA analysis [R2= 0.106, Q2= 0.588].

Supplemental Figure S12

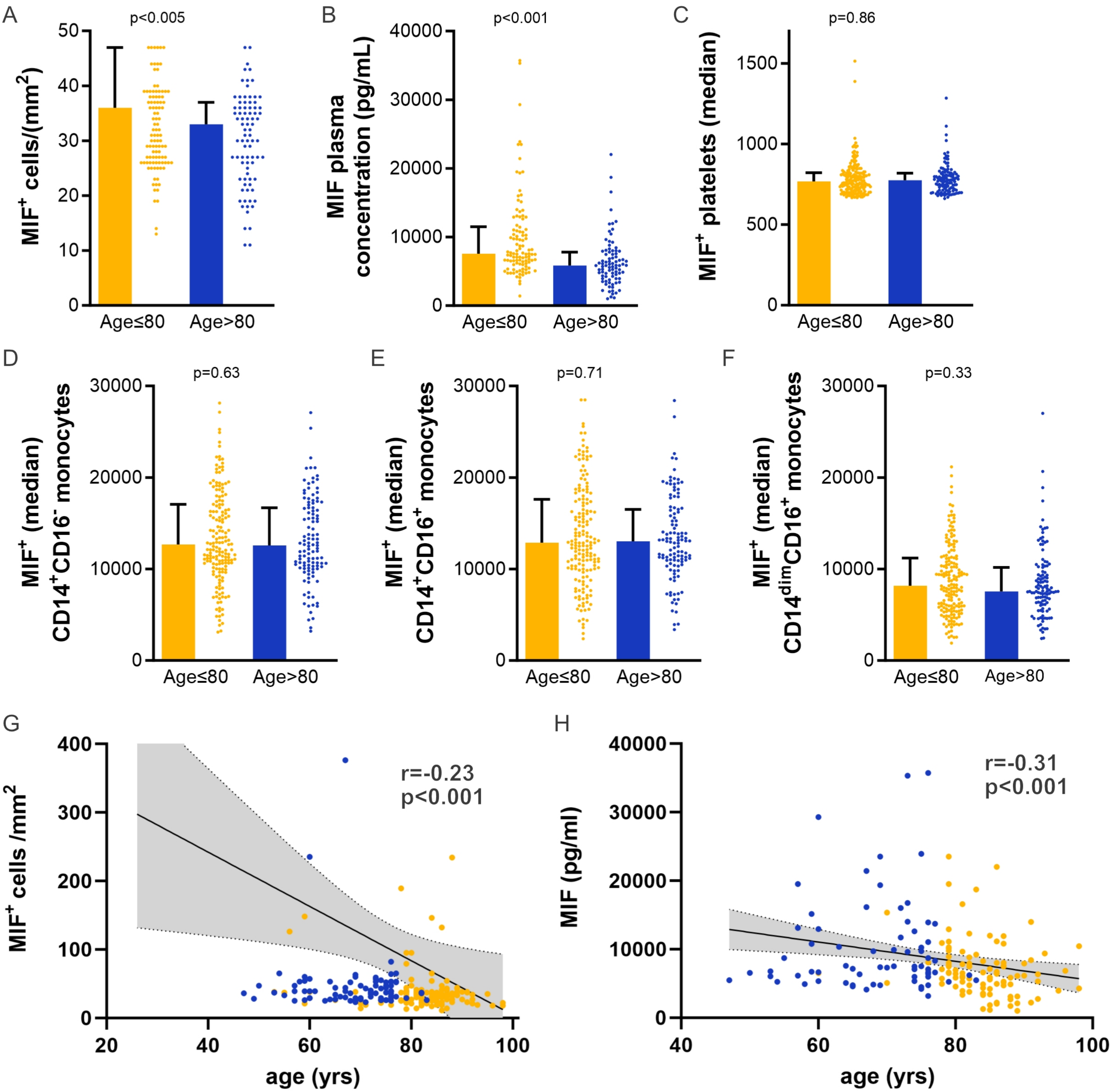

Supplemental Figure S12. Local and systemic MIF expression is enhanced in younger patients (age ≤ 80 years) suffering from aortic valve stenosis. (A) Patients with an age ≤ 80 years show a higher number of MIF^+ cells within the aortic valve tissue compared to patients older than 80 years (n=218, p<0.005). **(B)** MIF plasma concentration is significantly higher in younger patients with aortic stenosis (n=193, p<0.001). Intracellular MIF expression (median) in **(C)** platelets, **(D)** classical, **(E)** intermediate, and **(F)** non-classical monocyte subsets was not different in AS patients over 80 years compared to patients ≤ 80 years (n=292, p>0.05, respectively). Plotted: Median ± interquartile range (IQR); Statistics: Mann-Whitney U test. Spearman´s correlation analysis to evaluate associations of age with **(G)** local valvular and **(H)** systemic plasmatic MIF expression. Spearman´s correlation analysis of age and **(G)** number of MIF^+ cells within the aortic valve tissue (n=218, r=-0.23, p<0.001) as well as **(H)** MIF plasma concentration (n=193, r=-0.31, p<0.001) confirmed an association of age with these parameters.

Supplemental Figure S13

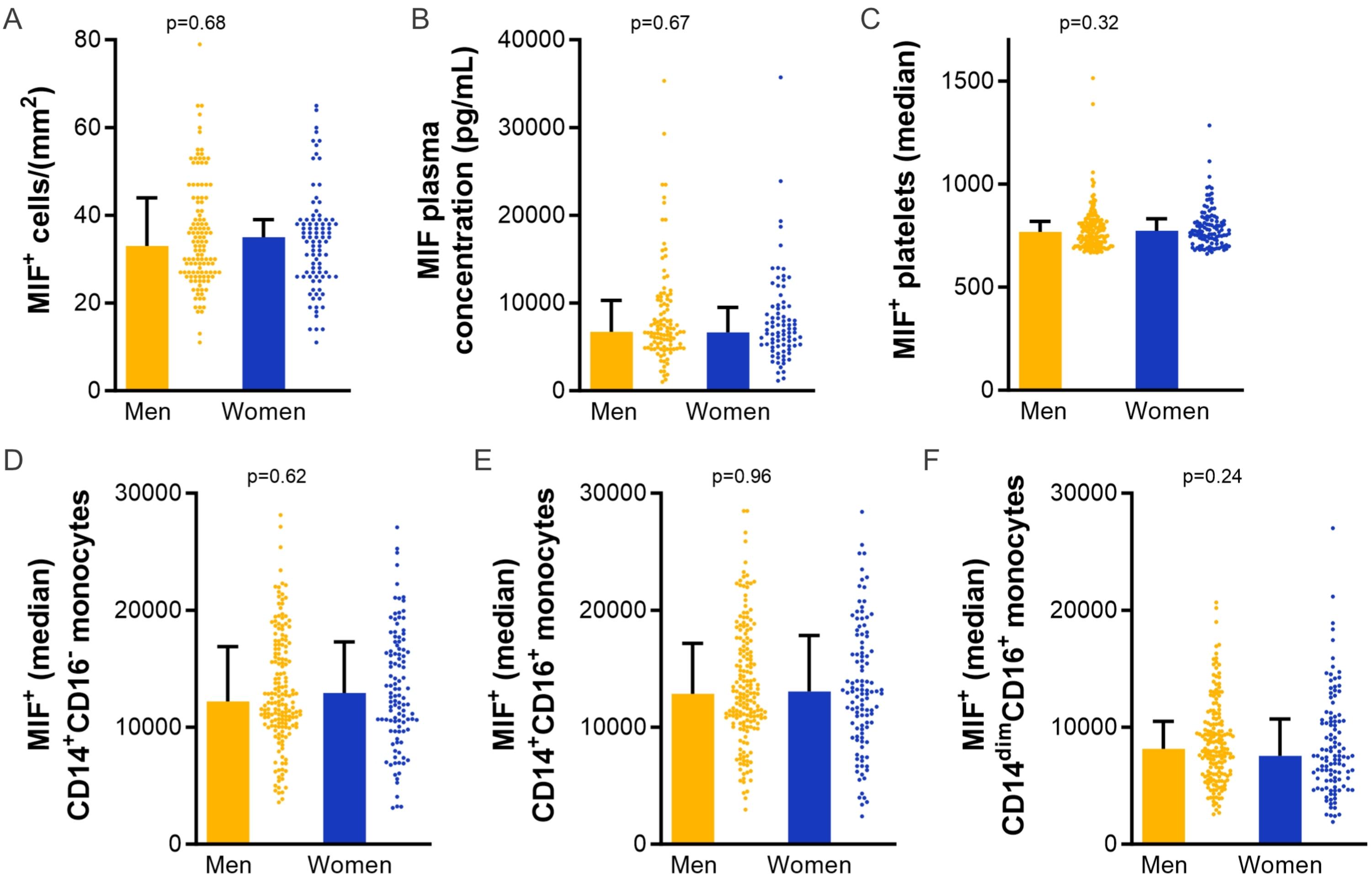

Supplemental Figure S13. Local and systemic MIF expression was not associated with gender-specific differences in patients with aortic valve stenosis. (A) Numbers of MIF^+ cells within the aortic valve tissue were not different in men compared to women (n=218, p=0.68). **(B)** MIF plasma concentration showed no gender-specific differences (n=193, p=0.67). Intracellular MIF expression (median) in **(C)** platelets, **(D)** classical, **(E)** intermediate, and **(F)** non-classical monocyte subsets was not different among male and female patients with AS (n=292, p=0.32, p=0.62, p=0.96, p=0.24, respectively). Plotted: Median ± interquartile range (IQR); Statistics: Mann-Whitney U test.

Supplemental Figure S14

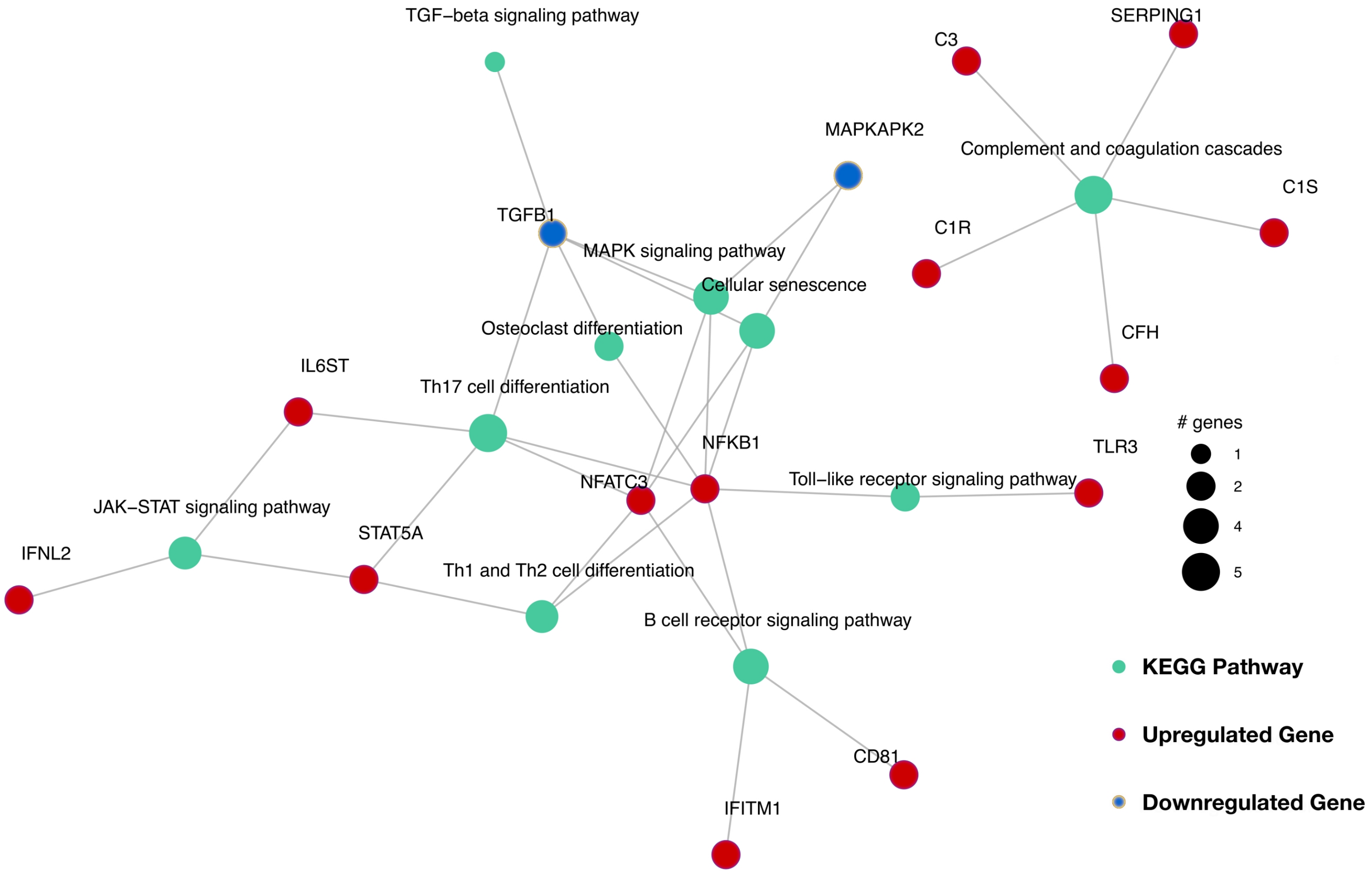

Supplemental Figure S14. Term-gene-graph illustrating subnetworks and regulations in FP-AS and SP-AS.

A term-gene-graph highlights subnetworks and regulations of significantly ($p<0.05$) altered genes and the referring Kyoto Encyclopedia of Genes and Genomes (KEGG) pathways in patients with FP-AS when compared to SP-AS.

Supplemental Figure S15

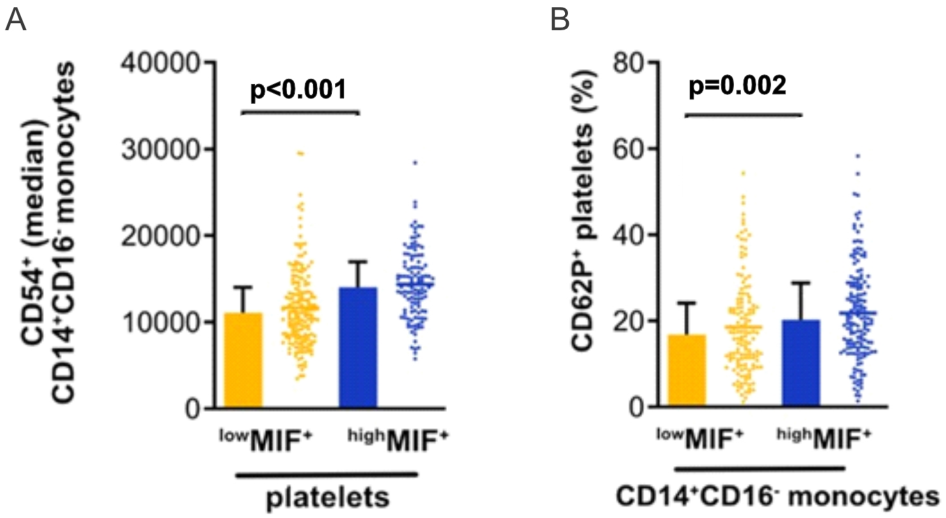

Supplemental Figure S15. Analysis of MIF^{high} platelets and MIF^{high} monocytes reveals an association with platelet and monocyte activation (A) Patients with high MIF levels in their platelets show an increased expression of CD54 (ICAM-1) on their monocytes indicating monocyte activation with increased adhesion markers in this patient group ($p<0.001$). **(B)** High MIF content in monocytes is associated with increased CD62P+ expression in platelets indicating an elevated activation status of platelets among this group ($p=0.002$).

Supplemental Table I: Major Resources Table

<u>Antibodies for Flow cytometry</u>

Target antigen	Clone	Company	Catalog #
CD3 BV510	OKT3	BioLegend	317332
CD15 BV510	W6D3	BioLegend	323028
CD19 BV510	HIB19	BioLegend	302242
CD20 BV510	2H7	BioLegend	302340
CD56 BV510	HCD56	BioLegend	318340
CD14 FITC	M5E2	BioLegend	301804
CD16 BV711	3G8	BioLegend	302044
HLA-DR PerCP Vio770	REA805	Miltenyi	130-111-793
CD197 (CCR7) BV785	G043H7	BioLegend	353230
CD11b BV605	ICRF44	BioLegend	301332
CD62L PECy7	DREG-56	BioLegend	304822
CXCR7 BV421	10D1	BD	566233
CXCR4 BV650	12G5	BD	740599
CD192 (CCR2) BV605	K036C2	BioLegend	357214
CX3CR1 PE/Dazzle 594	2A9-1	BioLegend	341624
CD54 PE	REA266	Miltenyi	130-120-711
CD106 APC	STA	BioLegend	305810
CD49a PECy7	TS2/7	BioLegend	328312
CD11a BV650	HI1 11	BD	563934
CD40 BV421	5C3	BioLegend	334332
CD80 BV785	2D10	BioLegend	305238
CD41 PaBl	HIP8	BioLegend	303714
CD31 BV711	WM59	BioLegend	303136
CD61 FITC	VI-PL2	BioLegend	336404
CD42b PerCP/Cy5.5	HIP1	BioLegend	303918
CD62P PECy7	AK4	BioLegend	304922
MIF AF594	932606	R&D Systems	IC2891T-100UG
CXCL14 AF647	MM0213-12B24	Novus Biological	NBP2-12224AF647
CXCL12 PE	79018	R&D Systems	IC350P

Other reagents for flow cytometry

Reagent	Company	Order number
RBC Lysis Buffer (10X)	BioLegend	420302
eBioscience™ Foxp3 / Transcription Factor Staining Buffer Set	Thermo Fisher	00-5523-00
IgG from human serum	MERCK	I4506-10MG
Dulbeccos Phosphatgepufferte Kochsalzlösung	MERCK	D8537
Gibco™ Fetal Bovine Serum, qualified, heat inactivated, United States	Fisher scientific	11580516

Immunohistochemistry antibodies

Antigen	Company	Order number
Anti- SDF- antibody	Abcam	Ab9797
Goat anti human IgG H&L	Abcam	Ab6858
Anti- MIF antibody	Abcam	Ab55445
Anti-GPCR RDC1/CXCR-7 antibody	Abcam	Ab72100
Human CXCR4 antibody	R&D Systems	MAB172-100
Normal mouse IgG	Santa Cruz	Sc-2025
Goat Anti-Human IgG H&L (HRP) (ab6858)	Abcam	Ab6858
CD14 (UCH-M1)	Santa Cruz	Sc-1182
CD16 (DJ130c)	Santa Cruz	Sc-20052
CD68 (KP1)	Santa Cruz	Sc-20060
CD42b (E-8)	Santa Cruz	Sc-271171
Anti-Gremlin-1	Abcam	Ab189267
Anti-Gremlin-1	Santa Cruz	Sc-18274
Anti-CXCR3	Abcam	Ab125255
Anti-CD3	Abcam	Ab16669
Anti-BMP-7	Bioss	Bs-2242R-Biotin
Anti-BMP-4	GeneTex	GTX63501
Anti-BMP-2	Bioss	bs-1012R
Anti-CD42b	Santa Cruz	Sc-7070
Anti-TGF-ß1	Santa Cruz	Sc-146-G
Anti-CXCL11	Abcam	ab9955
Rabbit Immunoglobulin Fraction (Solid-Phase Absorbed)	Agilent/ Dako	X0936

Immunohistological staining kits

Reagent	Company	Order number
LSAB2 Kits, Universal	Agilent/ Dako	K067511-2
DAB+ Chromogen, Liquid	Agilent/ Dako	K346811-2
Streptavidin (Conjugate) Streptavidin/HRP, Immunohistochemistry/ISH/Blot/ELISA, 1 mL	Agilent/ Dako	P039701-2
Proteinblock, Serum- free, Ready-to-use	Agilent/ Dako	X090930-2
Goat Serum (Normal, for Immunohistochemistry)	Agilent/ Dako	X090710-8
Rabbit Immunoglobulin Fraction (Normal)	Agilent/ Dako	X090302-8
Anti-Goat HRP-DAB Cell & Tissue Staining Kit	R&D Systems	CTS008
Anti-Mouse HRP-DAB Cell & Tissue Staining Kit	R&D Systems	CTS002

Histological staining kits

Reagent	Company	Order number
Movat Pentachrom	DIAPATH /Weinkauf Medizintechnik	010247
Von Kossa	DIAPATH /Weinkauf Medizintechnik	010241
Mayers Hämalaunlösung	Merck	109249

Other histological materials

Reagent	Company	Order number
Embedding cassettes Macrosette™	ROTH	EE16.1
Accessories Sponges for embedding cassettes	ROTH	TT56.1
Staining chamber StainTray™ black lid, for 20 microscopie slides	ROTH	HA51.1
Staining through (Glass)	ROTH	H554.1
Staining trough ROTILABO®, blue	ROTH	HA44.1
Formaldehyd-solution 4 % buffered , 1 L	Fischar	27279
Hydrogen peroxide	ROTH	CP26.4
ROTI®Histol, 5 l	ROTH	6640.5
ROTI®Histokitt, 100 ml	ROTH	6638.1
Super PAP Pen Liquid Blocker new	Science Services	N71310-N
PBS Tablets	Thermo Fisher Scientific	18912014
Tween 20	SigmaAldrich	8221840500

ELISA

Reagent	Company	Order number
LEGEND MAX™ Human Active MIF ELISA Kit	BioLegend	438407
Human MIF ELISA Kit	Ray biotech, inc. norcross, USA	P14174
Human/Mouse/Rat/Porcine/Canine TGF-beta 1 Quantikine ELISA	R&D/ biotechne	DB100B
